# A Dose-Dependent Association between Alcohol Consumption and Incidence of Proteinuria and Low Glomerular Filtration Rate: A Systematic Review and Meta-Analysis of Cohort Studies

**DOI:** 10.3390/nu15071592

**Published:** 2023-03-25

**Authors:** Ryohei Yamamoto, Qinyan Li, Naoko Otsuki, Maki Shinzawa, Makoto Yamaguchi, Minako Wakasugi, Yasuyuki Nagasawa, Yoshitaka Isaka

**Affiliations:** 1 Health and Counseling Center, Osaka University, Toyonaka 560-0043, Japan; 2 Department of Nephrology, Graduate School of Medicine, Osaka University, Suita 565-0871, Japan; 3 Department of Nephrology and Rheumatology, Aichi Medical University, Nagakute 480-1195, Japan; 4 Department of Inter-Organ Communication Research, Graduate School of Medical and Dental Sciences, Niigata University, Niigata 951-8510, Japan; 5 Department of General Internal Medicine, Hyogo Medical College, Nishinomiya 663-8501, Japan

**Keywords:** alcohol consumption, chronic kidney disease, cohort study, dose-dependent association, glomerular filtration rate, meta-analysis, proteinuria, systematic review

## Abstract

Previous cohort studies have reported conflicting associations between alcohol consumption and chronic kidney disease, characterized by proteinuria and low glomerular filtration rate (GFR). This systematic review, which included 14,634,940 participants from 11 cohort studies, assessed a dose-dependent association of alcohol consumption and incidence of proteinuria and low estimated GFR (eGFR) of <60 mL/min/1.73 m^2^. Compared with non-drinkers, the incidence of proteinuria was lower in drinkers with alcohol consumption of ≤12.0 g/day (relative risk 0.87 [95% confidence interval 0.83, 0.92]), but higher in drinkers with alcohol consumption of 36.1–60.0 g/day (1.09 [1.03, 1.15]), suggesting a J-shaped association between alcohol consumption and the incidence of proteinuria. Incidence of low eGFR was lower in drinkers with alcohol consumption of ≤12.0 and 12.1–36.0 than in non-drinkers (≤12.0, 12.1–36.0, and 36.1–60.0 g/day: 0.93 [0.90, 0.95], 0.82 [0.78, 0.86], and 0.89 [0.77, 1.03], respectively), suggesting that drinkers were at lower risk of low eGFR. In conclusion, compared with non-drinkers, mild drinkers were at lower risk of proteinuria and low eGFR, whereas heavy drinkers had a higher risk of proteinuria but a lower risk of low eGFR. The clinical impact of high alcohol consumption should be assessed in well-designed studies.

## 1. Introduction

Chronic kidney disease (CKD) characterized by proteinuria and low glomerular filtration rate (GFR) [1] is a major global health problem [2] and an enormous economic burden [3], because patients with CKD are at higher risk of end-stage kidney disease (ESKD) [4], cardiovascular disease (CVD) [5,6], and all-cause mortality [6]. Multiple studies have identified modifiable lifestyle factors as risk factors for incidence of CKD, including smoking [7], physical inactivity [8,9], sedentary behavior [10], short sleep duration [11,12], and unhealthy dietary behaviors, including poor dietary patterns [13], breakfast skipping [14], and low vegetable consumption [15,16]. To establish an effective CKD prevention strategy, the association between the modifiable lifestyle factors and CKD should be clarified extensively.

Alcohol consumption, a major global risk factor of attributable disability-adjusted life-years [2], is a potential modifiable lifestyle factor for CKD. Some recent systematic reviews have reported conflicting results of an association between alcohol consumption and incidence of CKD. A systematic review by Chinese researchers, which included 268,723 participants from 15 cohort studies, suggested a U-shape association between alcohol consumption and the incidence of a wide variety of kidney damages, including CKD, ESKD, declined GFR, and proteinuria [17]. Another systematic review by different Chinese researchers, which included 514,148 participants from 25 cohort studies, reported that drinkers were at a lower risk of incidence of CKD, ESKD, proteinuria, or eGFR decline [18]. The findings of these systematic reviews should be interpreted with caution because they included studies with various kidney outcomes. After these systematic reviews, several large cohort studies, which included 14,190,878 Korean [19], 177,572 Japanese [20], and 26,788 Japanese [21] adults, identified high alcohol consumption as a significant predictor of incidence of proteinuria.

This systematic review, which included 14,634,940 participants from 11 cohort studies, aimed to assess a dose-dependent association between alcohol consumption and major outcomes of CKD, namely, incidence of proteinuria and low eGFR of <60 mL/min/1.73 m^2^, reflecting the results of recent large cohort studies. To study the clinical impact of high alcohol consumption on the outcomes precisely, we included the cohort studies with the lower boundary of the highest alcohol consumption category of >12 g/day. The findings of the present study update epidemiological evidence of the association between high alcohol consumption and CKD.

## 2. Materials and Methods

The protocol for this systematic review and meta-analysis, registered in PROSPERO (CRD42023388228), adheres to the meta-analysis of observational study in epidemiology (MOOSE) reporting guidelines [22].

### 2.1. Literature Search and Selection Criteria

PubMed and Web of Science were searched between January 2000 and December 2022, to identify relevant cohort studies that investigated an association between alcohol consumption and the incidence of proteinuria or low eGFR of <60 mL/min/1.72 m^2^. The search strategy used is described in Appendix A in detail. Briefly, search terms included “proteinuria” or “glomerular filtration rate” with “alcohol,” followed by terms to exclude non-cohort studies. In addition, we searched the reference lists of included publications and relevant reviews. The search was limited to publications available in the English language.

To assess a dose-dependent association between alcohol consumption, especially moderate to high consumption of >12 g/day, and incidence of the outcomes, we included studies in this review if a study (i) was a prospective or retrospective cohort study, (ii) measured the baseline alcohol consumption stratified by at least 3 categories, including non-/rare drinkers as a reference group, and current drinkers with at least 2 levels of alcohol consumption as exposure groups, (iii) had the highest alcohol consumption category with the lower boundary of >12 g/day, (iv) measured incidence of proteinuria or low eGFR of <60 mL/min/1.73 m^2^ during the follow-up period, and (v) were published in the English language.

All records retrieved from the literature search were assessed by two reviewers (RY and QL) independently for inclusion, using a web app, Rayyan (Rayyan Systems, Inc. Cambridge, MA, USA. Available online: https://rayyan.ai) [23]. Full texts of potentially eligible studies were then reviewed to determine their final eligibility. Any disagreements between two reviewers were resolved through consensus.

### 2.2. Data Extraction and Quality Assessment

Two reviewers (RY and QL) independently extracted the following information from each study: lead author, study name, publication year, study location, numbers of participants and cases with outcomes, follow-up duration, age and eGFR of participants, male proportion, prevalence of diabetes and hypertension, alcohol consumption category (g/day or drinks/day), and multivariable-adjusted hazard ratios of each alcohol consumption category, and covariates used in statistical analyses. If more than one multivariable-adjusted model was reported in a study, the one with the largest number of adjusted variables was extracted.

We used the Newcastle-Ottawa quality assessment scale (NOS) to assess the methodological quality of 12 publications of 11 studies [24]. The NOS includes 8 items of 3 domains: (i) selection, including representativeness of the exposed cohort (0 or 1 score), selection of non-exposed (0 or 1), and ascertainment of exposed (0 or 1); (ii) comparability of cohorts on the basis of the design or analysis (0, 1, or 2); and (iii) outcome, including assessment of outcome (0 or 1), follow-up length long enough for outcome to occur (0 or 1), and adequacy of follow-up of cohorts (0 or 1). A study is considered of good quality if the total score is at least 7/9 [25]. Two reviewers (RY and QL) independently conducted quality assessment of the included studies. Disagreements were resolved by discussion between the two reviewers.

### 2.3. Statistical Analysis

If the mean or median values of baseline age, body mass index, and eGFR of all participants were not reported, we used the following equations to calculate the mean value of all participants. If the mean value of each alcohol consumption category was reported,

Mean_all_ = Σ (Mean_category_ × N_category_) ÷ Σ N_category_

where Mean_all_ and Mean_category_ are the mean value of all participants and participants with each alcohol consumption category, respectively, and N_category_ is the number of participants in each alcohol consumption category. If the median value with the interquartile range of each alcohol consumption category was reported, we estimated the mean value of each alcohol consumption category using the following equation before calculating Mean_all_:

Mean_category_ = (Q1_category_ + Q3_category_ + Median_categry_) ÷ 3

where Q1_category_ and Q3_category_ are its first and third quartiles, respectively, and Median_category_ is its median value [26].

To assess a dose-dependent association between alcohol consumption (g/day) and the outcomes, we assigned the midpoint of the lower and upper boundaries of each alcohol consumption category as its representative value (Table 1). If the upper boundary was open-ended, 1.2 times its lower boundary was assigned as a representative value of the category [27]. A representative value of occasional drinkers was set at half a representative value of the adjacent category. If a unit of alcohol consumption was drinks/day (or drinks/week), it was converted to g/day (or g/week), according to a standard serving size of an alcoholic beverage in the study country, which is equivalent to 20 g of alcohol in Japan, 12 or 14 [28] g in the US and Canada, 8 g in the UK and Ireland, and 10 g in Australia [29]. Non-reference categories were divided into four groups of ≤12.0, 12.1–36.0, 36.1–60.0, and >60.0 g/day, based on the representative value of each alcohol consumption category. We pooled the relative risk (RR) estimates and their 95% confidence intervals of alcohol consumption for ≤12.0, 12.1–36.0, 36.1–60.0, and ≥60.0 g/day (vs. non-, rare, or never drinkers) with inverse weighting using the DerSimonian-Laird random-effects model to allow for between-study heterogeneity. If former drinkers were categorized separately from non-drinkers in a study, they were excluded from the present meta-analyses. Hazard ratios and odds ratios were considered as surrogate measures of RRs. Statistical heterogeneity among the studies was measured using the I^2^ statistic. I^2^ ≥ 50% suggested substantial heterogeneity. Publication bias was assessed by a visual inspection of funnel plot and by Egger’s statistical tests [30]. We considered a *p*-value <0.05 to be evidence of small study effects.

To examine a potential nonlinear association between alcohol consumption and the outcomes, we performed a 2-stage random-effects dose-response meta-analysis [31] with the use of restricted cubic splines with 4 knots at fixed percentiles (5%, 35%, 65%, and 95%) of the distribution, including cohort studies which reported the number of participants with incidence of outcomes. The first stage of the meta-analysis estimated the dose-response association between alcohol consumption and the log RRs in each included study. The study specific estimates were then combined in the second stage of this meta-analysis.

All analyses were conducted using Stata version 17.0 (StataCorp LLC, College Station, TX, USA) and R version 4.2.1 (The R Foundation for Statistical Computing, https://www.r-project.org). A *p*-value < 0.05 was considered statistically significant, unless otherwise specified.

**Table 1:** Alcohol consumption categories and outcomes in 12 publications from 11 cohort studies.

Author, Country, Year, Age, Sex	Alcohol Consumption (Representative g/Day)	*N*	Outcomes	Covariates
PHS [32]	≤1 drink/week (0.0)	4259	eGFR * < 55	Age, BMI, hypertension, diabetes, hypercholesterolemia, CVD, smoking, physical activity, parental CVD, RCT assignment
USA, 2005	2–4 drinks/week (6.0)	2582		
Age	5–6 drinks/week (11.0)	1474		
Men	≥7 drinks/week (16.8)	2708		
Yamagata [33]	Never drinkers (0.0)	88,934 †	eGFR < 60	Age, BMI, hypertension, IGT, diabetes, TCHO, HDL-C, TG, proteinuria, hematuria, smoking
Japan, 2007	Occasional drinkers (5.0)	10,036 †		
Age ≥ 40 years	Ethanol < 20 g/day (10.0)	22,112 †		
Men & women	Ethanol > 20 g/day (24.0)	2632 †		
ILSA [34] Italy, 2011 Age 65–84 years Men & women	Abstainers (0.0)	615 ‡	eGFR < 60	Age, BMI, hypertension, diabetes, TCHO, hyperlipidemia, fibrinogen, smoking, education level
	Former	673 ‡		
	<12 g/day (6.0)	819 ‡		
	12–24 g/day (18.0)	665 ‡		
	25–47 g/day (36.0)	413 ‡		
	≥48 g/day (57.6)	219 ‡		
Nagai [35] Japan, 2013 Age ≥40 years Men §	Non-drinkers (0.0)	26,232	Proteinuria ≥ 1+	Age, BMI, hypertensin, diabetes, TCHO, HDL-C, TG, eGFR, smoking
	Occasional drinkers (5.0)	12,019		
	Ethanol <20 g/day (10.0)	39,135		
	Ethanol >20 g/day (24.0)	4468		
Kansai Healthcare [36] Japan, 2014 40–55 years Men	Non-drinkers (0.0)	1390	eGFR < 60	Age, BMI, SBP, DBP, FPG, smoking, leisure-time physical activity
	0.1–23.0 g/day (11.5)	3914		
	23.1–46.0 g/day (34.5)	2895		
	46.1–69.0 g/day (57.5)	811		
	≥69.1 g/day (82.9)	102		
PREVEND [37] Netherlands, 2015 Age 28–75 years Men & women	No/rare drinkers (0.0)	1285	UAE > 30 eGFR < 60	Age, sex, height, weight, SBP, hypertension, insulin resistance, diabetes, TCHO/HDL-C, hyperlipidemia, CVD, smoking, education level, parental CKD
	<10 g/week (0.7)	860		
	10–69.9 g/week (5.7)	1949		
	70–210 g/week (20.0)	1121		
	>210 g/week (36.0)	261		
Kansai Healthcare [38] Japan, 2016 Age 40–55 years Men	Non-drinkers (0.0)	1397	Proteinuria ≥ 1+	Age, BMI, hypertension, FPG, eGFR, smoking, leisure-time physical activity
	0.1–23.0 g/day (11.5)	3929		
	23.1–46.0 g/day (34.5)	2909		
	46.1–69.0 g/day (57.5)	816		
	≥69.1 g/day (82.9)	103		
Kimura [20] Japan, 2018 Age 40–75 years Men & women	Rare drinkers (0.0)	57,042	Proteinuria ≥ 1+	Age, BMI, MAP, hypertension, HbA1c, diabetes, HDL-C, dyslipidemia, eGFR, CVD, smoking
	Occasional drinkers (5.0)	57,593		
	≤19 g/d (10.0)	20,818		
	20–39 g/day (30.0)	27,817		
	40–59 g/day (50.0)	11,098		
	≥60 g/day (72.0)	3204		
Park [19] Korea, 2019 Age 20–80 years Men & women	No drinkers (0.0)	7,245,632	Proteinuria ≥ 1+ eGFR < 60	Age, BMI, SBP, hypertension, FPG, diabetes, HDL-C, TG, eGFR, smoking, regular exercise
	<10 g/day (5.0)	3,402,518		
	10–19.9 g/day (15.0)	1,623,400		
	20–39.9 g/day (30.0)	1,361,836		
	≥40 g/day (48.0)	557,492		
ARIC [39] USA, 2020 Age 45–64 years Men & women	Never drinkers (0.0)	3118	eGFR < 60 with eGFR decline > 30%	Age, sex, race-center, BMI, hypertension, diabetes, eGFR, smoking, physical activity, energy intake, education level, income, health insurance
	Former drinkers	2239		
	≤1 drink/week (1.0)	2960		
	2–7 drinks/week (9.0)	2592		
	8–14 drinks/week (22.0)	1029		
	≥15 drinks/week (36.0)	754		
PROMISE [40]	Infrequent drinkers (0.0)	6199	Proteinuria ≥ 1+ eGFR < 60	Age, sex, BMI, hypertension, diabetes, hyperlipidemia, eGFR, smoking
Japan, 2021	<20 g/day (10.0)	3157		
Age 20–74 years	20–39 g/day (30.0)	1162		
Men & women	≥40 g/day (48.0)	657		
Tanaka [21]	No (0.0)	11,369	Proteinuria ≥ 1+ eGFR < 60 with eGFR decline > 25%	Age, BMI, hypertension, diabetes, dyslipidemia, CVD, eGFR, smoking
Japan, 2022	<23 g/day (11.5)	8289		
Age 20–80 years	23–46 g/day (34.5)	5007		
Men & women	≥46 g/day (55.2)	2123		

BMI, body mass index; CKD, chronic kidney disease; CVD, cardiovascular disease; DBP, diastolic blood pressure, eGFR, estimated glomerular filtration rate (mL/min/1.73 m^2^); FPG, fasting plasma glucose; HbA1c, hemoglobin A1c; HDL-C, high density lipoprotein-cholesterol; IGT, impaired glucose tolerance; MAP, mean arterial pressure; RCT, randomized controlled trial; SBP, systolic blood pressure; TCHO, total cholesterol; TG, triglyceride; UAE, urinary albumin excretion (mg/day). * GFR (mL/min) estimated using the Cockcroft-Gault equation. ^†^ Including 35,491 men and 71,298 women with eGFR of ≥60 mL/min/1.73 m^2^ and 5521 men and 11,454 women with eGFR of <60 mL/min/1.73 m^2^. ^‡^ Including 886 men and 653 women with eGFR ≥ 60 mL/min/1.73 m^2^ and 355 men and 507 women with eGFR of <60 mL/min/1.73 m^2^. ^§^ Women were excluded from the present meta-analysis because hazard ratio of women with ethanol >20 g/day was not reported in women.

## 3. Results

The search strategy identified 1457 articles, 1423 of which were excluded after review of the title or abstract (Figure S1). Of the 34 publications selected, the present meta-analysis finally included 12 publications [19,20,21,32,33,34,35,36,37,38,39,40] from 11 cohort studies (*N* = 14,634,940), including the Physicians’ Health Study (PHS) study which defined the outcome as eGFR of <55 mL/min [32]. We excluded 23 publications because two publications were cross-sectional studies [41,42], two did not assess alcohol consumption (g/day) as a predictor of proteinuria and/or low GFR [43,44], three categorized alcohol consumption into only two levels (none vs. ≥1 drink/day [45], no use vs. use of alcohol [46], and alcohol consumption of <20 vs. ≥20 g/day [47]), four did not stratify current drinkers by alcohol consumption level (g/day) [48,49,50,51], one had the highest alcohol consumption category with the lower boundary of <12 g/day, [52] one had a sex-specific definition of alcohol consumption level [53], one did not define the outcome of CKD [54], seven did not have the outcome of incidence of proteinuria or low GFR [55,56,57,58,59,60,61], one had missing information on the number of participants of alcohol consumption categories [62], and one reported similar results in previous publications [63].

The characteristics of the 12 publications from 11 studies are described in Table 1 and Table 2. Of the 11 studies, 6 (7 publications) were conducted in Japan, 2 were from US, and 3 were from Italy, the Netherlands, and Korea (Table 1). The highest alcohol consumption category in each study had a lower boundary of alcohol consumption of 14, 20, 30, 40, 46, 48, 60, and 69.1 g/day, to which we assigned 1.2 times the lower boundary values [27], namely, 16.8, 24.0, 36.0, 48.0, 55.2, 57.6, 72.0, and 82.9 g/day as its representative value. The incidence of proteinuria was defined as a dipstick urinary protein level of ≥1+ or urinary albumin excretion of >30 mg/day that was assessed in 7 studies, and the incidence of low eGFR was defined as <60 mL/min/1.73 m^2^ or < 55 mL/min that was assessed in nine studies. All studies used serum creatinine-based equations to calculate eGFR. The study sample size ranged from 1539 [34] to 14,190,878 [19] (Table 2). The prevalence of diabetes and hypertension was 0.0–13.9% and 0.0–64.9%, respectively and the mean (or median) follow-up period was ≤5, 6–10, and >10 years in 5, 2, and 3 studies, respectively. The study quality was good (NOS ≥ 7) for 5 (45.5%) studies (Table S1). In 6 publications from 5 studies, eGFR was not included as a covariate in the multivariable-adjusted model.

**Figure 1:**
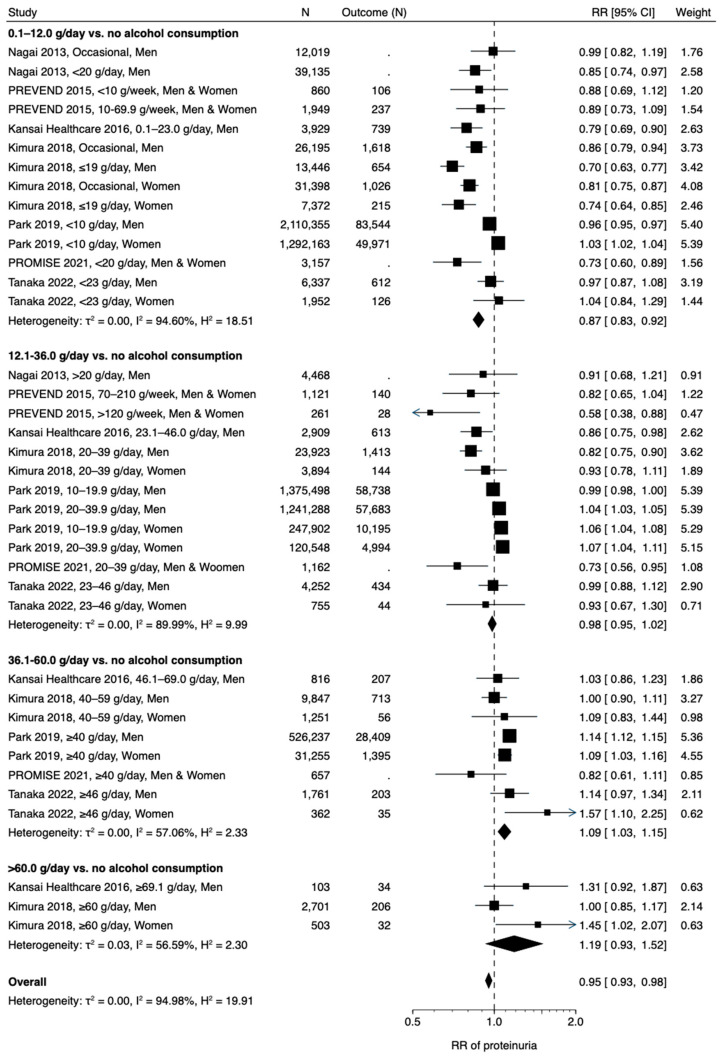
Alcohol consumption and incidence of proteinuria. CI, confidence interval; RR, relative risk [19,20,21,35,37,38,40].

**Figure 2:**
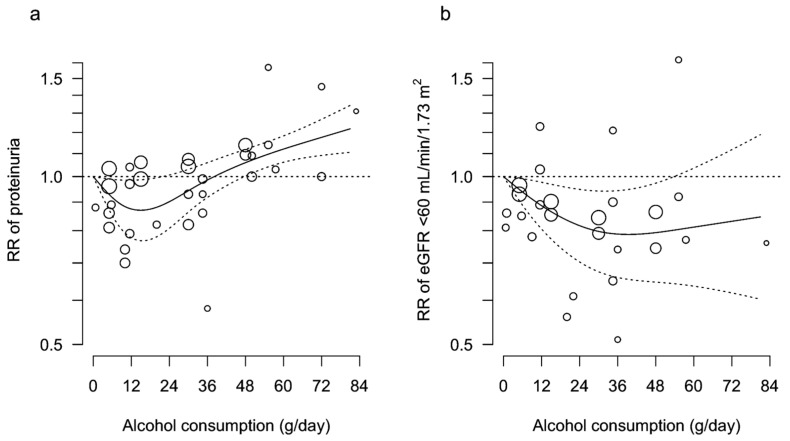
Dose-dependent association between alcohol consumption and incidence of proteinuria (**a**) and low eGFR <60 mL/min/1.73 m^2^ (**b**). RR, relative risk. Solid and dashed curves represent RR and 95% confidence interval, respectively. Circles represent the number of participants of each category of alcohol consumption.

An association between alcohol consumption and incidence of proteinuria stratified by alcohol consumption of 0.1–12.0, 12.1–36.0, 36.1–60.0, and >60.0 g/day is shown in Figure 1. The pooled result of 7 studies [19,20,21,35,37,38,40] that included 14,503,097 participants showed that drinkers were at a significantly lower risk of incidence of proteinuria than non-drinkers (overall RR 0.95 [95% confidence interval 0.93, 0.98]). However, the association was highly dependent on the alcohol consumption levels. Participants with low alcohol consumption of 0.1–12.0 g/day had a significantly lower risk of proteinuria (0.87 [0.83, 0.92]) than non-drinkers, while those with high alcohol consumption of 36.1–60.0 g/day had a significantly higher risk of proteinuria (1.09 [1.03, 1.15]). RR of alcohol consumption of >60.0 g/day (1.19 [0.93, 1.52]) was higher than that of 36.1–60.0 g/day, although a small number of studies with small sample sizes led to an underpowered analysis. A two-stage random-effects dose-response meta-analysis with use of a restricted cubic spline model, which included 14,410,068 participants from 5 studies [19,20,21,37,38], confirmed a J-shaped association between alcohol consumption and incidence of proteinuria (Figure 2a). Funnel plots suggested a potentially biased estimate of pooled RRs in drinkers with alcohol consumption of ≤12.0 g/day and 12.1–36.0 g/day (*p* = 0.041 and <0.001, respectively) (Figure S2a,b and Table S2), but not in those consuming 36.1–60.0 g/day (*p* = 0.141) (Figure S2c and Table S2). Because of substantial heterogeneity in 3 subgroups of alcohol consumption (I^2^ = 94.60%, 89.99%, 57.06%, and 56.59% of ≤12.0, 12.1–36.0, 36.1–60.0, and >60.0 g/day, respectively) (Figure 1 and Table S2), subgroup analyses stratified by sex; the median values of study size, body mass index, eGFR, prevalence of diabetes and hypertension, and follow-up duration; a NOS ≥7; and Asian and Western countries, were employed (Figures S3–S5 and Table S2). In the alcohol consumption subgroup of 36.1–60.0 g/day, age partly contributed to the high heterogeneity (I^2^ = 39.16% and 0.00% in subgroup analyses of age < median and ≥ median, respectively) (Figure S5c).

**Figure 3:**
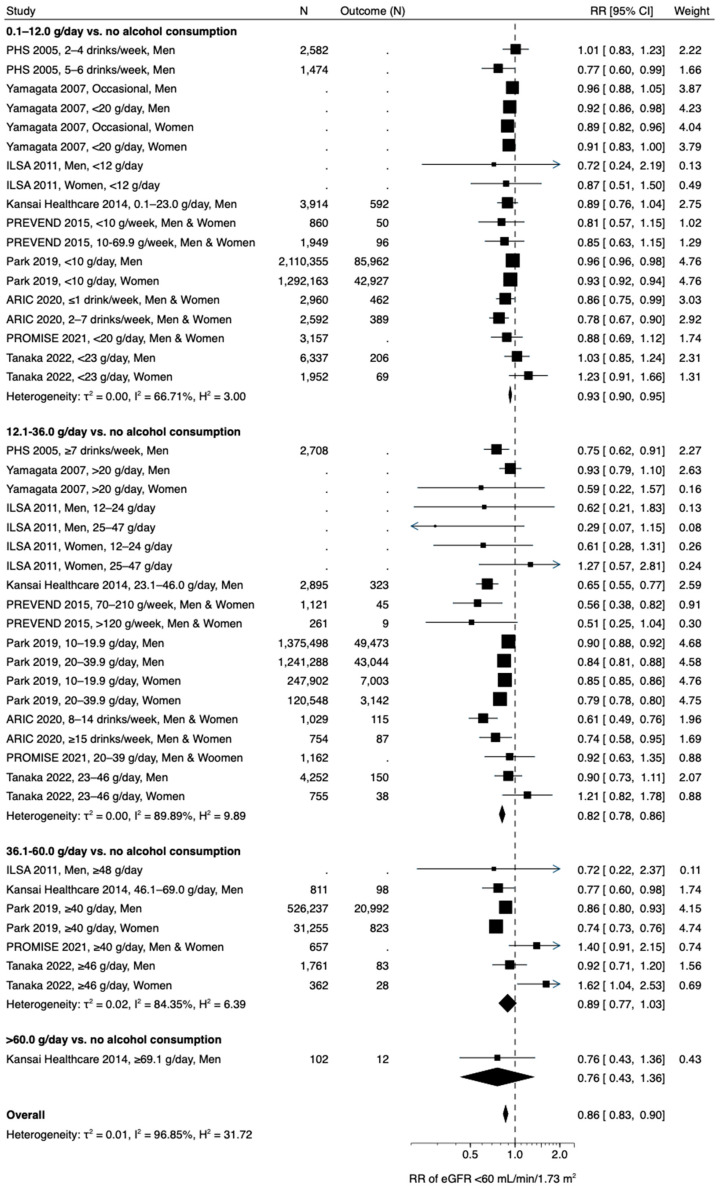
Alcohol consumption and incidence of low eGFR <60 mL/min/1.73 m^2^. CI, confidence interval; eGFR, estimated glomerular filtration rate; RR, relative risk [19,21,32,33,34,36,37,39,40].

An association between alcohol consumption and the incidence of low eGFR was different from that between alcohol consumption and the incidence of proteinuria. A meta-analysis of nine studies, which included 14,375,672 participants [19,21,32,33,34,36,37,39,40], drinkers with alcohol consumption of 0.1–12.0 and 12.1–36.0 g/day were at significantly lower risk for low eGFR than non-drinkers (0.93 [0.90, 0.95] and 0.82 [0.78, 0.86], respectively) (Figure 3). Those with alcohol consumption of 36.1–60.0 g/day were likely to have a lower risk of low eGFR (0.89 [0.77, 1.03]), although not at a statistically significant level. A two-stage random-effects dose-response meta-analysis with use of a restricted cubic spline model, which included 14,245,146 participants from five studies [19,21,36,37,39], showed that a negative linear association between alcohol consumption and incidence of low eGFR was blunted in the range of alcohol consumption of >24 g/day (Figure 2b). Funnel plots suggested publication bias in subgroups of alcohol consumption of 12.1–36.0 and 36.1–60.0 g/day (*p* = 0.048 and 0.020, respectively) (Figure S2e,f and Table S3), although not in a subgroup of ≤12.0 g/day (*p* = 0.167) (Figure S2d and Table S3). Substantial heterogeneity was observed in alcohol consumption of ≤12.0, 12.1–36.0, and 36.1–60.0 g/day (I^2^ = 66.71%, 89.89%, and 84.35%, respectively) (Figure 3 and Table S3). Subgroup analyses (Figures S6–S8 and Table S3) suggested that sex (I^2^ = 0.61% and 17.87% in subgroup analyses of men and women, respectively) (Figure S6a) and body mass index (I^2^ = 0.00% and 0.00% in subgroup analyses of body mass index < median and ≥ median, respectively) (Figure S6d) possibly contributed to this heterogeneity in alcohol consumption of ≤12.0 g/day. Interestingly, four Western cohort studies showed a significantly stronger renoprotective effect of alcohol consumption of ≤12.0 g/day and 12.1–36.0 g/day than five Asian cohort studies (Table S3 and Figures S6j and S7j). However, only a single Western cohort study has assessed an association between alcohol consumption of 36.1–60.0 g/day and the incidence of low eGFR (Table S3).

## 4. Discussion

This systematic review, which included 12 publications from 11 cohort studies, showed that drinkers with low alcohol consumption were at lower risk of proteinuria and low eGFR. Inversely, high alcohol consumption was significantly associated with the incidence of proteinuria (Figure 2a), but not with the incidence of low eGFR (Figure 2b). Several advantages of the present systematic review were, first, assessment of high alcohol consumption of >36.0 g/day, second, separate analyses of a dose-dependent association of alcohol consumption between proteinuria and low eGFR, and third, a selection of publications with clinically relevant well-defined outcomes of proteinuria (dipstick urinary protein of ≥1+ or urinary albumin excretion of >30 mg/day) and low eGFR of <60 mL/min/1.73 m^2^ (or <55 mL/min). This systematic review also disclosed a potential deleterious effect of high alcohol consumption on proteinuria, similar to the findings of the association between high alcohol consumption and cardiometabolic diseases, including hypertension [64], diabetes [65], stroke [66], and heart failure [66].

A previous American systematic review pooled RRs of the highest alcohol consumption category (vs. non-drinkers) in 6 cross-sectional, one case-control, and nine cohort studies, which included a total of 212,918 participants, and showed that the highest alcohol consumption was associated with lower prevalence/incidence of CKD with a wide variety of definitions, including ESKD, eGFR of <60 mL/min/1.73 m^2^, eGFR decline of >3 mL/min/1.73 m^2^, and proteinuria of ≥1+ [67]. This systematic review also reported no significant association between the highest alcohol consumption category and the incidence of proteinuria in four cohort studies with a total of 140,686 participants. However, it was difficult to draw any conclusion on the clinical impact of alcohol consumption on CKD because of the inclusion of many cross-sectional studies and a small number of cohort studies which assessed the association between alcohol consumption and incidence of proteinuria. Two Chinese systematic reviews, which included 268,723 participants from 15 cohort studies [17] and 514,148 participants from 25 prospective cohort studies [18], reported that high alcohol consumption of 26–60 g/day [17] and >24 g/day [18] was associated with a lower incidence of CKD, which was defined variously in each study including ESKD, eGFR <60 mL/min/1.73 m^2^, and eGFR decline of >3 mL/min/1.73 m^2^/year. The present study rigorously defined the outcomes of low eGFR and proteinuria and clarified different dose-dependent associations between alcohol consumption and these outcomes (Figure 2). Inclusion of evidence from large cohort studies [19,20] enabled us to reveal a deleterious effect of high alcohol consumption on proteinuria.

Past drinkers were included as a reference category in nine of 11 cohort studies in this meta-analysis, possibly leading to a biased estimate of the association between alcohol drinking and incidence of proteinuria and low eGFR. Because former drinkers might be inspired to quit drinking due to health concerns, they might be at increased risk of proteinuria and low eGFR, known as the sick-quitter effect. An Australian cohort study clarified the sick-quitter effect among 97,852 drinkers aged ≥45 years [68]. During the median observational period of 5.3 years, 9438 (9.5%) drinkers quit drinking. Among a wide variety of 28 health conditions, including cancers, cardiovascular disease, endocrine conditions, genitourinary conditions, conditions affecting mobility, mental health conditions, and other conditions, 20 health conditions were significantly associated with quitting drinking. The most common health condition in those quitting drinking was heart disease (12.3%), a critical risk factor for incidence of CKD [69] and ESKD [70]. Given that the sick quitters at risk of incidence of CKD were categorized into a reference group, a beneficial effect of alcohol consumption might be overestimated, and its adverse effects might be underestimated. In the present meta-analysis, cohort studies with older age were more likely to show an antiproteinuric effect at ≤36.0 g/day of alcohol consumption and no detrimental effect at 36.1–60.0 g/day of alcohol consumption than those with younger age (Figures S3c, S4c and S5c and Table S2). Sick quitters might contribute to this significant age-dependent association between alcohol consumption and incidence of proteinuria. Evaluation of the association between alcohol consumption and incidences of proteinuria and low eGFR should be assessed more deliberately, considering the sick-quitter effect.

This study had several limitations. First, because seven of 11 (63.6%) cohort studies were reported from Asian countries, including six Japanese cohorts and one Korean cohort, and no Western study has assessed the clinical impact of alcohol consumption of >36.0 g/day on proteinuria and low eGFR (Tables S2 and S3 and Figures S5j and S8j), the results of the present meta-analysis were chiefly based on the genetic background of the Asian population. Alcohol metabolism is greatly dependent on genetic polymorphisms of major alcohol-metabolizing enzymes, alcohol dehydrogenase (ADH) and aldehyde dehydrogenase (ALDH). Asian people are characterized by their unique allele frequencies of major gene polymorphisms of ADH and ALDH, including ADH1B*2 (rs1229984) and ALDH2*2 (rs679). Frequencies of A and C alleles of ADH1B*2 and ALDH2*2 are 74–77% and 11–28%, respectively, in Asian population, whereas their frequencies are almost 0% in other populations [71]. These alleles promote acetaldehyde production and suppress acetaldehyde metabolism, leading to the high acetaldehyde level [71]. These Asian genetic characteristics might contribute to a smaller renoprotective impact of alcohol consumption of ≤12.0 and 12.1–36.0 g/day on the incidence of low eGFR in Asian countries than in Western countries in this study (Table S3 and Figures S6j and S7j). The findings of this meta-analysis, especially the association between alcohol consumption of >36.0 g/day and the incidence of proteinuria and low eGFR, should be verified in non-Asian populations. Second, different definitions of the highest alcohol consumption category among 12 studies might lead to a biased estimate of clinical impacts of alcohol consumption on proteinuria and low eGFR. A large retrospective cohort study, which included 88,647 men and 88,925 women in Japan, carefully showed that the alcohol consumption category affected a dose-dependent association between alcohol consumption and incidence of dipstick proteinuria of ≥1+ [20]. If the alcohol consumption was categorized into four categories of rare drinkers, occasional drinkers, and daily drinkers with ≤19 and ≥20 g/day, alcohol consumption was associated with the incidence of proteinuria in a U-shape fashion in women (multivariable-adjusted hazard ratio [95% CI]: 1.00 [reference], 0.81 [0.75, 0.87], 0.74 [0.64, 0.85], 1.01 [0.88, 1.17], respectively), whereas a J-shaped association was observed after alcohol consumption was categorized into six categories of rare drinkers, occasional drinkers, and daily drinkers with ≤19, 20–39, 40–59, and ≥60 g/day (1.00 [reference], 0.81 [0.75, 0.87], 0.74 [0.64, 0.85], 0.93 [0.78, 1.11], 1.09 [0.84, 1.44], and 1.45 [1.02, 2.08]). Large cohort studies with deliberately categorized alcohol consumption are essential to assess a clinical impact of high alcohol consumption on CKD. Third, binge drinking defined generally as ≥5 and ≥4 standard drinks/occasion in men and women, respectively [72], was not assessed in this meta-analysis. Although binge drinking is a risk factor for cardiovascular diseases [73], little information has been available about an association between binge drinking and incidence of CKD. A prospective cohort study, which included 1883 Korean patients with CKD, reported that patients with occasional binge drinking were at a higher risk of incidence of a 50% increase in eGFR and/or ESKD. Clinical impact of binge drinking on CKD should be clarified in future studies. Fourth, GFR decline might be underestimated in participants with high alcohol consumption in this meta-analysis. Because high alcohol consumption is associated with low muscle mass [74,75] and serum creatinine level is heavily dependent on muscle mass [76], serum creatinine-based eGFR is likely to increase during the observational period among the participants with high alcohol consumption. Thus, a deleterious effect of high alcohol consumption on GFR might be blunted in the cohort studies included in this meta-analysis, in which GFR was estimated using serum creatinine-based equation. The association between high alcohol consumption and GFR trajectory should be assessed using an eGFR equation based on serum cystatin C level, which is independent of muscle mass [77].

## 5. Conclusions

This systematic review, which included 14,634,940 participants from 11 cohort studies, clarified that low alcohol consumption of ≤12 g/day was associated with lower incidence of proteinuria and low eGFR than non-drinkers. However, people with high alcohol consumption of ≥36 g/day were at a higher risk of proteinuria, whereas they were at a lower risk of low eGFR. Clinical impact of high alcohol consumption on the incidence of proteinuria and low eGFR have been assessed chiefly in the Asian population and scarcely in the non-Asian population with genetically different characteristics of alcohol metabolism. The association between high alcohol consumption and CKD should be assessed deliberately in well-designed cohort studies, including a wide variety of ethnic groups.

## Figures and Tables

**Table 2:** Clinical characteristics of 12 publications of 11 cohort studies stratified by sex subgroups.

Study Subgroup	*N*	Men (%)	Age (year)	BMI (kg/m^2^)	eGFR (mL/min/1.73 m^2^)	DM (%)	HT (%)	Follow-Up (Year)	NOS
PHS 2005, men [32]	11,023	100.0	52.9	24.9	NA	2.0	20.9 ^||^	14.2	5
Yamagata 2007, men [33]	35,491	100.0	61.8 ± 10.2 *	23.2 ± 2.9 *	81.9 ± 14.5 *	3.6 *^‡^	21.0 *^‡^	NA	6
Yamagata 2007, women [33]	71,298	0.0	58.3 ± 10.0 *	23.5 ± 3.2 *	79.8 ± 14.2 *	2.1 *^‡^	18.9 *^‡^	NA	6
ILSA 2011, men [34]	886	100.0	71.9 ^†^	26.5 ^†^	NA	13.5 ^†^	64.9 ^†^	3.5	6
ILSA 2011, women [34]	653	0.0	73.1 ^†^	27.6 ^†^	NA	13.9 ^†^	73.2 ^†^	3.5	6
Nagai 2013, men [35]	81,854	100.0	60.2 ± 9.7	23.4 ± 2.9	NA	7.5 ^||^	52.4 ^||^	4.0	7
Kansai Healthcare 2014 [36]	9112	100.0	48.2 ± 4.2	23.2 ± 2.8	84.7 ± 14.0	0.0 ^‡^	0.0 ^‡^	8.7	5
PREVEND 2015 [37]	5476	47.4	48.4 ± 11.7	25.7 ± 4.0	97.3 ± 14.8	1.0 ^‡^	11.7 ^‡^	10.2 (6.2–11.4)	7
Kansai Healthcare 2016 [38]	9154	100.0	48.2 ± 4.2	23.2 ± 2.8	84.7 ± 14.0	0.0 ^‡^	0.0 ^‡^	8.0	5
Kimura 2018, men [20]	88,647	100.0	65 (58–69)	23.6 ± 3.0	75 (69–86)	5.7 ^§^	28.8 ^§^	1.8 (1.0–2.2)	7
Kimura 2018, women [20]	88,925	0.0	65 (59–69)	22.6 ± 3.3	76 (68–90)	3.0 ^§^	24.1 ^§^	1.7 (1.0–2.1)	7
Park 2019, men [19]	7,625,277	100.0	44.7	NA	91.7	3.9 ^‡^	10.7 ^‡^	6.4	8
Park 2019, women [19]	6,565,601	0.0	47.9	NA	92.6	3.6 ^‡^	13.3 ^‡^	6.4	8
ARIC 2020 [39]	12,692	55.9	54 ± 6	27.4	103.3	10.1 ^¶^	25.1 ^‡^	24 **	8
PROMISE 2021 [40]	11,175	40.2	62 (55–67)	22.3 ± 3.1	78 ± 12	3.4 ^‡^	17.8 ^‡^	5.0 (2.9–7.6)	6
Tanaka 2022, men [21]	19,702	100.0	42 ^††^	23.4 ^††^	86 ^††^	3.1 ^§^	9.4 ^§^	4 (3–6)	5
Tanaka 2022, women [21]	7086	0.0	43 ^††^	21.4 ^††^	76 ^††^	1.3 ^§^	5.7 ^§^	4 (2–5)	5

Mean ± standard deviation, Median (25%–75%). BMI, body mass index; DM, diabetes; eGFR, estimated glomerular filtration rate; HT, hypertension; NA, not available; NOS, Newcastle-Ottawa Scale. * Including 35,491 men and 71,298 women with eGFR of ≥60 mL/min/1.73 m^2^ and 5521 men and 11,454 women with eGFR of <60 mL/min/1.73 m^2^. ^†^ Including 886 men and 653 women with eGFR ≥60 mL/min/1.73 m^2^ and 355 men and 507 women with eGFR of <60 mL/min/1.73 m^2^. ^‡^ Use of anti-diabetic or anti-hypertensive drugs. ^§^ Current treatment for hypertension or diabetes. ^||^ Diabetes defined as fasting plasma glucose of ≥126 mg/dL, random plasma glucose ≥200 mg/dL, and/or use of anti-diabetic drugs; and hypertension defined as systolic blood pressure ≥140 mmHg, diastolic blood pressure ≥90 mmHg, and/or use of anti-hypertensive drugs. ^¶^ Diabetes defined as fasting plasma glucose of ≥126 mg/dL, random plasma glucose ≥200 mg/dL, use of anti-diabetic drugs, and/or self-reported diabetes. ** Median value. ^††^ A mean value of all men or women in a study was calculated after the mean value of each alcohol consumption group was estimated using the equation: mean = (Q1 + Q3 + median) ÷ 3, where Q1 and Q3 are the first and third quartiles, respectively [26].

## Data Availability

Not applicable.

## Supplementary Materials

The following supporting information can be downloaded at: https://www.mdpi.com/article/10.3390/nu15071592/s1, S1: Newcastle-Ottawa scale (NOS) of 12 publications from 11 cohort studies; Table S2: Alcohol consumption and incidence of proteinuria stratified by major study characteristics; Table S3: Alcohol consumption and incidence of eGFR of <60 mL/min/1.73 m^2^ stratified by major study characteristics; Figure legends; Figure S1: Flow diagram of study selection; Figure S2: Funnel plots of relative risks to estimate associations of alcohol consumption of ≤12.0 (a), 12.1–36.0 (b), and 36.1–60.0 (c) with incidence of proteinuria and those of ≤12.0 (d), 12.1–36.0 (e), and 36.1–60.0 (f) with incidence of eGFR <60 mL/min/1.73 m^2^; Figure S3: Forest plots of relative risks to estimate an association between alcohol consumption of <12 g/day and incidence of proteinuria stratified by sex (a), study size (b), age (c), body mass index (d), eGFR (e), prevalence of diabetes (f), prevalence of hypertension (g), follow-up length (h), and Newcastle-Ottawa scale (i); Figure S4: Forest plots of relative risks to estimate an association between alcohol consumption of 12.1–36.0 g/day and incidence of proteinuria stratified by sex (a), study size (b), age (c), body mass index (d), eGFR (e), prevalence of diabetes (f), prevalence of hypertension (g), follow-up length (h), Newcastle-Ottawa scale (i), and Asian and Western countries (j); Figure S5: Forest plots of relative risks to estimate an association between alcohol consumption of 36.0–60.0 g/day and incidence of proteinuria stratified by sex (a), study size (b), age (c), body mass index (d), eGFR (e), prevalence of diabetes (f), prevalence of hypertension (g), follow-up length (h), Newcastle-Ottawa scale (i), and Asian and Western countries (j); Figure S6: Forest plots of relative risks to estimate an association between alcohol consumption of <12 g/day and incidence of eGFR <60 mL/min/1.73 m^2^ stratified by sex (a), study size (b), age (c), body mass index (d), eGFR (e), prevalence of diabetes (f), prevalence of hypertension (g), follow-up length (h), Newcastle-Ottawa scale (i), and Asian and Western countries (j); Figure S7: Forest plots of relative risks to estimate an association between alcohol consumption of 12.1–36.0 g/day and incidence of eGFR <60 mL/min/1.73 m^2^ stratified by sex (a), study size (b), age (c), body mass index (d), eGFR (e), prevalence of diabetes (f), prevalence of hypertension (g), follow-up length (h), Newcastle-Ottawa scale (i), and Asian and Western countries (j); Figure S8: Forest plots of relative risks to estimate an association between alcohol consumption of 36.0–60.0 g/day and incidence of eGFR <60 mL/min/1.73 m^2^ stratified by sex (a), study size (b), age (c), body mass index (d), eGFR (e), prevalence of diabetes (f), prevalence of hypertension (g), follow-up length (h), Newcastle-Ottawa scale (i), and Asian and Western countries (j).

## Appendix A. Search Strategies in PubMed and Web of Science

### Appendix A.1. Pubmed

#1 glomerular filtration rate OR proteinuria
#2 alcohol
#3 “2000/1/1” [Date - Publication]: “2022/12/31” [Date - Publication]
#4 “English” [Language]
#5 #1 AND #2 AND #3 AND #4
#6 systematic review[Title] OR meta-analysis[Title] OR guidelines[Title] OR recommendations[Title] OR cross-sectional[Title] OR case report[Title] OR case reports[Title] OR case series[Title]
#7 rat[Title] OR rats[Title] OR rodent[Title] OR mouse[Title] OR mice[Title] OR murine[Title] OR dog[Title] OR dogs[Title] OR porcine[Title] OR rabbit[Title] OR rabbits[Title] OR zebrafish[Title] OR in vivo[Title] OR in vitro[Title]
#8 #5 NOT #6 NOT #7

Search results: 1273

### Appendix A.2. Web of Science

#1 ALL=(glomerular filtration rate) OR ALL=(proteinuria)
#2 #1 AND ALL=(alcohol)
#3 #2 AND PY=(2000-2022)
#4 #3 AND LA=(English)
#5 #4 NOT TI=(systematic review) NOT TI=(meta-analysis) NOT TI=(guidelines) NOT TI=(cross-sectional) NOT TI=(case report) NOT TI=(case reports) NOT TI=(case series)
#6 #5 NOT TI=(rat) NOT TI=(rats) NOT TI=(rodent) NOT TI=(mouse) NOT TI=(mice) NOT TI=(murine) NOT TI=(dog) NOT TI=(dogs) NOT TI=(porcine) NOT TI=(rabbit) NOT TI=(rabbits) NOT TI=(zebrafish) NOT TI=(in vivo) NOT TI=(in vitro)

Search results: 559
