# Supplementary material for: A Dose-Dependent Association between Alcohol Consumption and Incidence of Proteinuria and Low Glomerular Filtration Rate: A Systematic Review and Meta-Analysis of Cohort Studies
Source: Nutrients. 2023 Mar 25;15(7):1592. doi: 10.3390/nu15071592 (PMC10097279; doi:10.3390/nu15071592)
Supplement: Supplementary file 1 [file nutrients-15-01592-s001.zip › nutrients-2259660-supplementary.pdf]

Supplementary Materials

Table S1. Newcastle–Ottawa scale (NOS) of 12 publications from 11 cohort studies.
Table S2. Alcohol consumption and incidence of proteinuria stratified by major study characteristics.
Table S3. Alcohol consumption and incidence of eGFR of <60 mL/min/1.73 m^2 stratified by major study characteristics.
Figure legends.
Figure S1. Flow diagram of study selection.
Figure S2. Funnel plots of relative risks to estimate associations of alcohol consumption of ≤12.0 (a), 12.1–36.0 (b), and 36.1–60.0 (c) with incidence of proteinuria and those of ≤12.0 (d), 12.1–36.0 (e), and 36.1–60.0 (f) with incidence of eGFR <60 mL/min/1.73 m^2.
Figure S3. Forest plots of relative risks to estimate an association between alcohol consumption of <12 g/day and incidence of proteinuria stratified by sex (a), study size (b), age (c), body mass index (d), eGFR (e), prevalence of diabetes (f), prevalence of hypertension (g), follow-up length (h), Newcastle-Ottawa scale (i), and Asian and Western countries (j).
Figure S4. Forest plots of relative risks to estimate an association between alcohol consumption of 12.1–36.0 g/day and incidence of proteinuria stratified by sex (a), study size (b), age (c), body mass index (d), eGFR (e), prevalence of diabetes (f), prevalence of hypertension (g), follow-up length (h), Newcastle-Ottawa scale (i), and Asian and Western countries (j).
Figure S5 Forest plots of relative risks to estimate an association between alcohol consumption of 36.0–60.0 g/day and incidence of proteinuria stratified by sex (a), study size (b), age (c), body mass index (d), eGFR (e), prevalence of diabetes (f), prevalence of hypertension (g), follow-up length (h), Newcastle-Ottawa scale (i), and Asian and Western countries (j).
Figure S6. Forest plots of relative risks to estimate an association between alcohol consumption of <12 g/day and incidence of eGFR <60 mL/min/1.73 m^2 stratified by sex (a), study size (b), age (c), body mass index (d), eGFR (e), prevalence of diabetes (f), prevalence of hypertension (g), follow-up length (h), Newcastle-Ottawa scale (i), and Asian and Western countries (j).
Figure S7. Forest plots of relative risks to estimate an association between alcohol consumption of 12.1–36.0 g/day and incidence of eGFR <60 mL/min/1.73 m^2 stratified by sex (a), study size (b), age (c), body mass index (d), eGFR (e), prevalence of diabetes (f), prevalence of hypertension (g), follow-up length (h), Newcastle-Ottawa scale (i), and Asian and Western countries (j).
Figure S8. Forest plots of relative risks to estimate an association between alcohol consumption of 36.0–60.0 g/day and incidence of eGFR <60 mL/min/1.73 m^2 stratified by sex (a), study size (b), age (c), body mass index (d), eGFR (e), prevalence of diabetes (f), prevalence of hypertension (g), follow-up length (h), Newcastle-Ottawa scale (i), and Asian and Western countries (j).

Table S1. Newcastle–Ottawa scale of 12 publications from 11 cohort studies.

Publications	PHS 2005	Yamagata 2007	ILSA 2011	Nagai 2013	Knasai Healthcare 2014	PREVEND 2015	Knasai Healthcare 2018	Kimura 2018	Park 2019	ARIC 2020	PROMISE 2021	Tanaka 2022
Selection												
Representativeness		*	*	*		*		*	*	*		
Selection		*	*	*		*		*	*	*		
Ascertainment												
Outcome	*	*	*	*	*	*	*	*	*	*	*	*
Comparability	*	*	*	**		*	*	**	**	**	**	**
Outcome												
Assessment	*	*	*	*	*	*	*	*	*	*	*	*
Follow-up	*				*	*	*		*	*	*	
Adequacy	*	*	*	*	*	*	*	*	*	*	*	*
Total score	5	6	6	7	5	7	5	7	8	8	6	5

Newcastle-Ottawa scale includes 8 items of 3 domains: (i) Selection, including representativeness of the exposed cohort, selection of the non-exposed, and ascertainment of exposure, demonstration that outcome of interest was not present at start of study; (ii) comparability of cohorts on the basis of the design or analysis; and (iii) outcome, including assessment of outcome, follow-up length enough for outcome to occur, and adequacy of follow-up of cohorts.

Table S2. Alcohol consumption and incidence of proteinuria stratified by study characteristics.

		≤12.0 g/day			12.1–36.0 g/day			36.1–60.0 g/day		
		N*	RR (95% CI)	I^2	N*	RR (95% CI)	I^2	N*	RR (95% CI)	I^2
Sex, N‡	Men	7 (5)	0.87 (0.79, 0.96)	89	6 (5)	0.96 (0.92, 1.01)	93	4 (4)	1.09 (1.01, 1.18)	57
	Women	4 (3)	0.89 (0.74, 1.07)	95	4 (3)	1.06 (1.04, 1.08)	10	3 (3)	1.16 (0.98, 1.37)	47
Study size, N‡	< Median	6 (4)	0.88 (0.79, 0.98)	55	6 (4)	0.85 (0.73, 0.96)	50	4 (4)	1.09 (0.90, 1.32)	63
	≥ Median	8 (3)	0.87 (0.82, 0.92)	97	7 (3)	1.01 (0.97, 1.05)	94	4 (2)	1.10 (1.04, 1.16)	60
Age, yr†‡§	< Median	7 (4)	0.96 (0.91, 1.01)	95	6 (2)	1.04 (1.00, 1.07)	93	4 (2)	1.13 (1.08, 1.18)	39
	≥ Median	7 (3)	0.80 (0.74, 0.87)	66	7 (5)	0.83 (0.78, 0.89)	1	4 (3)	1.00 (0.92, 1.08)	0
Body mass index, kg/m^2	< Median	5 (4)	0.80 (0.74, 0.88)	49	4 (4)	0.87 (0.79, 0.95)	0	3 (3)	1.11 (0.78, 1.56)	73
	≥ Median	7 (4)	0.87 (0.78, 0.96)	74	5 (4)	0.86 (0.75, 0.97)	60	3 (3)	1.04 (0.96, 1.12)	0
eGFR, mL/min/1.73 m^2†‡	< Median	6 (3)	0.80 (0.73, 0.87)	71	6 (4)	0.88 (0.81, 0.95)	41	4 (3)	1.06 (0.87, 1.29)	62
	≥ Median	6 (4)	0.95 (0.90, 1.01)	96	6 (2)	1.03 (0.99, 1.07)	94	4 (3)	1.14 (1.12, 1.15)	0
DM prevalence, %‡	< Median	7 (4)	0.86 (0.79, 0.94)	62	7 (5)	0.87 (0.79, 0.96)	41	4 (3)	1.14 (0.99, 1.30)	30
	≥ Median	7 (4)	0.89 (0.84, 0.95)	97	6 (3)	1.01 (0.97, 1.05)	95	4 (3)	1.08 (1.00, 1.15)	75
HT prevalence, %†	< Median	7 (4)	0.96 (0.91, 1.01)	95	7 (4)	0.98 (0.94, 1.03)	90	4 (3)	1.14 (1.06, 1.23)	30
	≥ Median	7 (3)	0.80 (0.74, 0.87)	66	6 (4)	0.96 (0.89, 1.04)	88	4 (3)	1.04 (0.95, 1.13)	42
Follow-up duration, yr‡	< Median	8 (3)	0.85 (0.78, 0.93)	77	6 (4)	0.88 (0.81, 0.97)	41	4 (2)	1.12 (0.97, 1.29)	54
	≥ Median	6 (4)	0.93 (0.87, 0.99)	96	7 (3)	1.02 (0.98, 1.06)	93	4 (3)	1.10 (1.04, 1.17)	60
Newcastle-Ottawa score	< 7	4 (3)	0.87 (0.75, 1.02)	73	4 (3)	0.89 (0.79, 1.01)	44	4 (3)	1.09 (0.90, 1.32)	63
	≥ 7	10 (4)	0.87 (0.83, 0.92)	96	9 (4)	1.00 (0.96, 1.04)	92	4 (2)	1.10 (1.04, 1.16)	60
Country, N	Asian	13 (6)	0.87 (0.83, 0.92)	95	11 (6)	0.99 (0.96, 1.03)	91	7 (5)	1.09 (1.03, 1.15)	57
	Western	2 (1)			2 (1)			0 (0)		
All		14 (7)	0.87 (0.83, 0.92)	95	13 (7)	0.98 (0.95, 1.02)	90	8 (5)	1.09 (1.03, 1.15)	57
P for Egger's test			0.041			<0.001			0.141	

CI, confidence interval; RR, relative risk

*Number of estimates (Number of publications)

†P <0.05 for group difference in alcohol consumption of ≤12.0 g/day

‡P <0.05 for group difference in alcohol consumption of 12.1–36.0 g/day

§P <0.05 for group difference in alcohol consumption of 36.1–60.0 g/day

Table S3. Alcohol consumption and incidence of low eGFR of <60 mL/min/1.73 m^2 stratified by study characteristics.

		≤12.0 g/day			12.1–36.0 g/day			36.1–60.0 g/day		
		N*	RR (95% CI)	I^2	N*	RR (95% CI)	I^2	N*	RR (95% CI)	I^2
Sex, N†	Men	8 (6)	0.96 (0.95, 0.97)	1	8 (6)	0.84 (0.78, 0.90)	74	4 (4)	0.86 (0.80, 0.92)	0
	Women	5 (4)	0.92 (0.89, 0.96)	18	6 (4)	0.83 (0.77, 0.90)	95	2 (2)		
Study size, N	< Median	9 (6)	0.91 (0.83, 0.99)	0	9 (5)	0.72 (0.59, 0.87)	47	3 (3)	1.01 (0.56, 1.83)	76
	≥ Median	9 (4)	0.93 (0.90, 0.96)	81	10 (5)	0.84 (0.80, 0.88)	94	4 (3)	0.86 (0.74, 1.00)	89
Age, yr‡	< Median	9 (5)	0.95 (0.91, 0.98)	79	9 (4)	0.83 (0.79, 0.88)	95	3 (2)	1.01 (0.77, 1.32)	74
	≥ Median	9 (4)	0.90 (0.87, 0.94)	0	10 (5)	0.76 (0.66, 0.88)	38	4 (4)	0.84 (0.67, 1.07)	65
Body mass index, kg/m^2†‡	< Median	8 (4)	0.92 (0.89, 0.96)	0	7 (5)	0.84 (0.72, 0.98)	62	2 (2)		
	≥ Median	8 (4)	0.84 (0.78, 0.91)	0	8 (3)	0.65 (0.56, 0.74)	0	3 (3)	0.83 (0.70, 0.99)	0
eGFR, mL/min/1.73 m^2‡	< Median	7 (4)	0.92 (0.89, 0.96)	0	8 (5)	0.86 (0.80, 0.92)	72	3 (3)	1.17 (0.71, 1.94)	82
	≥ Median	7 (4)	0.93 (0.90, 0.97)	85	6 (3)	0.78 (0.73, 0.84)	96	3 (2)	0.82 (0.71, 0.94)	89
DM prevalence, %‡	< Median	9 (5)	0.92 (0.86, 0.97)	15	11 (7)	0.81 (0.76, 0.86)	92	3 (2)	1.00 (0.70, 1.43)	76
	≥ Median	9 (5)	0.93 (0.90, 0.96)	80	8 (3)	0.82 (0.76, 0.89)	73	4 (3)	0.85 (0.72, 0.99)	88
HT prevalence, %	< Median	10 (6)	0.94 (0.91, 0.97)	77	10 (5)	0.83 (0.79, 0.88)	94	4 (3)	0.92 (0.76, 1.12)	76
	≥ Median	8 (4)	0.90 (0.85, 0.95)	26	9 (4)	0.75 (0.64, 0.88)	42	3 (3)	0.93 (0.56, 1.55)	76
Follow-up duration, yr†	< Median	7 (4)	0.95 (0.92, 0.98)	83	7 (3)	0.92 (0.76, 1.12)	13	3 (2)	1.11 (0.70, 1.75)	60
	≥ Median	7 (4)	0.85 (0.80, 0.92)	0	10 (5)	0.80 (0.76, 0.85)	95	4 (3)	0.83 (0.72, 0.96)	88
Newcastle-Ottawa score	< 7	12 (6)	0.92 (0.89, 0.96)	0	11 (6)	0.83 (0.71, 0.96)	50	5 (4)	1.05 (0.78, 1.42)	66
	≥ 7	6 (3)	0.93 (0.90, 0.96)	87	8 (3)	0.82 (0.78, 0.87)	96	2 (1)		
Country, N†‡	Asian	10 (5)	0.94 (0.91, 0.96)	77	10 (5)	0.84 (0.80, 0.89)	94	6 (4)	0.90 (0.78, 1.03)	87
	Western	8 (4)	0.84 (0.78, 0.91)	0	9 (4)	0.68 (0.61, 0.76)	0	1 (1)		
All		18 (9)	0.93 (0.90, 0.95)	67	19 (9)	0.82 (0.78, 0.86)	90	7 (5)	0.89 (0.77, 1.03)	84
P for Egger's test			0.167			0.048			0.020	

CI, confidence interval; RR, relative risk

*Number of estimates (Number of publications)

†P <0.05 for group difference in alcohol consumption of ≤12.0 g/day

‡P <0.05 for group difference in alcohol consumption of 12.1–36.0 g/day

Figure Legends

Figure S1. Flow diagram of study selection.

Figure S2. Forest plots of relative risks to estimate associations of alcohol consumption of ≤12.0 (a), 12.1–36.0 (b), and 36.1–60.0 (c) with incidence of proteinuria and those of ≤12.0 (d), 12.1–36.0 (e), and 36.1–60.0 (f) with incidence of eGFR <60 mL/min/1.73 m^2.

Figure S3. Forest plots of relative risks to estimate an association between alcohol consumption of <12 g/day and incidence of proteinuria stratified by sex (a), study size (b), age (c), body mass index (d), eGFR (e), prevalence of diabetes (f), prevalence of hypertension (g), follow-up length (h), Newcastle-Ottawa scale (i), and Asian and Western countries (j).
CI, confidence interval; DM, diabetes mellitus; eGFR, estimated glomerular filtration rate; HT, hypertension; NOS, Newcastle-Ottawa scale; RR, relative risk.

Figure S4. Forest plots of relative risks to estimate an association between alcohol consumption of 12.1–36.0 g/day and incidence of proteinuria stratified by sex (a), study size (b), age (c), body mass index (d), eGFR (e), prevalence of diabetes (f), prevalence of hypertension (g), follow-up length (h), Newcastle-Ottawa scale (i), and Asian and Western countries (j).
CI, confidence interval; DM, diabetes mellitus; eGFR, estimated glomerular filtration rate; HT, hypertension; NOS, Newcastle-Ottawa scale; RR, relative risk.

Figure S5 Forest plots of relative risks to estimate an association between alcohol consumption of 36.0–60.0 g/day and incidence of proteinuria stratified by sex (a), study size (b), age (c), body mass index (d), eGFR (e), prevalence of diabetes (f), prevalence of hypertension (g), follow-up length (h), Newcastle-Ottawa scale (i), and Asian and Western countries (j).
CI, confidence interval; DM, diabetes mellitus; eGFR, estimated glomerular filtration rate; HT, hypertension; NOS, Newcastle-Ottawa scale; RR, relative risk.

Figure S6. Forest plots of relative risks to estimate an association between alcohol consumption of <12 g/day and incidence of eGFR <60 mL/min/1.73 m^2 stratified by sex (a), study size (b), age (c), body mass index (d), eGFR (e), prevalence of diabetes (f), prevalence of hypertension (g), follow-up length (h), Newcastle-Ottawa scale (i), and Asian and Western countries (j).
CI, confidence interval; DM, diabetes mellitus; eGFR, estimated glomerular filtration rate; HT, hypertension; NOS, Newcastle-Ottawa scale; RR, relative risk.

Figure S7. Forest plots of relative risks to estimate an association between alcohol consumption of 12.1–36.0 g/day and incidence of eGFR <60 mL/min/1.73 m^2 stratified by sex (a), study size (b), age (c), body mass index (d), eGFR (e), prevalence of diabetes (f),

prevalence of hypertension (g), follow-up length (h), Newcastle-Ottawa scale (i), and Asian and Western countries (j).
CI, confidence interval; DM, diabetes mellitus; eGFR, estimated glomerular filtration rate; HT, hypertension; NOS, Newcastle-Ottawa scale; RR, relative risk.

Figure S8. Forest plots of relative risks to estimate an association between alcohol consumption of 36.0–60.0 g/day and incidence of eGFR <60 mL/min/1.73 m^2 stratified by sex (a), study size (b), age (c), body mass index (d), eGFR (e), prevalence of diabetes (f), prevalence of hypertension (g), follow-up length (h), Newcastle-Ottawa scale (i), and Asian and Western countries (j).
CI, confidence interval; DM, diabetes mellitus; eGFR, estimated glomerular filtration rate; HT, hypertension; NOS, Newcastle-Ottawa scale; RR, relative risk.

Figure S1

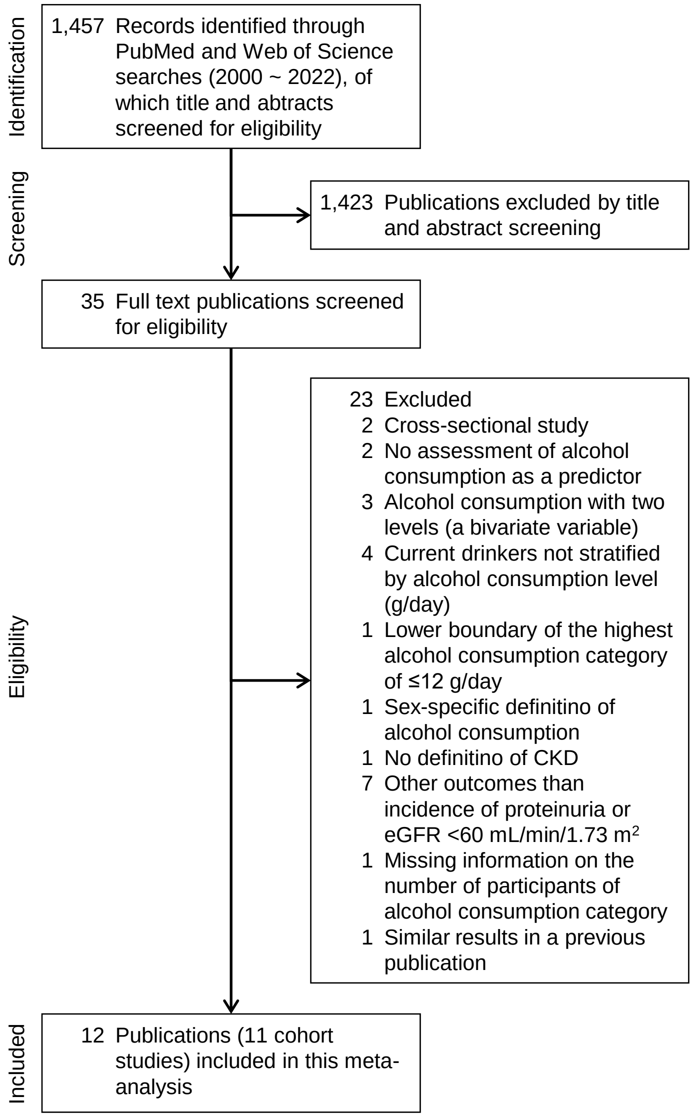
Identification
1,457 Records identified through PubMed and Web of Science searches (2000 ~ 2022), of which title and abtracts screened for eligibility
Screening
1,423 Publications excluded by title and abstract screening
35 Full text publications screened for eligibility
Eligibility
23 Excluded
2 Cross-sectional study
2 No assessment of alcohol consumption as a predictor
3 Alcohol consumption with two levels (a bivariate variable)
4 Current drinkers not stratified by alcohol consumption level (g/day)
1 Lower boundary of the highest alcohol consumption category of ≤12 g/day
1 Sex-specific definitino of alcohol consumption
1 No definitino of CKD
7 Other outcomes than incidence of proteinuria or eGFR <60 mL/min/1.73 m2
1 Missing information on the number of participants of alcohol consumption category
1 Similar results in a previous publication
Included
12 Publications (11 cohort studies) included in this meta-analysis

Figure S2

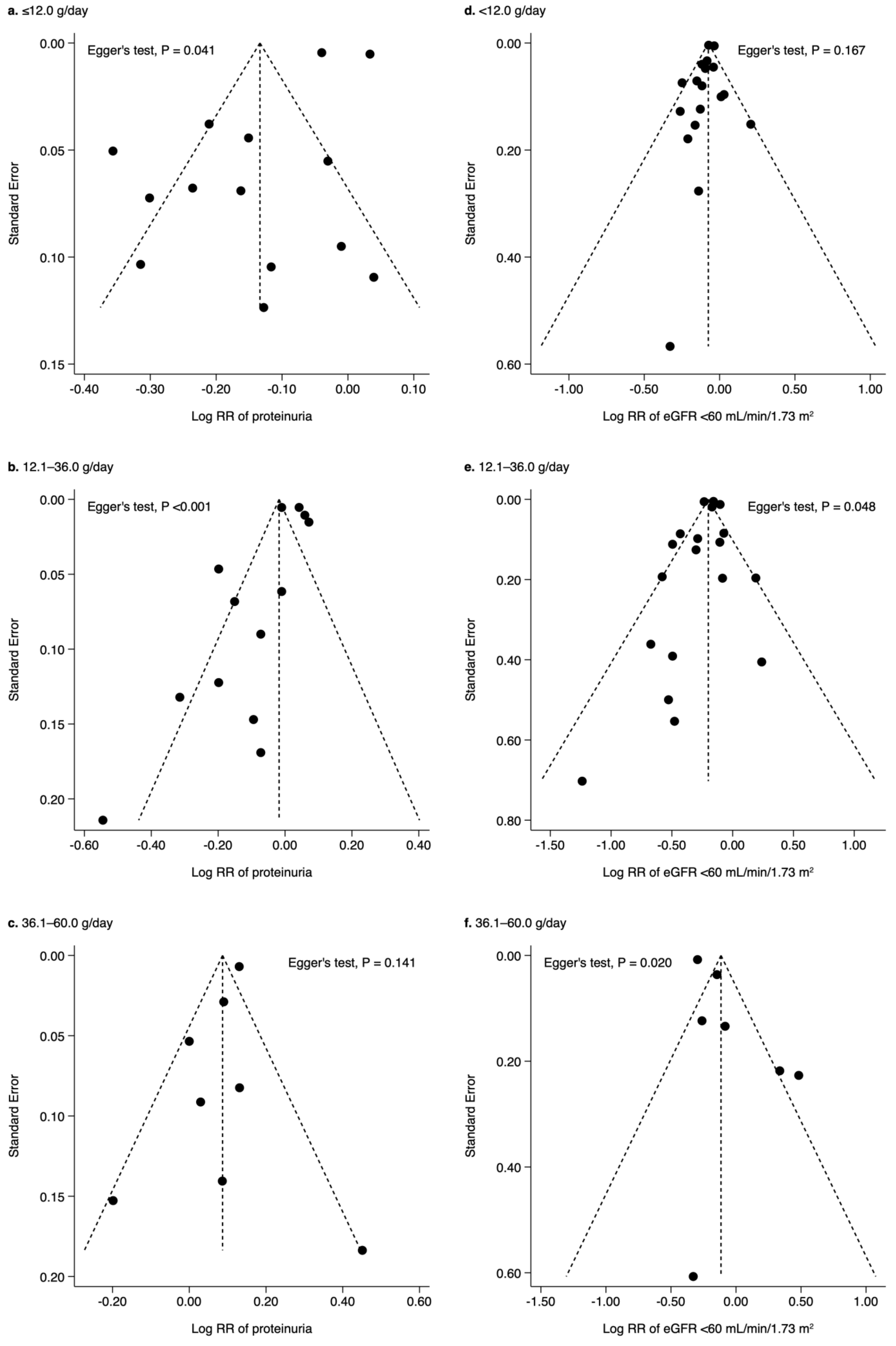
a. ≤12.0 g/day
Egger's test, P = 0.041
Standard Error
Log RR of proteinuria
d. <12.0 g/day
Egger's test, P = 0.167
Standard Error
Log RR of eGFR <60 mL/min/1.73 m²
b. 12.1–36.0 g/day
Egger's test, P <0.001
Standard Error
Log RR of proteinuria
e. 12.1–36.0 g/day
Egger's test, P = 0.048
Standard Error
Log RR of eGFR <60 mL/min/1.73 m²
c. 36.1–60.0 g/day
Egger's test, P = 0.141
Standard Error
Log RR of proteinuria
f. 36.1–60.0 g/day
Egger's test, P = 0.020
Standard Error
Log RR of eGFR <60 mL/min/1.73 m²

Figure S3a

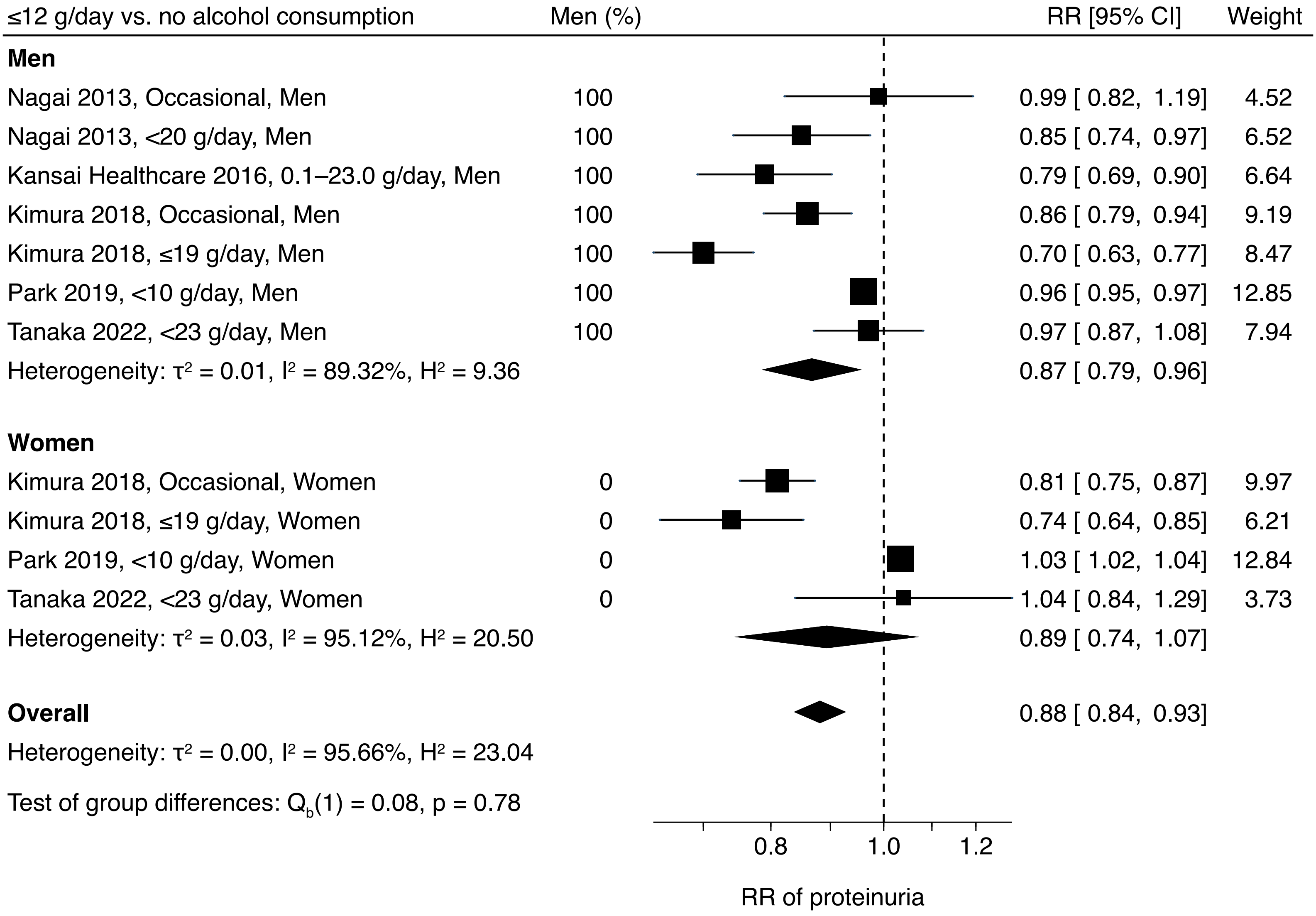
≤12 g/day vs. no alcohol consumption
Men (%)
RR [95% CI]
Weight
Men
Nagai 2013, Occasional, Men 100 0.99 [0.82, 1.19] 4.52
Nagai 2013, <20 g/day, Men 100 0.85 [0.74, 0.97] 6.52
Kansai Healthcare 2016, 0.1–23.0 g/day, Men 100 0.79 [0.69, 0.90] 6.64
Kimura 2018, Occasional, Men 100 0.86 [0.79, 0.94] 9.19
Kimura 2018, ≤19 g/day, Men 100 0.70 [0.63, 0.77] 8.47
Park 2019, <10 g/day, Men 100 0.96 [0.95, 0.97] 12.85
Tanaka 2022, <23 g/day, Men 100 0.97 [0.87, 1.08] 7.94
Heterogeneity: τ² = 0.01, I² = 89.32%, H² = 9.36 0.87 [0.79, 0.96]
Women
Kimura 2018, Occasional, Women 0 0.81 [0.75, 0.87] 9.97
Kimura 2018, ≤19 g/day, Women 0 0.74 [0.64, 0.85] 6.21
Park 2019, <10 g/day, Women 0 1.03 [1.02, 1.04] 12.84
Tanaka 2022, <23 g/day, Women 0 1.04 [0.84, 1.29] 3.73
Heterogeneity: τ² = 0.03, I² = 95.12%, H² = 20.50 0.89 [0.74, 1.07]
Overall 0.88 [0.84, 0.93]
Heterogeneity: τ² = 0.00, I² = 95.66%, H² = 23.04
Test of group differences: Q_b(1) = 0.08, p = 0.78
0.8
1.0
1.2
RR of proteinuria

Figure S3b

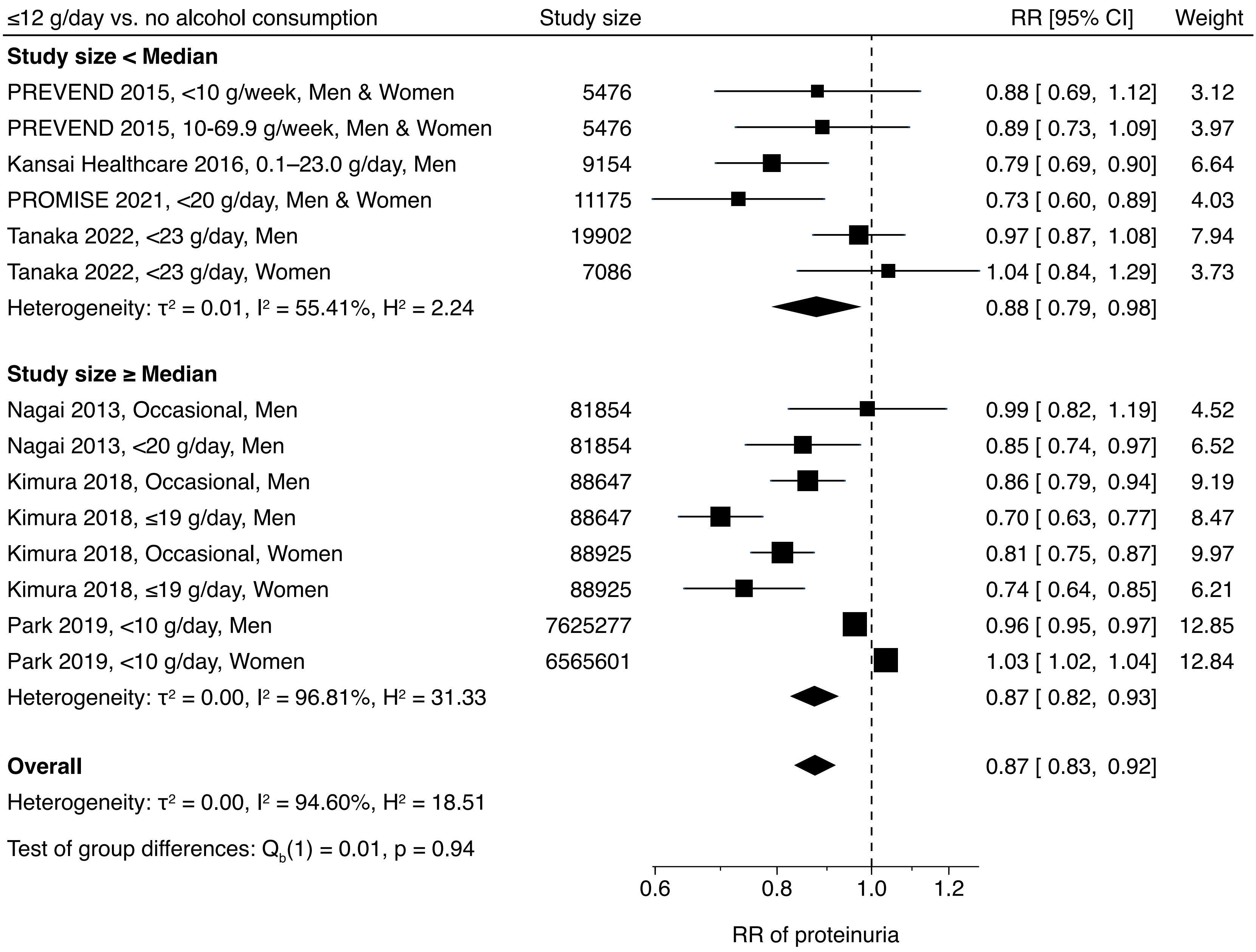

≤12 g/day vs. no alcohol consumption
Study size
RR [95% CI]
Weight
Study size < Median
PREVEND 2015, <10 g/week, Men & Women 5476 0.88 [0.69, 1.12] 3.12
PREVEND 2015, 10-69.9 g/week, Men & Women 5476 0.89 [0.73, 1.09] 3.97
Kansai Healthcare 2016, 0.1–23.0 g/day, Men 9154 0.79 [0.69, 0.90] 6.64
PROMISE 2021, <20 g/day, Men & Women 11175 0.73 [0.60, 0.89] 4.03
Tanaka 2022, <23 g/day, Men 19902 0.97 [0.87, 1.08] 7.94
Tanaka 2022, <23 g/day, Women 7086 1.04 [0.84, 1.29] 3.73
Heterogeneity: τ² = 0.01, I² = 55.41%, H² = 2.24 0.88 [0.79, 0.98]
Study size ≥ Median
Nagai 2013, Occasional, Men 81854 0.99 [0.82, 1.19] 4.52
Nagai 2013, <20 g/day, Men 81854 0.85 [0.74, 0.97] 6.52
Kimura 2018, Occasional, Men 88647 0.86 [0.79, 0.94] 9.19
Kimura 2018, ≤19 g/day, Men 88647 0.70 [0.63, 0.77] 8.47
Kimura 2018, Occasional, Women 88925 0.81 [0.75, 0.87] 9.97
Kimura 2018, ≤19 g/day, Women 88925 0.74 [0.64, 0.85] 6.21
Park 2019, <10 g/day, Men 7625277 0.96 [0.95, 0.97] 12.85
Park 2019, <10 g/day, Women 6565601 1.03 [1.02, 1.04] 12.84
Heterogeneity: τ² = 0.00, I² = 96.81%, H² = 31.33 0.87 [0.82, 0.93]
Overall 0.87 [0.83, 0.92]
Heterogeneity: τ² = 0.00, I² = 94.60%, H² = 18.51
Test of group differences: Q_b(1) = 0.01, p = 0.94
0.6
0.8
1.0
1.2
RR of proteinuria

Figure S3c

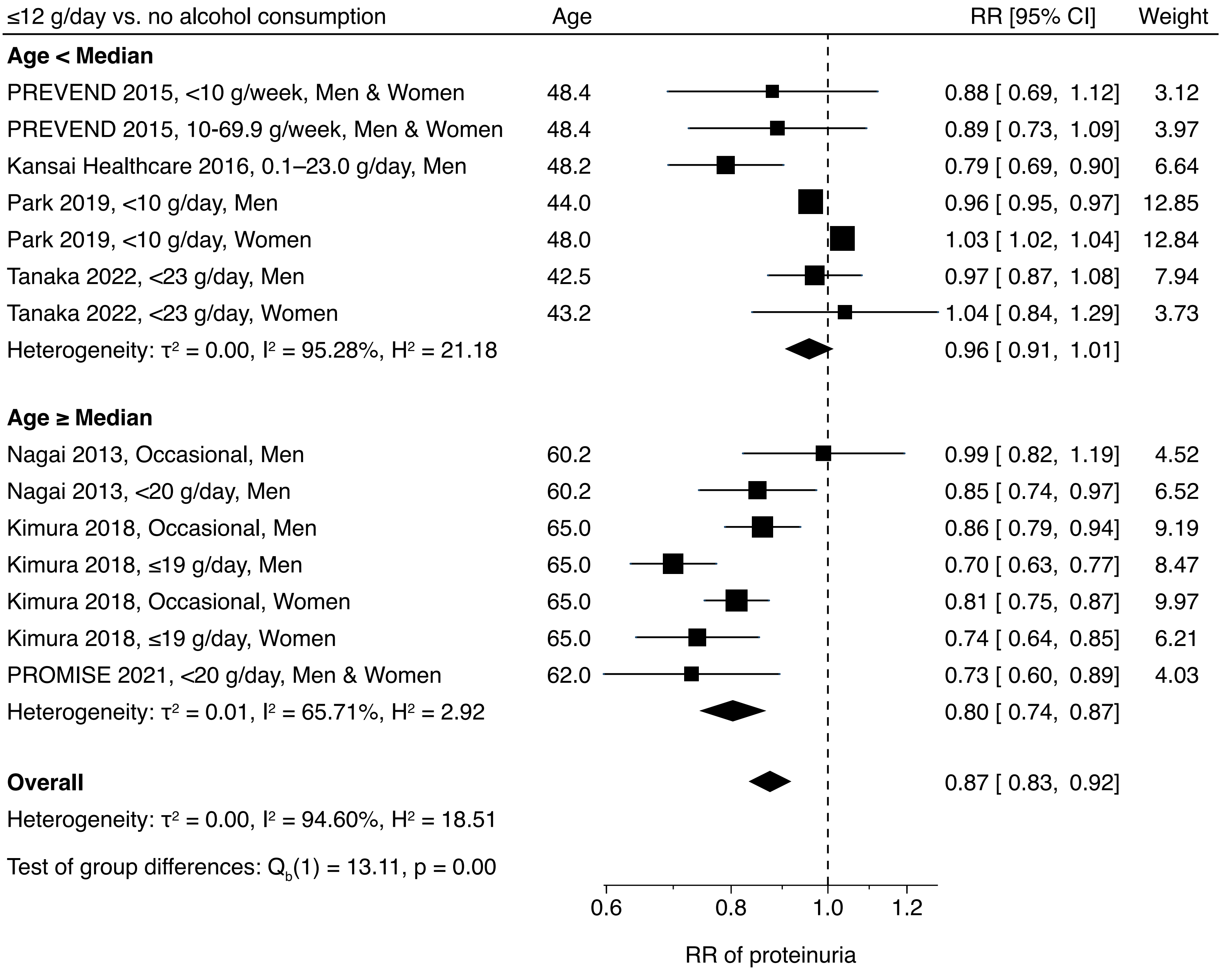

≤12 g/day vs. no alcohol consumption
Age
RR [95% CI]
Weight
Age < Median
PREVEND 2015, <10 g/week, Men & Women 48.4 0.88 [0.69, 1.12] 3.12
PREVEND 2015, 10-69.9 g/week, Men & Women 48.4 0.89 [0.73, 1.09] 3.97
Kansai Healthcare 2016, 0.1–23.0 g/day, Men 48.2 0.79 [0.69, 0.90] 6.64
Park 2019, <10 g/day, Men 44.0 0.96 [0.95, 0.97] 12.85
Park 2019, <10 g/day, Women 48.0 1.03 [1.02, 1.04] 12.84
Tanaka 2022, <23 g/day, Men 42.5 0.97 [0.87, 1.08] 7.94
Tanaka 2022, <23 g/day, Women 43.2 1.04 [0.84, 1.29] 3.73
Heterogeneity: τ² = 0.00, I² = 95.28%, H² = 21.18 0.96 [0.91, 1.01]
Age ≥ Median
Nagai 2013, Occasional, Men 60.2 0.99 [0.82, 1.19] 4.52
Nagai 2013, <20 g/day, Men 60.2 0.85 [0.74, 0.97] 6.52
Kimura 2018, Occasional, Men 65.0 0.86 [0.79, 0.94] 9.19
Kimura 2018, ≤19 g/day, Men 65.0 0.70 [0.63, 0.77] 8.47
Kimura 2018, Occasional, Women 65.0 0.81 [0.75, 0.87] 9.97
Kimura 2018, ≤19 g/day, Women 65.0 0.74 [0.64, 0.85] 6.21
PROMISE 2021, <20 g/day, Men & Women 62.0 0.73 [0.60, 0.89] 4.03
Heterogeneity: τ² = 0.01, I² = 65.71%, H² = 2.92 0.80 [0.74, 0.87]
Overall 0.87 [0.83, 0.92]
Heterogeneity: τ² = 0.00, I² = 94.60%, H² = 18.51
Test of group differences: Q_b(1) = 13.11, p = 0.00
0.6
0.8
1.0
1.2
RR of proteinuria

Figure S3d

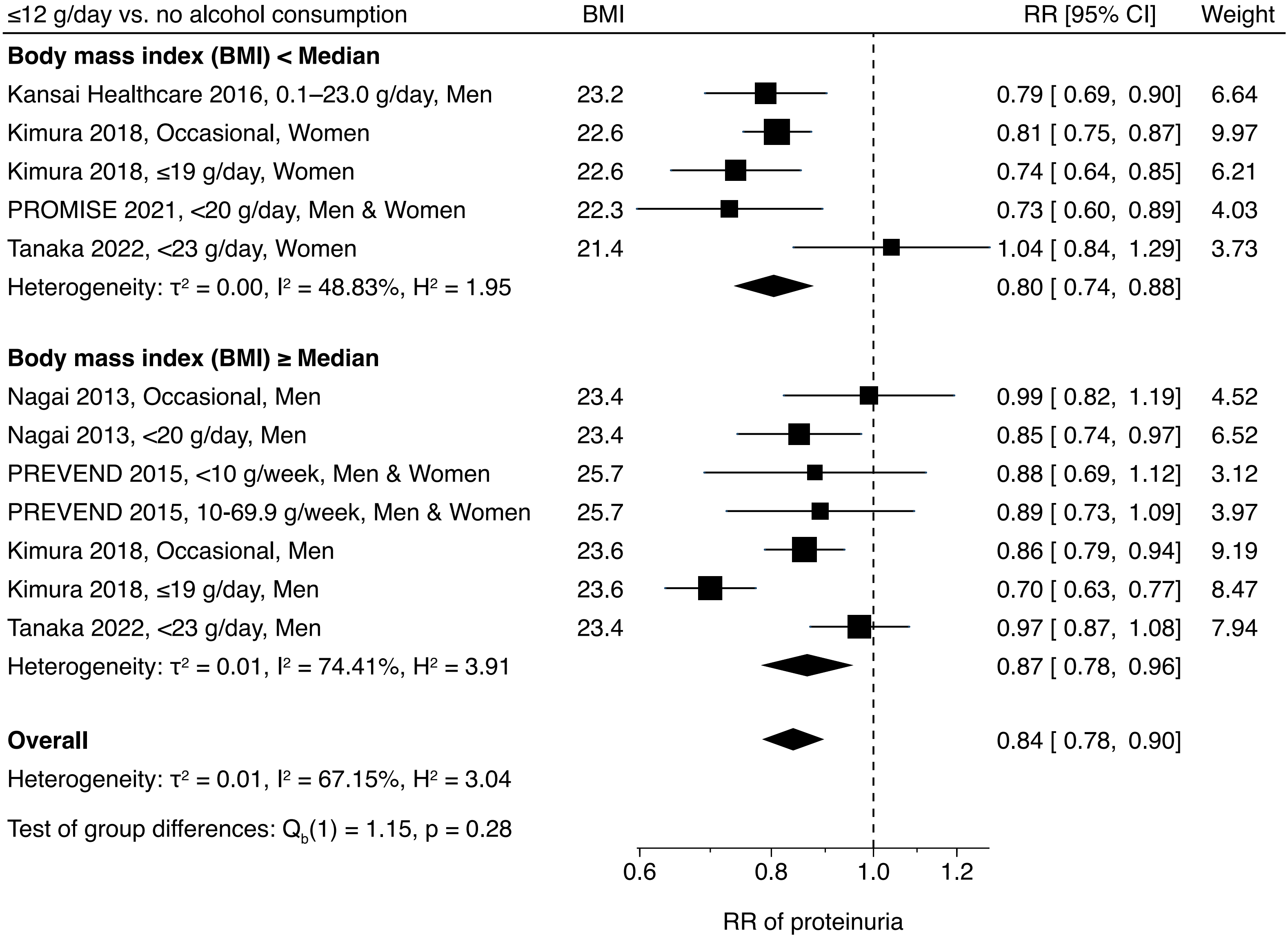
≤12 g/day vs. no alcohol consumption
BMI
RR [95% CI]
Weight
Body mass index (BMI) < Median
Kansai Healthcare 2016, 0.1–23.0 g/day, Men 23.2 0.79 [0.69, 0.90] 6.64
Kimura 2018, Occasional, Women 22.6 0.81 [0.75, 0.87] 9.97
Kimura 2018, ≤19 g/day, Women 22.6 0.74 [0.64, 0.85] 6.21
PROMISE 2021, <20 g/day, Men & Women 22.3 0.73 [0.60, 0.89] 4.03
Tanaka 2022, <23 g/day, Women 21.4 1.04 [0.84, 1.29] 3.73
Heterogeneity: τ² = 0.00, I² = 48.83%, H² = 1.95 0.80 [0.74, 0.88]
Body mass index (BMI) ≥ Median
Nagai 2013, Occasional, Men 23.4 0.99 [0.82, 1.19] 4.52
Nagai 2013, <20 g/day, Men 23.4 0.85 [0.74, 0.97] 6.52
PREVEND 2015, <10 g/week, Men & Women 25.7 0.88 [0.69, 1.12] 3.12
PREVEND 2015, 10-69.9 g/week, Men & Women 25.7 0.89 [0.73, 1.09] 3.97
Kimura 2018, Occasional, Men 23.6 0.86 [0.79, 0.94] 9.19
Kimura 2018, ≤19 g/day, Men 23.6 0.70 [0.63, 0.77] 8.47
Tanaka 2022, <23 g/day, Men 23.4 0.97 [0.87, 1.08] 7.94
Heterogeneity: τ² = 0.01, I² = 74.41%, H² = 3.91 0.87 [0.78, 0.96]
Overall 0.84 [0.78, 0.90]
Heterogeneity: τ² = 0.01, I² = 67.15%, H² = 3.04
Test of group differences: Q_b(1) = 1.15, p = 0.28
0.6
0.8
1.0
1.2
RR of proteinuria

Figure S3e

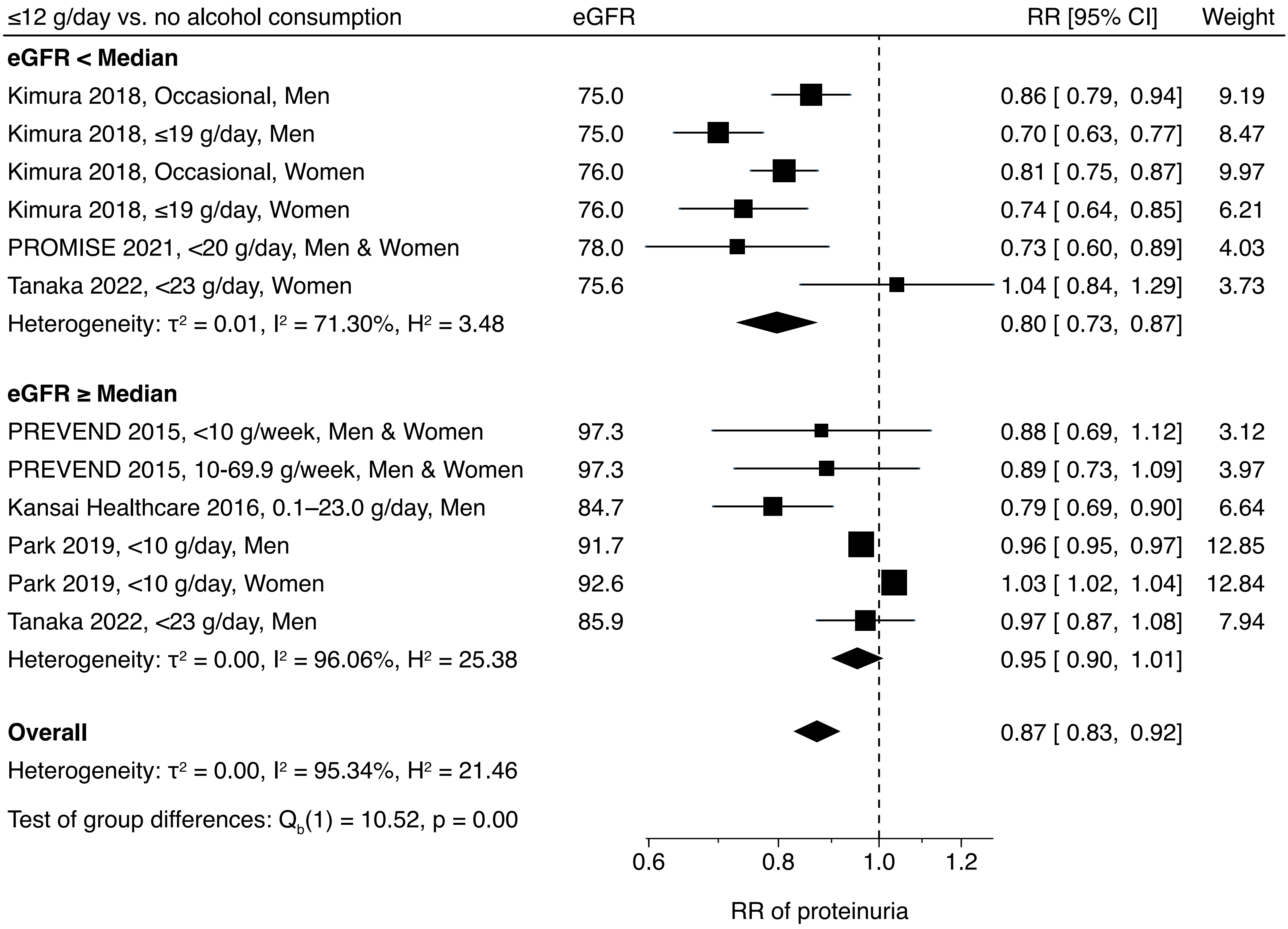
≤12 g/day vs. no alcohol consumption
eGFR
RR [95% CI]
Weight
eGFR < Median
Kimura 2018, Occasional, Men 75.0 0.86 [0.79, 0.94] 9.19
Kimura 2018, ≤19 g/day, Men 75.0 0.70 [0.63, 0.77] 8.47
Kimura 2018, Occasional, Women 76.0 0.81 [0.75, 0.87] 9.97
Kimura 2018, ≤19 g/day, Women 76.0 0.74 [0.64, 0.85] 6.21
PROMISE 2021, <20 g/day, Men & Women 78.0 0.73 [0.60, 0.89] 4.03
Tanaka 2022, <23 g/day, Women 75.6 1.04 [0.84, 1.29] 3.73
Heterogeneity: τ² = 0.01, I² = 71.30%, H² = 3.48 0.80 [0.73, 0.87]
eGFR ≥ Median
PREVEND 2015, <10 g/week, Men & Women 97.3 0.88 [0.69, 1.12] 3.12
PREVEND 2015, 10-69.9 g/week, Men & Women 97.3 0.89 [0.73, 1.09] 3.97
Kansai Healthcare 2016, 0.1–23.0 g/day, Men 84.7 0.79 [0.69, 0.90] 6.64
Park 2019, <10 g/day, Men 91.7 0.96 [0.95, 0.97] 12.85
Park 2019, <10 g/day, Women 92.6 1.03 [1.02, 1.04] 12.84
Tanaka 2022, <23 g/day, Men 85.9 0.97 [0.87, 1.08] 7.94
Heterogeneity: τ² = 0.00, I² = 96.06%, H² = 25.38 0.95 [0.90, 1.01]
Overall 0.87 [0.83, 0.92]
Heterogeneity: τ² = 0.00, I² = 95.34%, H² = 21.46
Test of group differences: Qb(1) = 10.52, p = 0.00
0.6
0.8
1.0
1.2
RR of proteinuria

Figure S3f

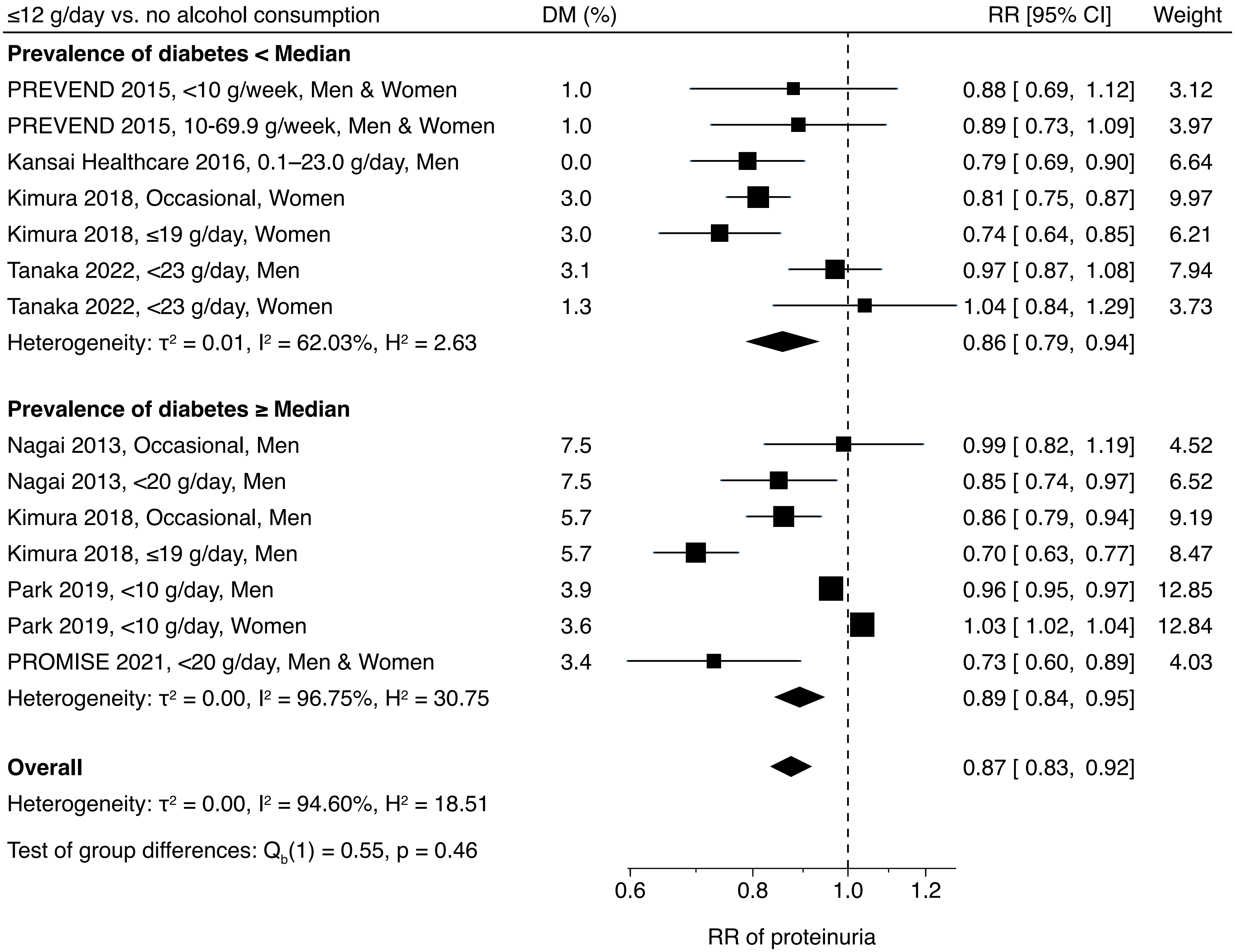

≤12 g/day vs. no alcohol consumption
DM (%)
RR [95% CI]
Weight
Prevalence of diabetes < Median
PREVEND 2015, <10 g/week, Men & Women 1.0 0.88 [0.69, 1.12] 3.12
PREVEND 2015, 10-69.9 g/week, Men & Women 1.0 0.89 [0.73, 1.09] 3.97
Kansai Healthcare 2016, 0.1–23.0 g/day, Men 0.0 0.79 [0.69, 0.90] 6.64
Kimura 2018, Occasional, Women 3.0 0.81 [0.75, 0.87] 9.97
Kimura 2018, ≤19 g/day, Women 3.0 0.74 [0.64, 0.85] 6.21
Tanaka 2022, <23 g/day, Men 3.1 0.97 [0.87, 1.08] 7.94
Tanaka 2022, <23 g/day, Women 1.3 1.04 [0.84, 1.29] 3.73
Heterogeneity: $\tau^2 = 0.01$, $I^2 = 62.03\%$, $H^2 = 2.63$ 0.86 [0.79, 0.94]
Prevalence of diabetes ≥ Median
Nagai 2013, Occasional, Men 7.5 0.99 [0.82, 1.19] 4.52
Nagai 2013, <20 g/day, Men 7.5 0.85 [0.74, 0.97] 6.52
Kimura 2018, Occasional, Men 5.7 0.86 [0.79, 0.94] 9.19
Kimura 2018, ≤19 g/day, Men 5.7 0.70 [0.63, 0.77] 8.47
Park 2019, <10 g/day, Men 3.9 0.96 [0.95, 0.97] 12.85
Park 2019, <10 g/day, Women 3.6 1.03 [1.02, 1.04] 12.84
PROMISE 2021, <20 g/day, Men & Women 3.4 0.73 [0.60, 0.89] 4.03
Heterogeneity: $\tau^2 = 0.00$, $I^2 = 96.75\%$, $H^2 = 30.75$ 0.89 [0.84, 0.95]
Overall 0.87 [0.83, 0.92]
Heterogeneity: $\tau^2 = 0.00$, $I^2 = 94.60\%$, $H^2 = 18.51$
Test of group differences: $Q_b(1) = 0.55$, $p = 0.46$
0.6
0.8
1.0
1.2
RR of proteinuria

Figure S3g

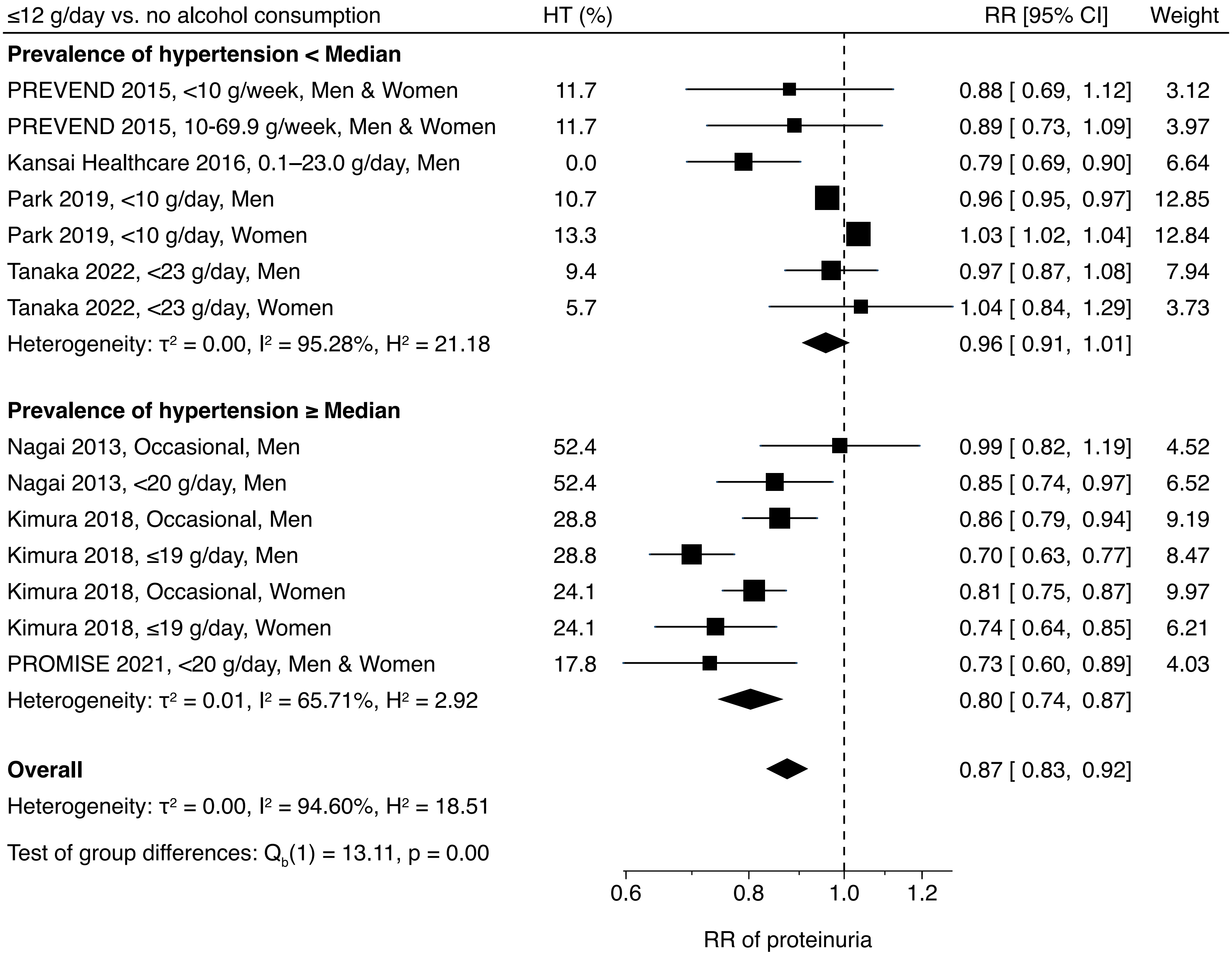
≤12 g/day vs. no alcohol consumption
HT (%)
RR [95% CI]
Weight
Prevalence of hypertension < Median
PREVEND 2015, <10 g/week, Men & Women 11.7 0.88 [0.69, 1.12] 3.12
PREVEND 2015, 10-69.9 g/week, Men & Women 11.7 0.89 [0.73, 1.09] 3.97
Kansai Healthcare 2016, 0.1–23.0 g/day, Men 0.0 0.79 [0.69, 0.90] 6.64
Park 2019, <10 g/day, Men 10.7 0.96 [0.95, 0.97] 12.85
Park 2019, <10 g/day, Women 13.3 1.03 [1.02, 1.04] 12.84
Tanaka 2022, <23 g/day, Men 9.4 0.97 [0.87, 1.08] 7.94
Tanaka 2022, <23 g/day, Women 5.7 1.04 [0.84, 1.29] 3.73
Heterogeneity: τ² = 0.00, I² = 95.28%, H² = 21.18 0.96 [0.91, 1.01]
Prevalence of hypertension ≥ Median
Nagai 2013, Occasional, Men 52.4 0.99 [0.82, 1.19] 4.52
Nagai 2013, <20 g/day, Men 52.4 0.85 [0.74, 0.97] 6.52
Kimura 2018, Occasional, Men 28.8 0.86 [0.79, 0.94] 9.19
Kimura 2018, ≤19 g/day, Men 28.8 0.70 [0.63, 0.77] 8.47
Kimura 2018, Occasional, Women 24.1 0.81 [0.75, 0.87] 9.97
Kimura 2018, ≤19 g/day, Women 24.1 0.74 [0.64, 0.85] 6.21
PROMISE 2021, <20 g/day, Men & Women 17.8 0.73 [0.60, 0.89] 4.03
Heterogeneity: τ² = 0.01, I² = 65.71%, H² = 2.92 0.80 [0.74, 0.87]
Overall 0.87 [0.83, 0.92]
Heterogeneity: τ² = 0.00, I² = 94.60%, H² = 18.51
Test of group differences: Qb(1) = 13.11, p = 0.00
0.6
0.8
1.0
1.2
RR of proteinuria

Figure S3h

≤12 g/day vs. no alcohol consumption	Follow-up	RR [95% CI]	Weight
**Follow-up duration < Median**			
Nagai 2013, Occasional, Men	4.0	0.99 [0.82, 1.19]	4.52
Nagai 2013, <20 g/day, Men	4.0	0.85 [0.74, 0.97]	6.52
Kimura 2018, Occasional, Men	1.8	0.86 [0.79, 0.94]	9.19
Kimura 2018, ≤19 g/day, Men	1.8	0.70 [0.63, 0.77]	8.47
Kimura 2018, Occasional, Women	1.7	0.81 [0.75, 0.87]	9.97
Kimura 2018, ≤19 g/day, Women	1.7	0.74 [0.64, 0.85]	6.21
Tanaka 2022, <23 g/day, Men	4.0	0.97 [0.87, 1.08]	7.94
Tanaka 2022, <23 g/day, Women	4.0	1.04 [0.84, 1.29]	3.73
Heterogeneity: $\tau^2 = 0.01$, $I^2 = 77.23\%$, $H^2 = 4.39$		0.85 [0.78, 0.93]	
**Follow-up duration ≥ Median**			
PREVEND 2015, <10 g/week, Men & Women	10.2	0.88 [0.69, 1.12]	3.12
PREVEND 2015, 10-69.9 g/week, Men & Women	10.2	0.89 [0.73, 1.09]	3.97
Kansai Healthcare 2016, 0.1–23.0 g/day, Men	8.0	0.79 [0.69, 0.90]	6.64
Park 2019, <10 g/day, Men	6.4	0.96 [0.95, 0.97]	12.85
Park 2019, <10 g/day, Women	6.4	1.03 [1.02, 1.04]	12.84
PROMISE 2021, <20 g/day, Men & Women	5.0	0.73 [0.60, 0.89]	4.03
Heterogeneity: $\tau^2 = 0.00$, $I^2 = 96.31\%$, $H^2 = 27.10$		0.93 [0.87, 0.99]	
**Overall**		0.87 [0.83, 0.92]	
Heterogeneity: $\tau^2 = 0.00$, $I^2 = 94.60\%$, $H^2 = 18.51$			
Test of group differences: $Q_b(1) = 2.71$, $p = 0.10$			

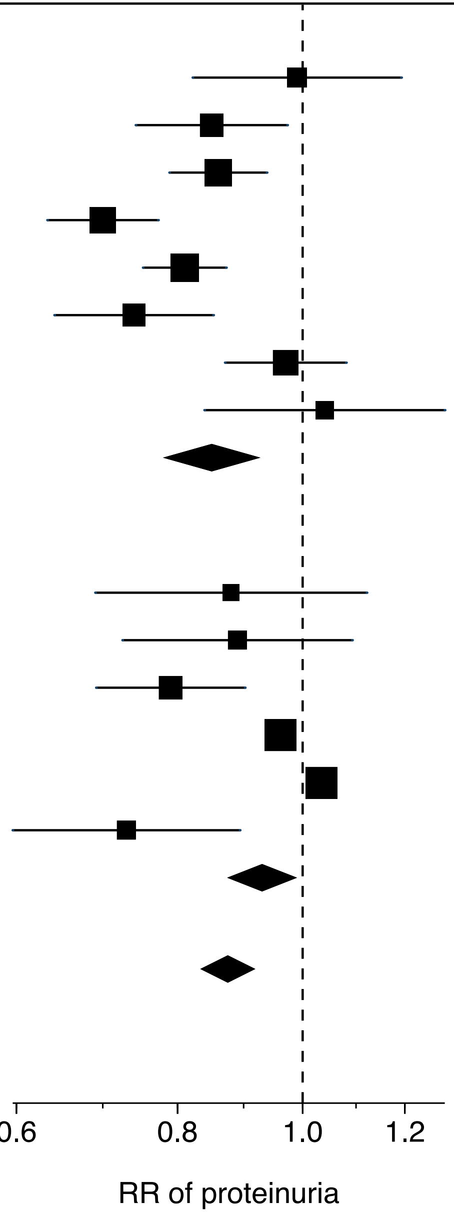

Figure S3i

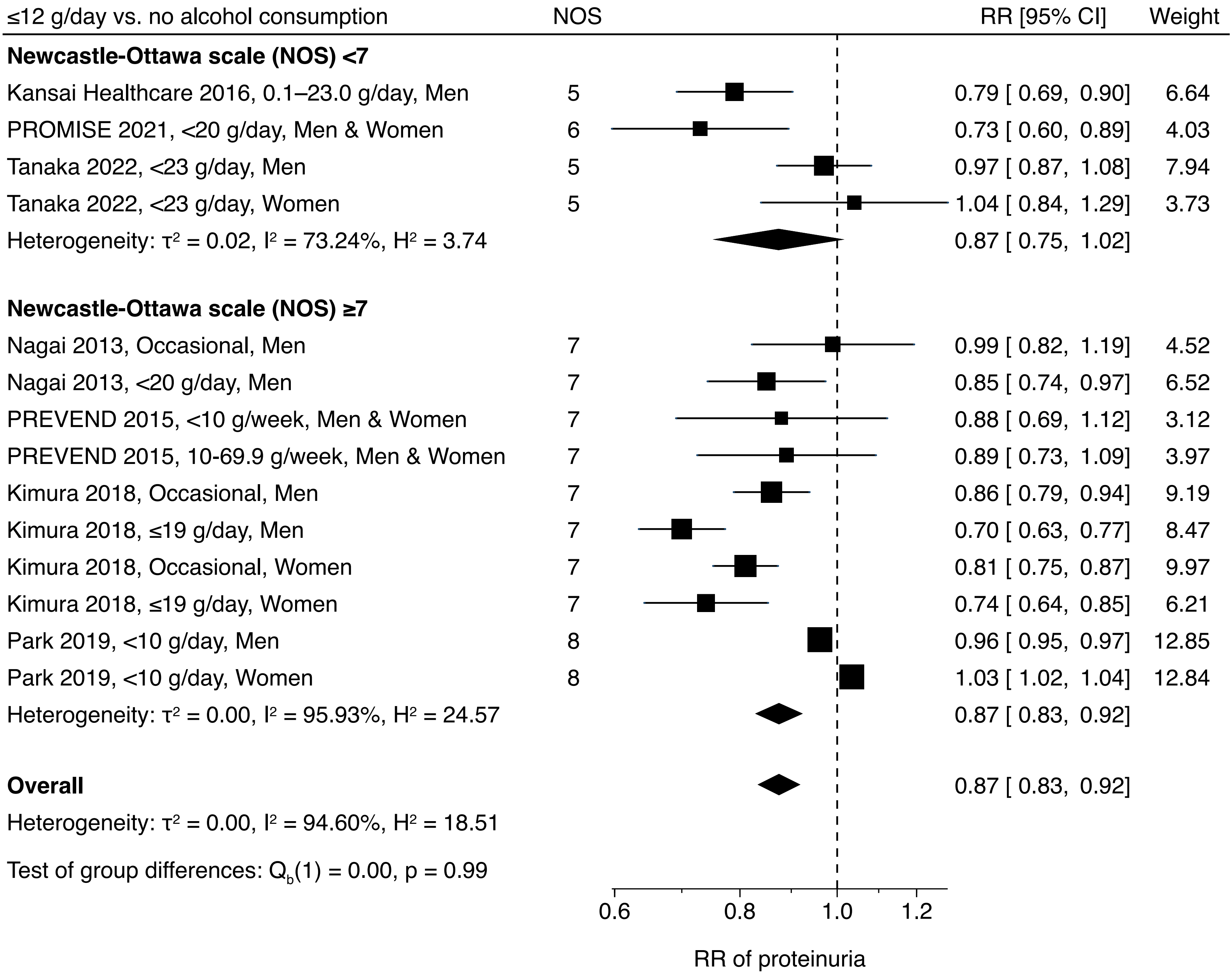
≤12 g/day vs. no alcohol consumption
NOS
RR [95% CI]
Weight
Newcastle-Ottawa scale (NOS) <7
Kansai Healthcare 2016, 0.1–23.0 g/day, Men 5 0.79 [0.69, 0.90] 6.64
PROMISE 2021, <20 g/day, Men & Women 6 0.73 [0.60, 0.89] 4.03
Tanaka 2022, <23 g/day, Men 5 0.97 [0.87, 1.08] 7.94
Tanaka 2022, <23 g/day, Women 5 1.04 [0.84, 1.29] 3.73
Heterogeneity: τ² = 0.02, I² = 73.24%, H² = 3.74 0.87 [0.75, 1.02]
Newcastle-Ottawa scale (NOS) ≥7
Nagai 2013, Occasional, Men 7 0.99 [0.82, 1.19] 4.52
Nagai 2013, <20 g/day, Men 7 0.85 [0.74, 0.97] 6.52
PREVEND 2015, <10 g/week, Men & Women 7 0.88 [0.69, 1.12] 3.12
PREVEND 2015, 10-69.9 g/week, Men & Women 7 0.89 [0.73, 1.09] 3.97
Kimura 2018, Occasional, Men 7 0.86 [0.79, 0.94] 9.19
Kimura 2018, ≤19 g/day, Men 7 0.70 [0.63, 0.77] 8.47
Kimura 2018, Occasional, Women 7 0.81 [0.75, 0.87] 9.97
Kimura 2018, ≤19 g/day, Women 7 0.74 [0.64, 0.85] 6.21
Park 2019, <10 g/day, Men 8 0.96 [0.95, 0.97] 12.85
Park 2019, <10 g/day, Women 8 1.03 [1.02, 1.04] 12.84
Heterogeneity: τ² = 0.00, I² = 95.93%, H² = 24.57 0.87 [0.83, 0.92]
Overall 0.87 [0.83, 0.92]
Heterogeneity: τ² = 0.00, I² = 94.60%, H² = 18.51
Test of group differences: Qb(1) = 0.00, p = 0.99
0.6
0.8
1.0
1.2
RR of proteinuria

Figure S3j

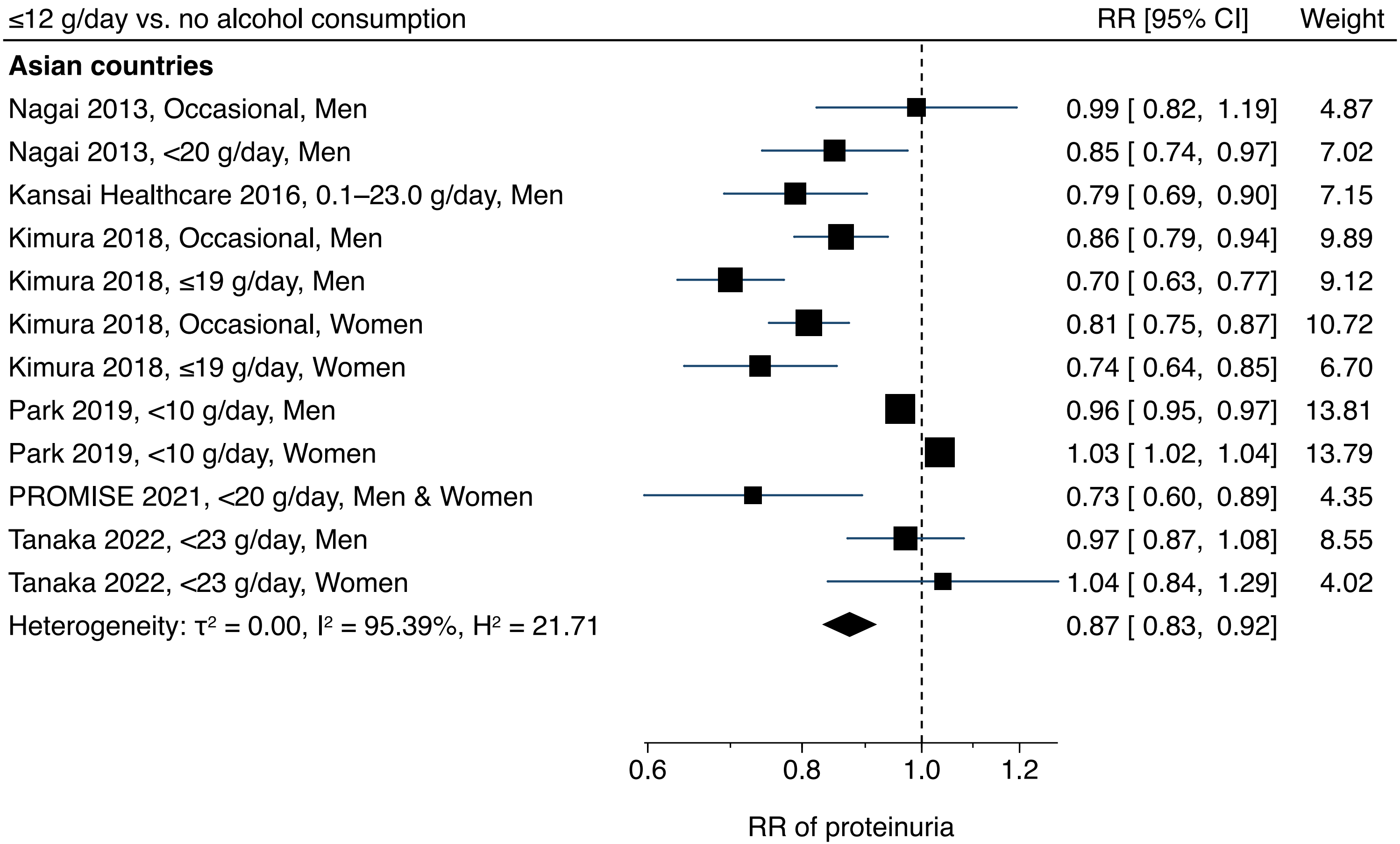
≤12 g/day vs. no alcohol consumption
RR [95% CI]
Weight
Asian countries
Nagai 2013, Occasional, Men
0.99 [0.82, 1.19]
4.87
Nagai 2013, <20 g/day, Men
0.85 [0.74, 0.97]
7.02
Kansai Healthcare 2016, 0.1–23.0 g/day, Men
0.79 [0.69, 0.90]
7.15
Kimura 2018, Occasional, Men
0.86 [0.79, 0.94]
9.89
Kimura 2018, ≤19 g/day, Men
0.70 [0.63, 0.77]
9.12
Kimura 2018, Occasional, Women
0.81 [0.75, 0.87]
10.72
Kimura 2018, ≤19 g/day, Women
0.74 [0.64, 0.85]
6.70
Park 2019, <10 g/day, Men
0.96 [0.95, 0.97]
13.81
Park 2019, <10 g/day, Women
1.03 [1.02, 1.04]
13.79
PROMISE 2021, <20 g/day, Men & Women
0.73 [0.60, 0.89]
4.35
Tanaka 2022, <23 g/day, Men
0.97 [0.87, 1.08]
8.55
Tanaka 2022, <23 g/day, Women
1.04 [0.84, 1.29]
4.02
Heterogeneity: τ² = 0.00, I² = 95.39%, H² = 21.71
0.87 [0.83, 0.92]
0.6
0.8
1.0
1.2
RR of proteinuria

Figure S4a

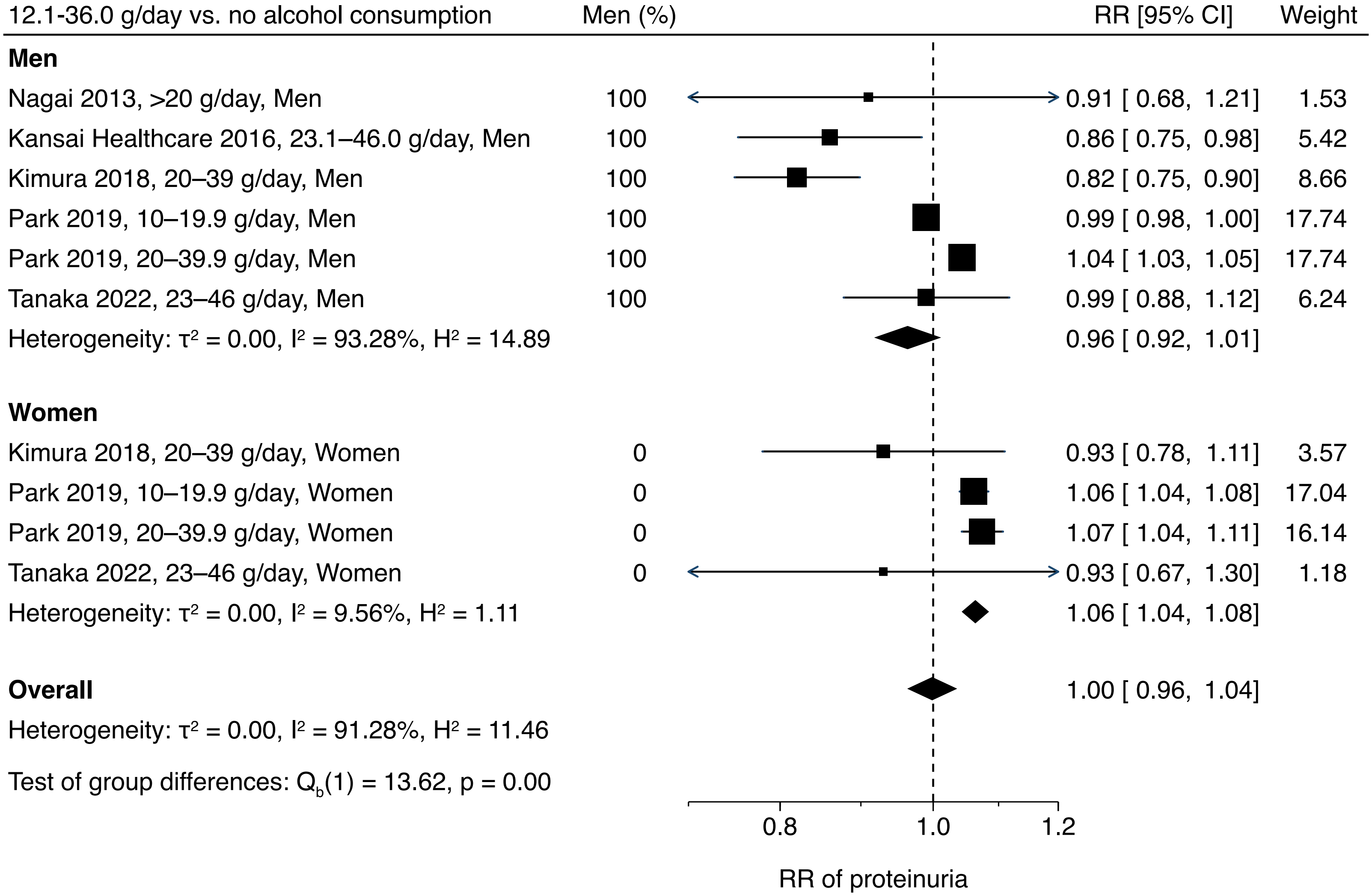
12.1-36.0 g/day vs. no alcohol consumption
Men (%)
RR [95% CI]
Weight
Men
Nagai 2013, >20 g/day, Men 100 0.91 [0.68, 1.21] 1.53
Kansai Healthcare 2016, 23.1–46.0 g/day, Men 100 0.86 [0.75, 0.98] 5.42
Kimura 2018, 20–39 g/day, Men 100 0.82 [0.75, 0.90] 8.66
Park 2019, 10–19.9 g/day, Men 100 0.99 [0.98, 1.00] 17.74
Park 2019, 20–39.9 g/day, Men 100 1.04 [1.03, 1.05] 17.74
Tanaka 2022, 23–46 g/day, Men 100 0.99 [0.88, 1.12] 6.24
Heterogeneity: τ^2 = 0.00, I^2 = 93.28%, H^2 = 14.89 0.96 [0.92, 1.01]
Women
Kimura 2018, 20–39 g/day, Women 0 0.93 [0.78, 1.11] 3.57
Park 2019, 10–19.9 g/day, Women 0 1.06 [1.04, 1.08] 17.04
Park 2019, 20–39.9 g/day, Women 0 1.07 [1.04, 1.11] 16.14
Tanaka 2022, 23–46 g/day, Women 0 0.93 [0.67, 1.30] 1.18
Heterogeneity: τ^2 = 0.00, I^2 = 9.56%, H^2 = 1.11 1.06 [1.04, 1.08]
Overall 1.00 [0.96, 1.04]
Heterogeneity: τ^2 = 0.00, I^2 = 91.28%, H^2 = 11.46
Test of group differences: $Q_b(1)$ = 13.62, p = 0.00
0.8
1.0
1.2
RR of proteinuria

Figure S4b

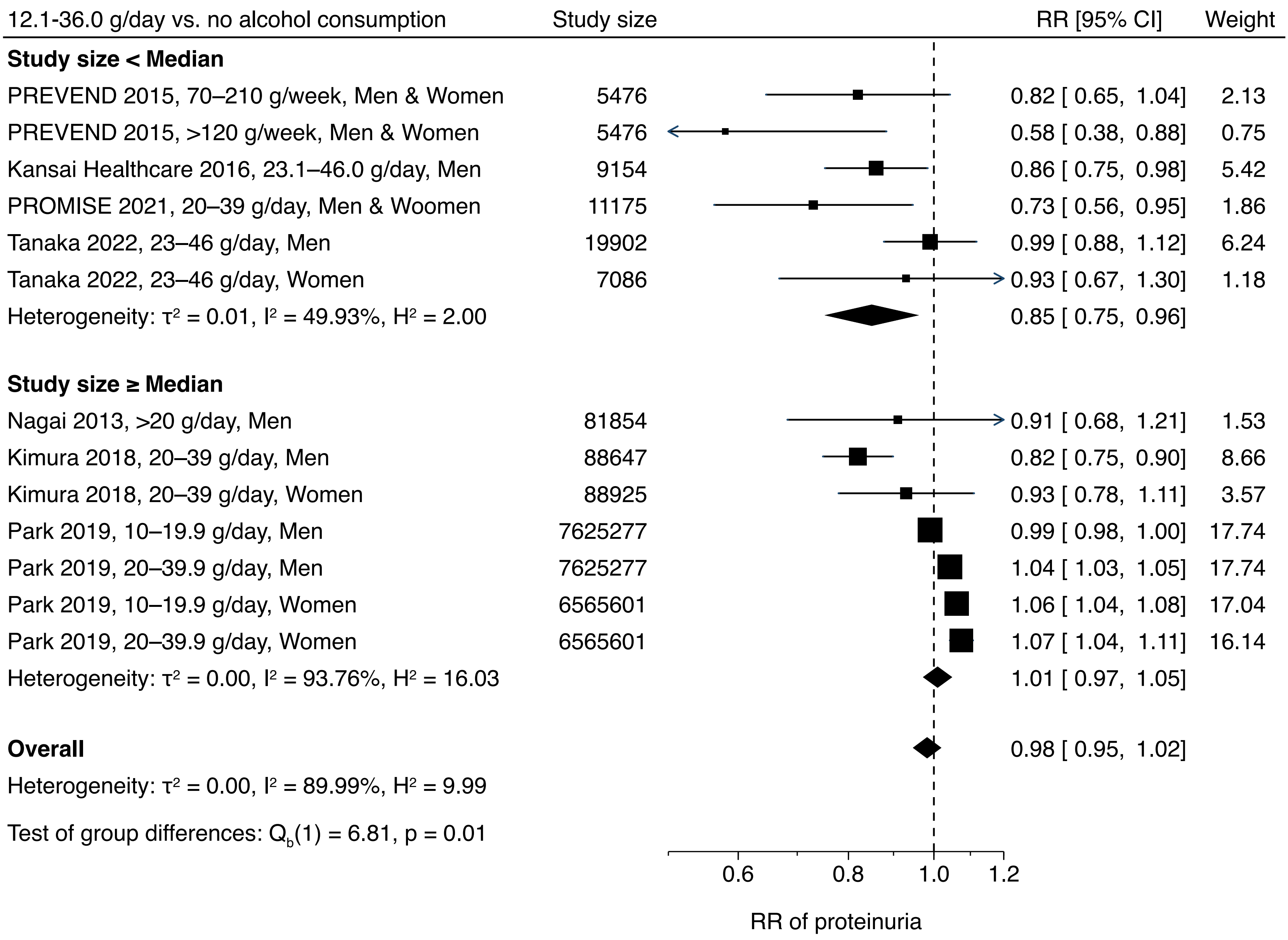
12.1-36.0 g/day vs. no alcohol consumption
Study size
RR [95% CI]
Weight
Study size < Median
PREVEND 2015, 70–210 g/week, Men & Women 5476 0.82 [0.65, 1.04] 2.13
PREVEND 2015, >120 g/week, Men & Women 5476 0.58 [0.38, 0.88] 0.75
Kansai Healthcare 2016, 23.1–46.0 g/day, Men 9154 0.86 [0.75, 0.98] 5.42
PROMISE 2021, 20–39 g/day, Men & Woomen 11175 0.73 [0.56, 0.95] 1.86
Tanaka 2022, 23–46 g/day, Men 19902 0.99 [0.88, 1.12] 6.24
Tanaka 2022, 23–46 g/day, Women 7086 0.93 [0.67, 1.30] 1.18
Heterogeneity: τ² = 0.01, I² = 49.93%, H² = 2.00 0.85 [0.75, 0.96]
Study size ≥ Median
Nagai 2013, >20 g/day, Men 81854 0.91 [0.68, 1.21] 1.53
Kimura 2018, 20–39 g/day, Men 88647 0.82 [0.75, 0.90] 8.66
Kimura 2018, 20–39 g/day, Women 88925 0.93 [0.78, 1.11] 3.57
Park 2019, 10–19.9 g/day, Men 7625277 0.99 [0.98, 1.00] 17.74
Park 2019, 20–39.9 g/day, Men 7625277 1.04 [1.03, 1.05] 17.74
Park 2019, 10–19.9 g/day, Women 6565601 1.06 [1.04, 1.08] 17.04
Park 2019, 20–39.9 g/day, Women 6565601 1.07 [1.04, 1.11] 16.14
Heterogeneity: τ² = 0.00, I² = 93.76%, H² = 16.03 1.01 [0.97, 1.05]
Overall 0.98 [0.95, 1.02]
Heterogeneity: τ² = 0.00, I² = 89.99%, H² = 9.99
Test of group differences: Q_b(1) = 6.81, p = 0.01
0.6
0.8
1.0
1.2
RR of proteinuria

Figure S4c

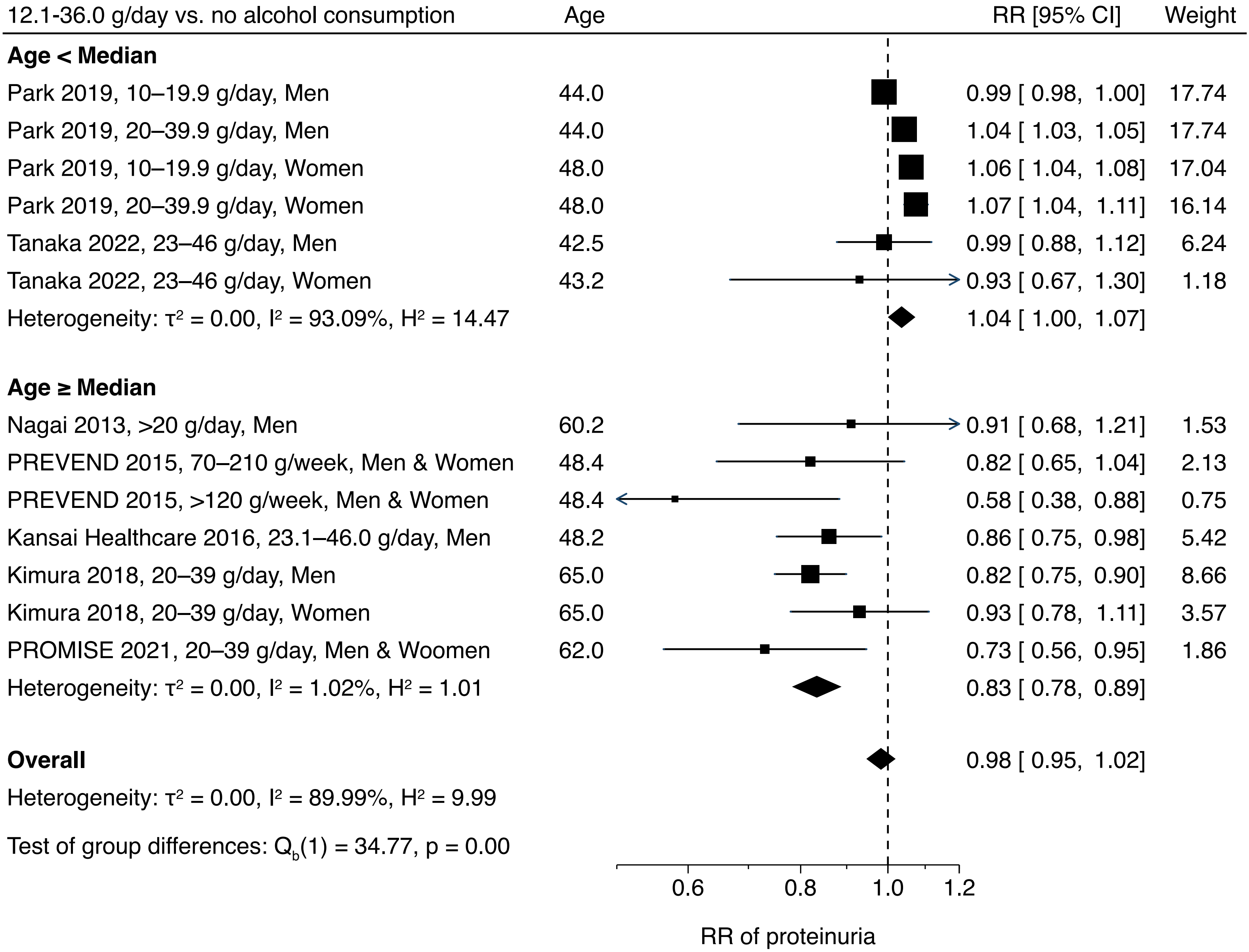

12.1-36.0 g/day vs. no alcohol consumption
Age
RR [95% CI]
Weight
Age < Median
Park 2019, 10–19.9 g/day, Men 44.0 0.99 [0.98, 1.00] 17.74
Park 2019, 20–39.9 g/day, Men 44.0 1.04 [1.03, 1.05] 17.74
Park 2019, 10–19.9 g/day, Women 48.0 1.06 [1.04, 1.08] 17.04
Park 2019, 20–39.9 g/day, Women 48.0 1.07 [1.04, 1.11] 16.14
Tanaka 2022, 23–46 g/day, Men 42.5 0.99 [0.88, 1.12] 6.24
Tanaka 2022, 23–46 g/day, Women 43.2 0.93 [0.67, 1.30] 1.18
Heterogeneity: τ^2 = 0.00, I^2 = 93.09%, H^2 = 14.47 1.04 [1.00, 1.07]
Age ≥ Median
Nagai 2013, >20 g/day, Men 60.2 0.91 [0.68, 1.21] 1.53
PREVEND 2015, 70–210 g/week, Men & Women 48.4 0.82 [0.65, 1.04] 2.13
PREVEND 2015, >120 g/week, Men & Women 48.4 0.58 [0.38, 0.88] 0.75
Kansai Healthcare 2016, 23.1–46.0 g/day, Men 48.2 0.86 [0.75, 0.98] 5.42
Kimura 2018, 20–39 g/day, Men 65.0 0.82 [0.75, 0.90] 8.66
Kimura 2018, 20–39 g/day, Women 65.0 0.93 [0.78, 1.11] 3.57
PROMISE 2021, 20–39 g/day, Men & Woomen 62.0 0.73 [0.56, 0.95] 1.86
Heterogeneity: τ^2 = 0.00, I^2 = 1.02%, H^2 = 1.01 0.83 [0.78, 0.89]
Overall 0.98 [0.95, 1.02]
Heterogeneity: τ^2 = 0.00, I^2 = 89.99%, H^2 = 9.99
Test of group differences: $Q_b(1)$ = 34.77, p = 0.00
0.6
0.8
1.0
1.2
RR of proteinuria

Figure S4d

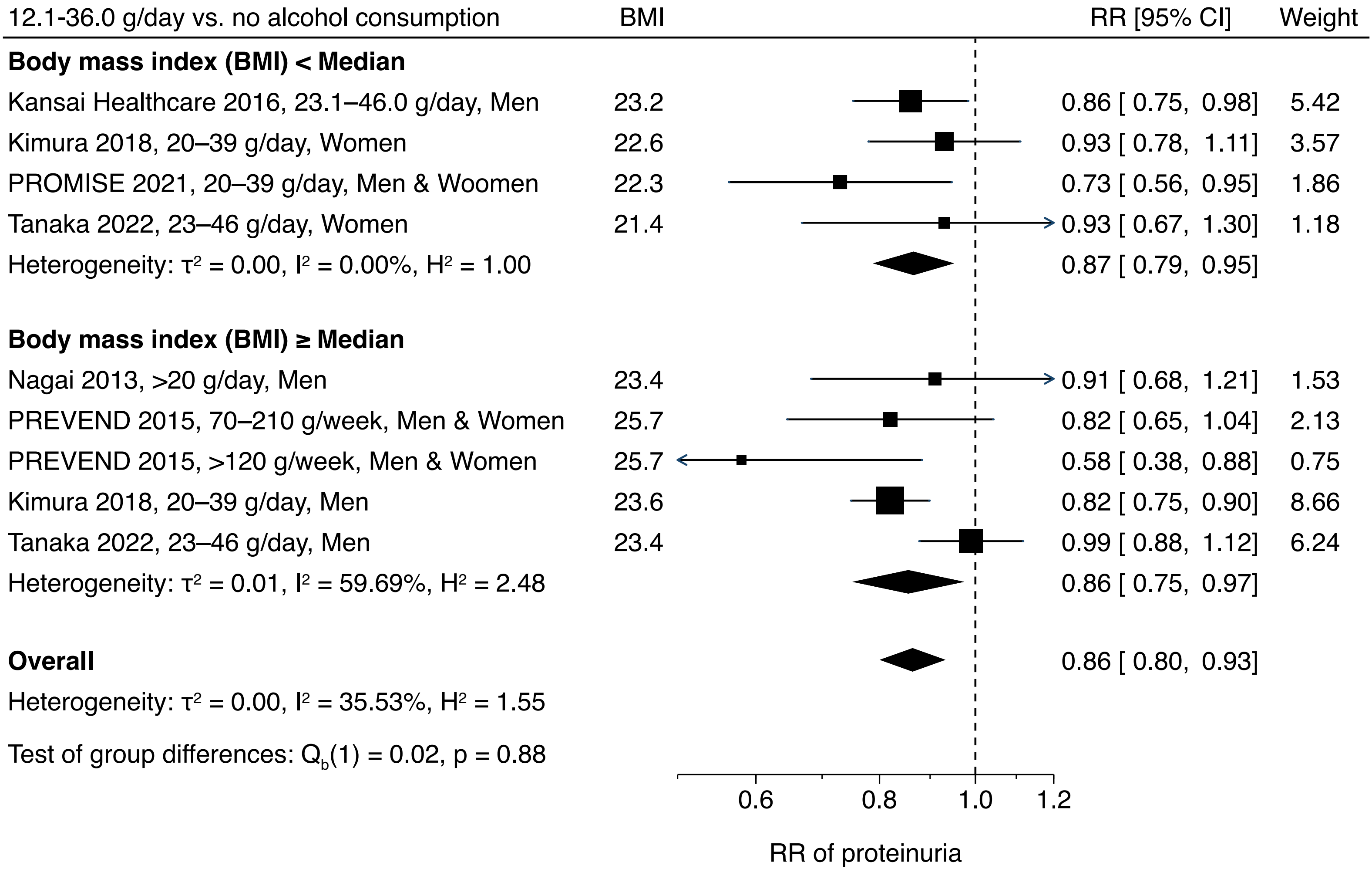
12.1-36.0 g/day vs. no alcohol consumption
BMI
RR [95% CI]
Weight
Body mass index (BMI) < Median
Kansai Healthcare 2016, 23.1–46.0 g/day, Men 23.2 0.86 [0.75, 0.98] 5.42
Kimura 2018, 20–39 g/day, Women 22.6 0.93 [0.78, 1.11] 3.57
PROMISE 2021, 20–39 g/day, Men & Woomen 22.3 0.73 [0.56, 0.95] 1.86
Tanaka 2022, 23–46 g/day, Women 21.4 0.93 [0.67, 1.30] 1.18
Heterogeneity: τ² = 0.00, I² = 0.00%, H² = 1.00 0.87 [0.79, 0.95]
Body mass index (BMI) ≥ Median
Nagai 2013, >20 g/day, Men 23.4 0.91 [0.68, 1.21] 1.53
PREVEND 2015, 70–210 g/week, Men & Women 25.7 0.82 [0.65, 1.04] 2.13
PREVEND 2015, >120 g/week, Men & Women 25.7 0.58 [0.38, 0.88] 0.75
Kimura 2018, 20–39 g/day, Men 23.6 0.82 [0.75, 0.90] 8.66
Tanaka 2022, 23–46 g/day, Men 23.4 0.99 [0.88, 1.12] 6.24
Heterogeneity: τ² = 0.01, I² = 59.69%, H² = 2.48 0.86 [0.75, 0.97]
Overall 0.86 [0.80, 0.93]
Heterogeneity: τ² = 0.00, I² = 35.53%, H² = 1.55
Test of group differences: Qb(1) = 0.02, p = 0.88
0.6
0.8
1.0
1.2
RR of proteinuria

Figure S4e

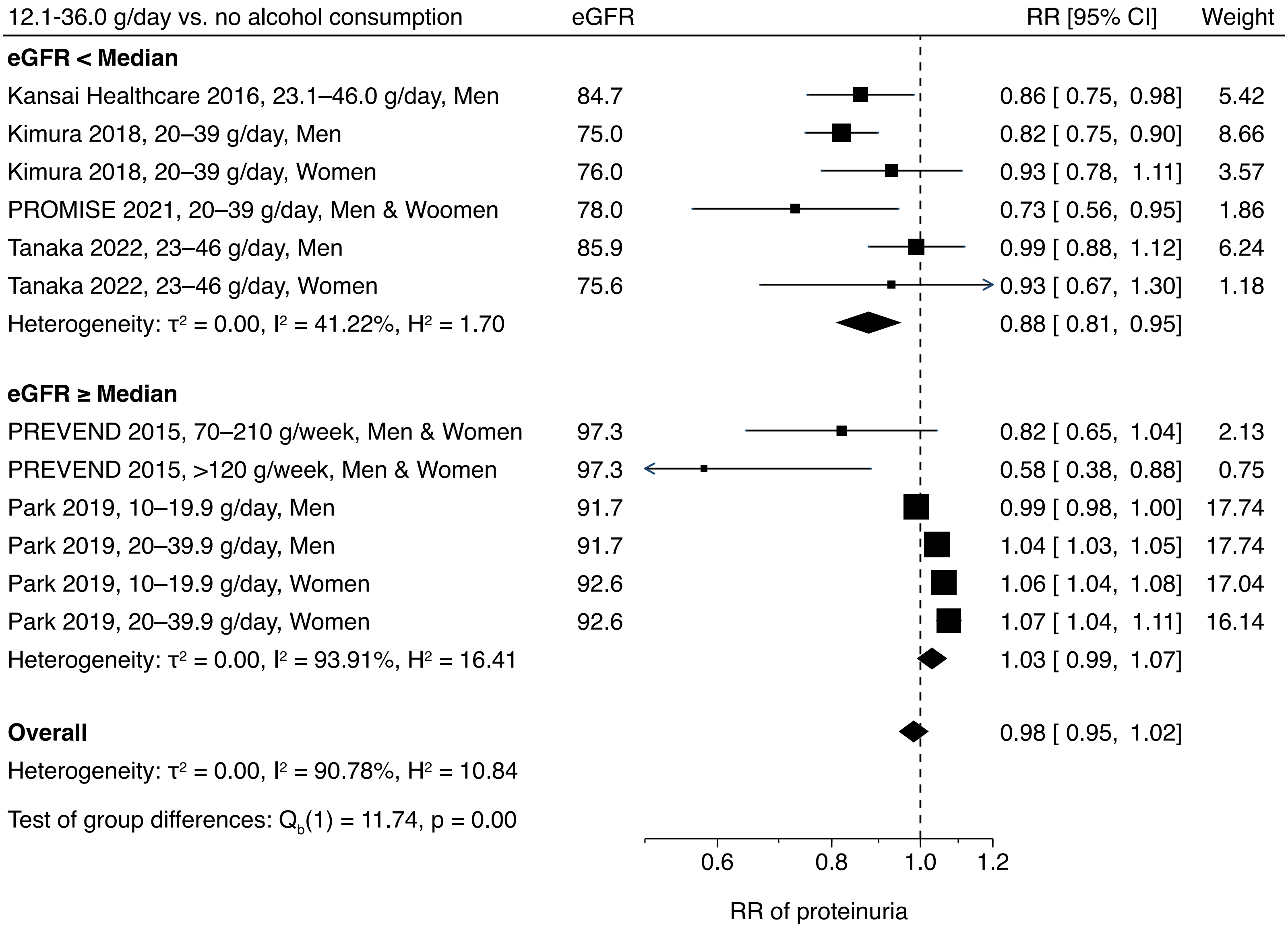
12.1-36.0 g/day vs. no alcohol consumption
eGFR
RR [95% CI]
Weight
eGFR < Median
Kansai Healthcare 2016, 23.1–46.0 g/day, Men 84.7 0.86 [0.75, 0.98] 5.42
Kimura 2018, 20–39 g/day, Men 75.0 0.82 [0.75, 0.90] 8.66
Kimura 2018, 20–39 g/day, Women 76.0 0.93 [0.78, 1.11] 3.57
PROMISE 2021, 20–39 g/day, Men & Woomen 78.0 0.73 [0.56, 0.95] 1.86
Tanaka 2022, 23–46 g/day, Men 85.9 0.99 [0.88, 1.12] 6.24
Tanaka 2022, 23–46 g/day, Women 75.6 0.93 [0.67, 1.30] 1.18
Heterogeneity: τ² = 0.00, I² = 41.22%, H² = 1.70 0.88 [0.81, 0.95]
eGFR ≥ Median
PREVEND 2015, 70–210 g/week, Men & Women 97.3 0.82 [0.65, 1.04] 2.13
PREVEND 2015, >120 g/week, Men & Women 97.3 0.58 [0.38, 0.88] 0.75
Park 2019, 10–19.9 g/day, Men 91.7 0.99 [0.98, 1.00] 17.74
Park 2019, 20–39.9 g/day, Men 91.7 1.04 [1.03, 1.05] 17.74
Park 2019, 10–19.9 g/day, Women 92.6 1.06 [1.04, 1.08] 17.04
Park 2019, 20–39.9 g/day, Women 92.6 1.07 [1.04, 1.11] 16.14
Heterogeneity: τ² = 0.00, I² = 93.91%, H² = 16.41 1.03 [0.99, 1.07]
Overall 0.98 [0.95, 1.02]
Heterogeneity: τ² = 0.00, I² = 90.78%, H² = 10.84
Test of group differences: Qb(1) = 11.74, p = 0.00
0.6
0.8
1.0
1.2
RR of proteinuria

Figure S4f

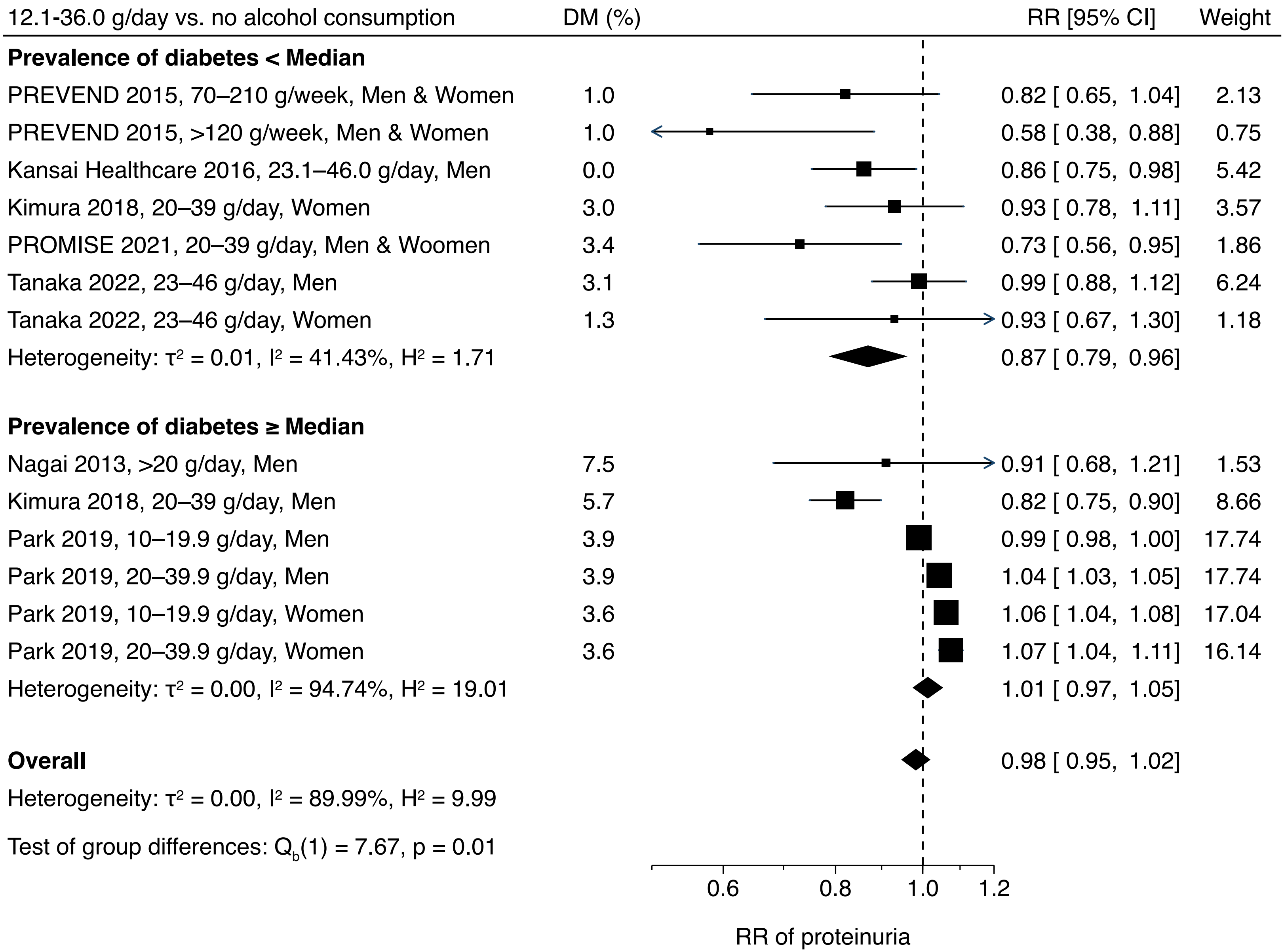

12.1-36.0 g/day vs. no alcohol consumption
DM (%)
RR [95% CI]
Weight
Prevalence of diabetes < Median
PREVEND 2015, 70–210 g/week, Men & Women 1.0 0.82 [0.65, 1.04] 2.13
PREVEND 2015, >120 g/week, Men & Women 1.0 0.58 [0.38, 0.88] 0.75
Kansai Healthcare 2016, 23.1–46.0 g/day, Men 0.0 0.86 [0.75, 0.98] 5.42
Kimura 2018, 20–39 g/day, Women 3.0 0.93 [0.78, 1.11] 3.57
PROMISE 2021, 20–39 g/day, Men & Woomen 3.4 0.73 [0.56, 0.95] 1.86
Tanaka 2022, 23–46 g/day, Men 3.1 0.99 [0.88, 1.12] 6.24
Tanaka 2022, 23–46 g/day, Women 1.3 0.93 [0.67, 1.30] 1.18
Heterogeneity: τ^2 = 0.01, I^2 = 41.43%, H^2 = 1.71 0.87 [0.79, 0.96]
Prevalence of diabetes ≥ Median
Nagai 2013, >20 g/day, Men 7.5 0.91 [0.68, 1.21] 1.53
Kimura 2018, 20–39 g/day, Men 5.7 0.82 [0.75, 0.90] 8.66
Park 2019, 10–19.9 g/day, Men 3.9 0.99 [0.98, 1.00] 17.74
Park 2019, 20–39.9 g/day, Men 3.9 1.04 [1.03, 1.05] 17.74
Park 2019, 10–19.9 g/day, Women 3.6 1.06 [1.04, 1.08] 17.04
Park 2019, 20–39.9 g/day, Women 3.6 1.07 [1.04, 1.11] 16.14
Heterogeneity: τ^2 = 0.00, I^2 = 94.74%, H^2 = 19.01 1.01 [0.97, 1.05]
Overall 0.98 [0.95, 1.02]
Heterogeneity: τ^2 = 0.00, I^2 = 89.99%, H^2 = 9.99
Test of group differences: $Q_b(1)$ = 7.67, p = 0.01
0.6
0.8
1.0
1.2
RR of proteinuria

Figure S4g

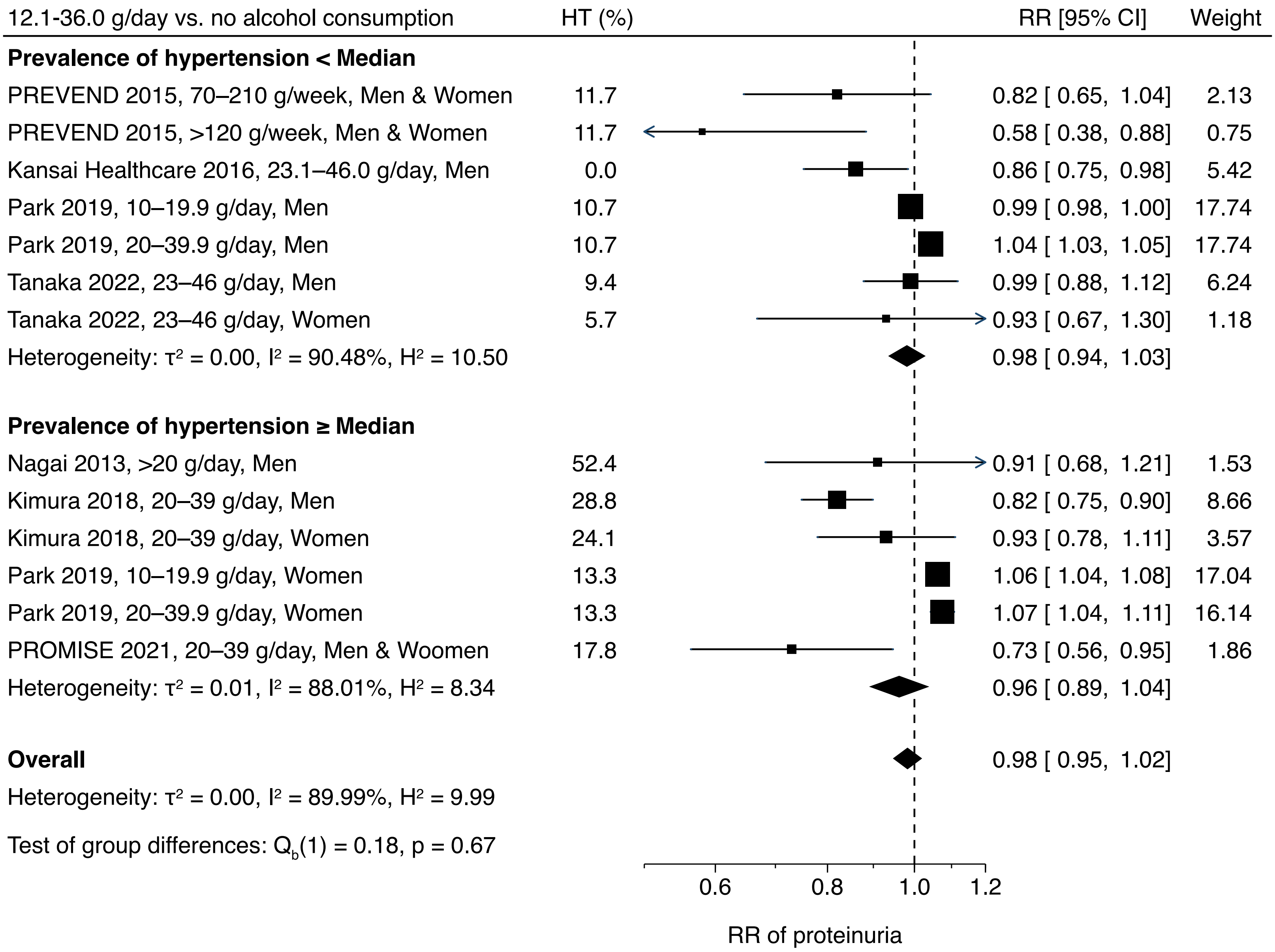
12.1-36.0 g/day vs. no alcohol consumption
HT (%)
RR [95% CI]
Weight
Prevalence of hypertension < Median
PREVEND 2015, 70–210 g/week, Men & Women 11.7 0.82 [0.65, 1.04] 2.13
PREVEND 2015, >120 g/week, Men & Women 11.7 0.58 [0.38, 0.88] 0.75
Kansai Healthcare 2016, 23.1–46.0 g/day, Men 0.0 0.86 [0.75, 0.98] 5.42
Park 2019, 10–19.9 g/day, Men 10.7 0.99 [0.98, 1.00] 17.74
Park 2019, 20–39.9 g/day, Men 10.7 1.04 [1.03, 1.05] 17.74
Tanaka 2022, 23–46 g/day, Men 9.4 0.99 [0.88, 1.12] 6.24
Tanaka 2022, 23–46 g/day, Women 5.7 0.93 [0.67, 1.30] 1.18
Heterogeneity: τ² = 0.00, I² = 90.48%, H² = 10.50 0.98 [0.94, 1.03]
Prevalence of hypertension ≥ Median
Nagai 2013, >20 g/day, Men 52.4 0.91 [0.68, 1.21] 1.53
Kimura 2018, 20–39 g/day, Men 28.8 0.82 [0.75, 0.90] 8.66
Kimura 2018, 20–39 g/day, Women 24.1 0.93 [0.78, 1.11] 3.57
Park 2019, 10–19.9 g/day, Women 13.3 1.06 [1.04, 1.08] 17.04
Park 2019, 20–39.9 g/day, Women 13.3 1.07 [1.04, 1.11] 16.14
PROMISE 2021, 20–39 g/day, Men & Woomen 17.8 0.73 [0.56, 0.95] 1.86
Heterogeneity: τ² = 0.01, I² = 88.01%, H² = 8.34 0.96 [0.89, 1.04]
Overall 0.98 [0.95, 1.02]
Heterogeneity: τ² = 0.00, I² = 89.99%, H² = 9.99
Test of group differences: Q_b(1) = 0.18, p = 0.67
0.6
0.8
1.0
1.2
RR of proteinuria

Figure S4h

12.1-36.0 g/day vs. no alcohol consumption	Follow-up	RR [95% CI]	Weight
**Follow-up duration < Median**			
Nagai 2013, >20 g/day, Men	4.0	0.91 [0.68, 1.21]	1.53
Kimura 2018, 20–39 g/day, Men	1.8	0.82 [0.75, 0.90]	8.66
Kimura 2018, 20–39 g/day, Women	1.7	0.93 [0.78, 1.11]	3.57
PROMISE 2021, 20–39 g/day, Men & Woomen	5.0	0.73 [0.56, 0.95]	1.86
Tanaka 2022, 23–46 g/day, Men	4.0	0.99 [0.88, 1.12]	6.24
Tanaka 2022, 23–46 g/day, Women	4.0	0.93 [0.67, 1.30]	1.18
Heterogeneity: $\tau^2 = 0.01$, $I^2 = 41.20\%$, $H^2 = 1.70$		0.88 [0.81, 0.97]	
**Follow-up duration ≥ Median**			
PREVEND 2015, 70–210 g/week, Men & Women	10.2	0.82 [0.65, 1.04]	2.13
PREVEND 2015, >120 g/week, Men & Women	10.2	0.58 [0.38, 0.88]	0.75
Kansai Healthcare 2016, 23.1–46.0 g/day, Men	8.0	0.86 [0.75, 0.98]	5.42
Park 2019, 10–19.9 g/day, Men	6.4	0.99 [0.98, 1.00]	17.74
Park 2019, 20–39.9 g/day, Men	6.4	1.04 [1.03, 1.05]	17.74
Park 2019, 10–19.9 g/day, Women	6.4	1.06 [1.04, 1.08]	17.04
Park 2019, 20–39.9 g/day, Women	6.4	1.07 [1.04, 1.11]	16.14
Heterogeneity: $\tau^2 = 0.00$, $I^2 = 93.23\%$, $H^2 = 14.76$		1.02 [0.98, 1.06]	
**Overall**		0.98 [0.95, 1.02]	
Heterogeneity: $\tau^2 = 0.00$, $I^2 = 89.99\%$, $H^2 = 9.99$			
Test of group differences: $Q_b(1) = 7.35$, $p = 0.01$			

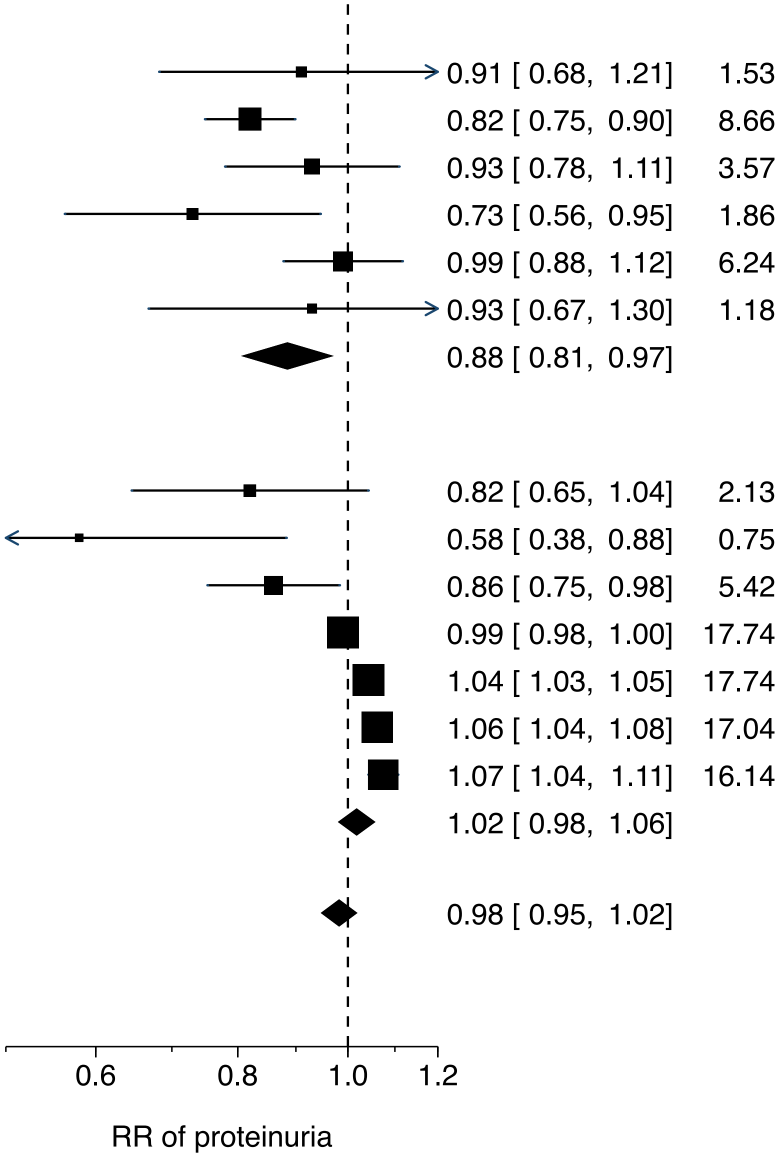

Figure S4i

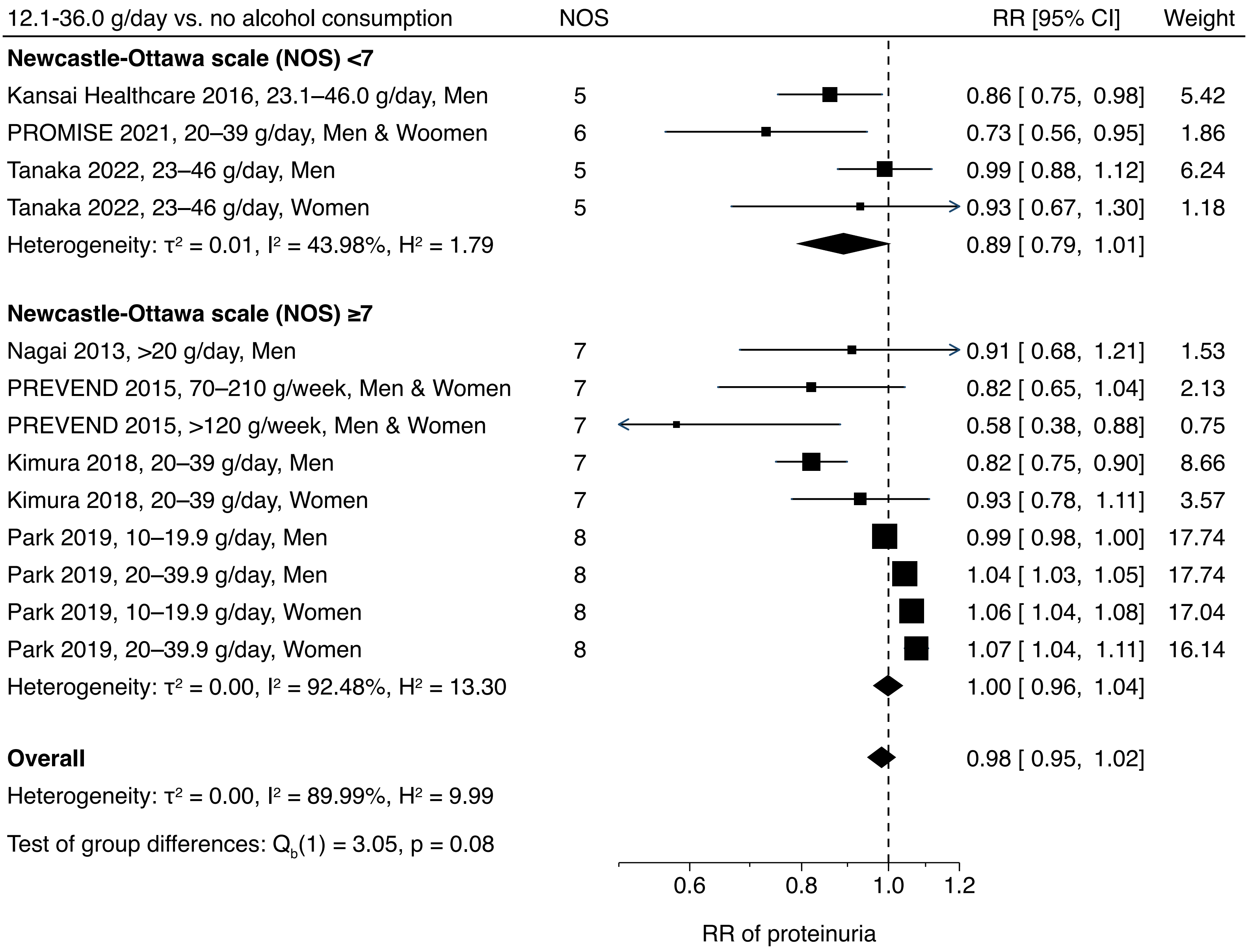
12.1-36.0 g/day vs. no alcohol consumption
NOS
RR [95% CI]
Weight
Newcastle-Ottawa scale (NOS) <7
Kansai Healthcare 2016, 23.1–46.0 g/day, Men 5 0.86 [0.75, 0.98] 5.42
PROMISE 2021, 20–39 g/day, Men & Woomen 6 0.73 [0.56, 0.95] 1.86
Tanaka 2022, 23–46 g/day, Men 5 0.99 [0.88, 1.12] 6.24
Tanaka 2022, 23–46 g/day, Women 5 0.93 [0.67, 1.30] 1.18
Heterogeneity: τ² = 0.01, I² = 43.98%, H² = 1.79 0.89 [0.79, 1.01]
Newcastle-Ottawa scale (NOS) ≥7
Nagai 2013, >20 g/day, Men 7 0.91 [0.68, 1.21] 1.53
PREVEND 2015, 70–210 g/week, Men & Women 7 0.82 [0.65, 1.04] 2.13
PREVEND 2015, >120 g/week, Men & Women 7 0.58 [0.38, 0.88] 0.75
Kimura 2018, 20–39 g/day, Men 7 0.82 [0.75, 0.90] 8.66
Kimura 2018, 20–39 g/day, Women 7 0.93 [0.78, 1.11] 3.57
Park 2019, 10–19.9 g/day, Men 8 0.99 [0.98, 1.00] 17.74
Park 2019, 20–39.9 g/day, Men 8 1.04 [1.03, 1.05] 17.74
Park 2019, 10–19.9 g/day, Women 8 1.06 [1.04, 1.08] 17.04
Park 2019, 20–39.9 g/day, Women 8 1.07 [1.04, 1.11] 16.14
Heterogeneity: τ² = 0.00, I² = 92.48%, H² = 13.30 1.00 [0.96, 1.04]
Overall 0.98 [0.95, 1.02]
Heterogeneity: τ² = 0.00, I² = 89.99%, H² = 9.99
Test of group differences: Qb(1) = 3.05, p = 0.08
0.6
0.8
1.0
1.2
RR of proteinuria

Figure S4j

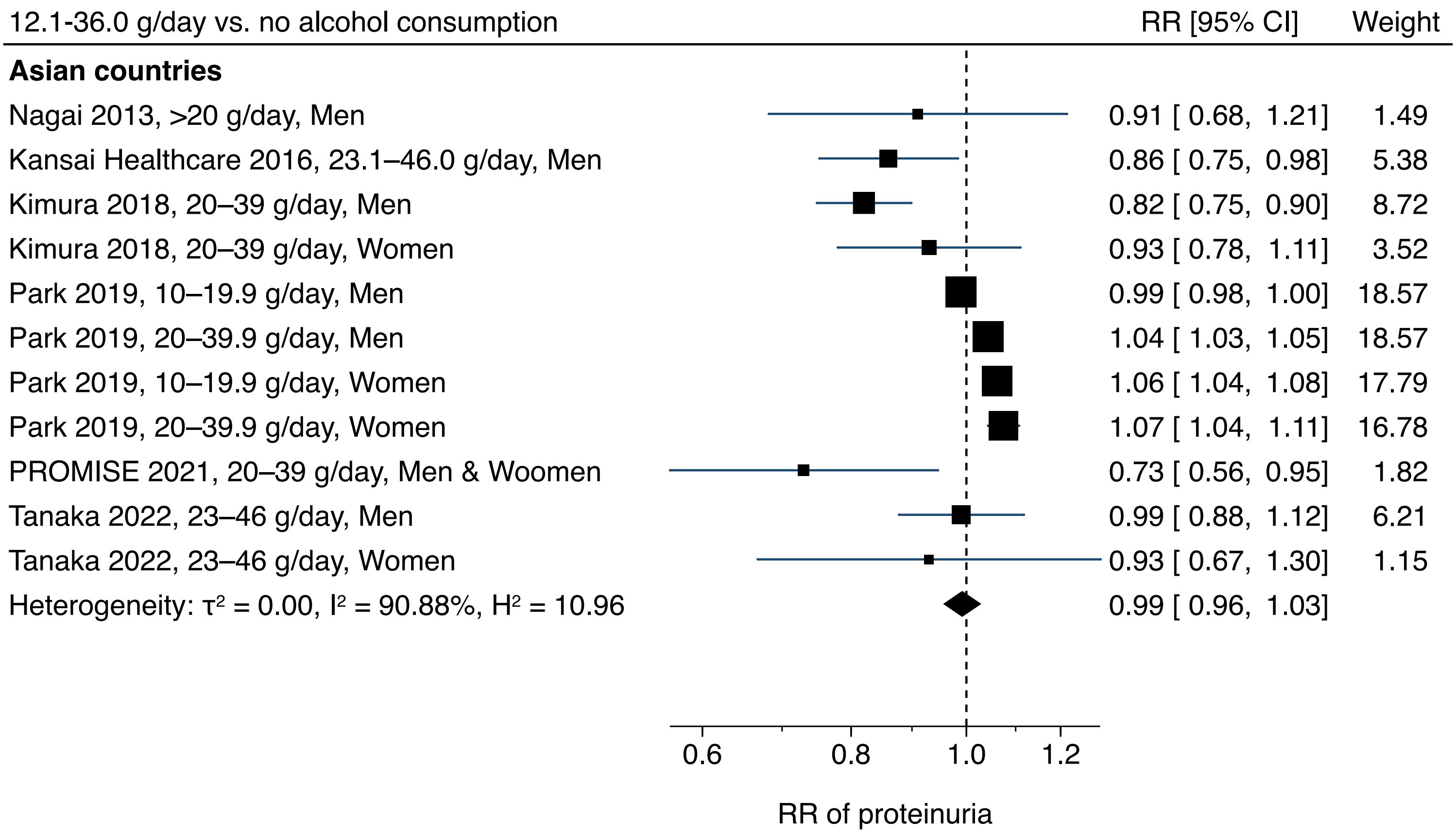
12.1-36.0 g/day vs. no alcohol consumption
RR [95% CI]
Weight
Asian countries
Nagai 2013, >20 g/day, Men
0.91 [0.68, 1.21] 1.49
Kansai Healthcare 2016, 23.1–46.0 g/day, Men
0.86 [0.75, 0.98] 5.38
Kimura 2018, 20–39 g/day, Men
0.82 [0.75, 0.90] 8.72
Kimura 2018, 20–39 g/day, Women
0.93 [0.78, 1.11] 3.52
Park 2019, 10–19.9 g/day, Men
0.99 [0.98, 1.00] 18.57
Park 2019, 20–39.9 g/day, Men
1.04 [1.03, 1.05] 18.57
Park 2019, 10–19.9 g/day, Women
1.06 [1.04, 1.08] 17.79
Park 2019, 20–39.9 g/day, Women
1.07 [1.04, 1.11] 16.78
PROMISE 2021, 20–39 g/day, Men & Woomen
0.73 [0.56, 0.95] 1.82
Tanaka 2022, 23–46 g/day, Men
0.99 [0.88, 1.12] 6.21
Tanaka 2022, 23–46 g/day, Women
0.93 [0.67, 1.30] 1.15
Heterogeneity: τ² = 0.00, I² = 90.88%, H² = 10.96
0.99 [0.96, 1.03]
0.6
0.8
1.0
1.2
RR of proteinuria

Figure S5a

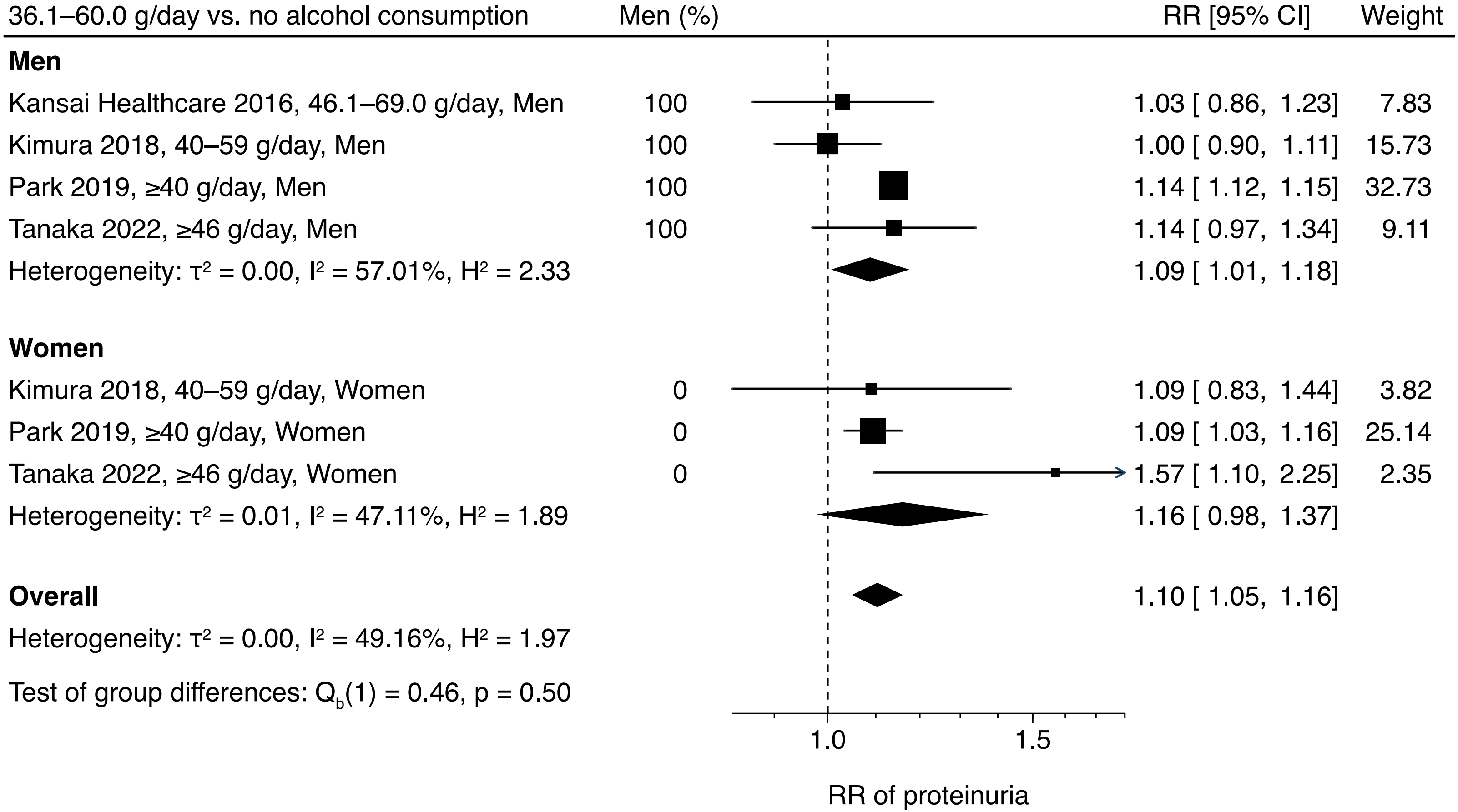
36.1–60.0 g/day vs. no alcohol consumption
Men (%)
RR [95% CI]
Weight
Men
Kansai Healthcare 2016, 46.1–69.0 g/day, Men
100
1.03 [0.86, 1.23]
7.83
Kimura 2018, 40–59 g/day, Men
100
1.00 [0.90, 1.11]
15.73
Park 2019, ≥40 g/day, Men
100
1.14 [1.12, 1.15]
32.73
Tanaka 2022, ≥46 g/day, Men
100
1.14 [0.97, 1.34]
9.11
Heterogeneity: τ² = 0.00, I² = 57.01%, H² = 2.33
1.09 [1.01, 1.18]
Women
Kimura 2018, 40–59 g/day, Women
0
1.09 [0.83, 1.44]
3.82
Park 2019, ≥40 g/day, Women
0
1.09 [1.03, 1.16]
25.14
Tanaka 2022, ≥46 g/day, Women
0
1.57 [1.10, 2.25]
2.35
Heterogeneity: τ² = 0.01, I² = 47.11%, H² = 1.89
1.16 [0.98, 1.37]
Overall
1.10 [1.05, 1.16]
Heterogeneity: τ² = 0.00, I² = 49.16%, H² = 1.97
Test of group differences: Qb(1) = 0.46, p = 0.50
1.0
1.5
RR of proteinuria

Figure S5b

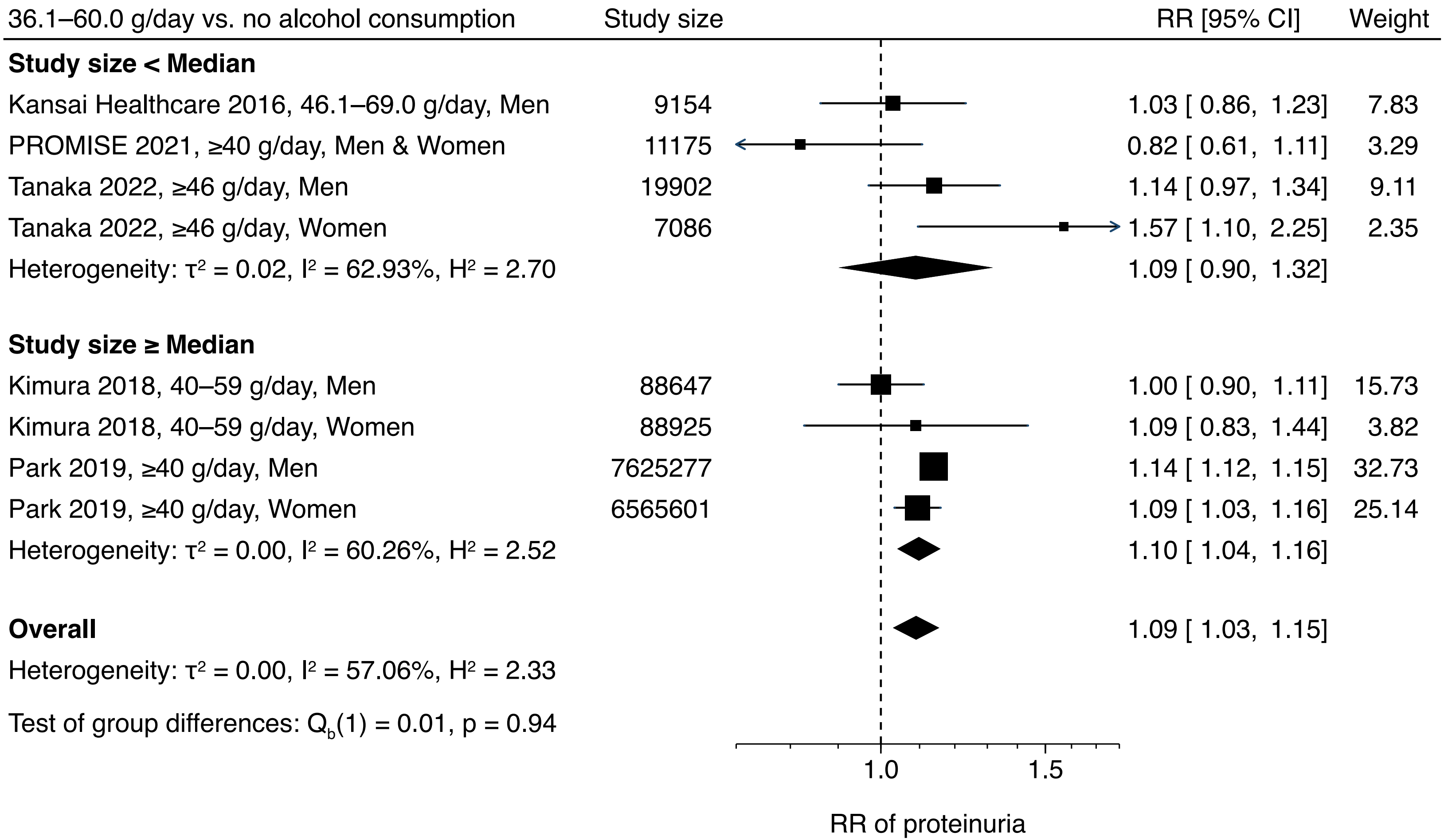

36.1–60.0 g/day vs. no alcohol consumption
Study size
RR [95% CI]
Weight
Study size < Median
Kansai Healthcare 2016, 46.1–69.0 g/day, Men 9154 1.03 [0.86, 1.23] 7.83
PROMISE 2021, ≥40 g/day, Men & Women 11175 0.82 [0.61, 1.11] 3.29
Tanaka 2022, ≥46 g/day, Men 19902 1.14 [0.97, 1.34] 9.11
Tanaka 2022, ≥46 g/day, Women 7086 1.57 [1.10, 2.25] 2.35
Heterogeneity: τ² = 0.02, I² = 62.93%, H² = 2.70 1.09 [0.90, 1.32]
Study size ≥ Median
Kimura 2018, 40–59 g/day, Men 88647 1.00 [0.90, 1.11] 15.73
Kimura 2018, 40–59 g/day, Women 88925 1.09 [0.83, 1.44] 3.82
Park 2019, ≥40 g/day, Men 7625277 1.14 [1.12, 1.15] 32.73
Park 2019, ≥40 g/day, Women 6565601 1.09 [1.03, 1.16] 25.14
Heterogeneity: τ² = 0.00, I² = 60.26%, H² = 2.52 1.10 [1.04, 1.16]
Overall 1.09 [1.03, 1.15]
Heterogeneity: τ² = 0.00, I² = 57.06%, H² = 2.33
Test of group differences: Qb(1) = 0.01, p = 0.94
1.0
1.5
RR of proteinuria

Figure S5c

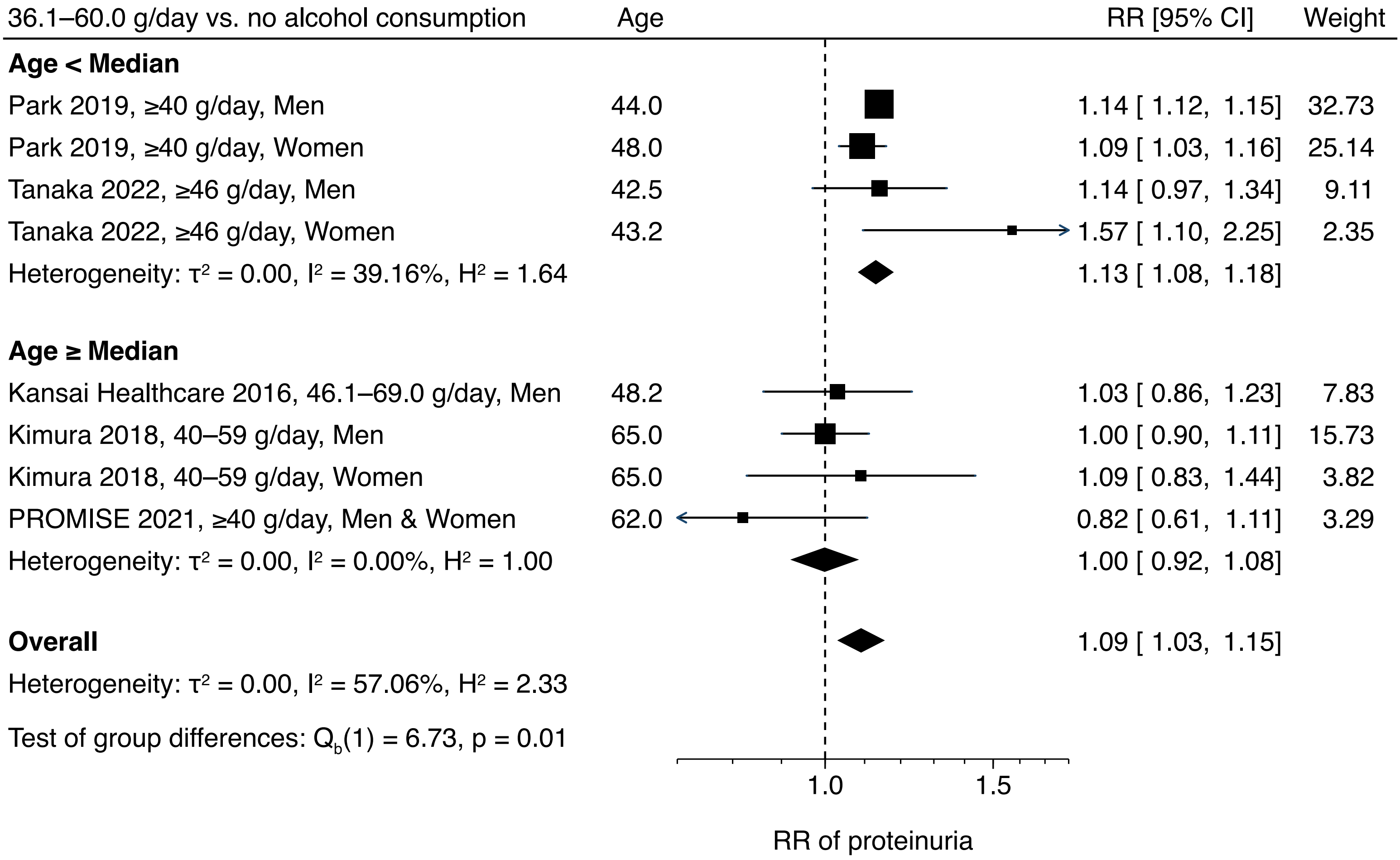

36.1–60.0 g/day vs. no alcohol consumption
Age
RR [95% CI]
Weight
Age < Median
Park 2019, ≥40 g/day, Men 44.0 1.14 [1.12, 1.15] 32.73
Park 2019, ≥40 g/day, Women 48.0 1.09 [1.03, 1.16] 25.14
Tanaka 2022, ≥46 g/day, Men 42.5 1.14 [0.97, 1.34] 9.11
Tanaka 2022, ≥46 g/day, Women 43.2 1.57 [1.10, 2.25] 2.35
Heterogeneity: τ² = 0.00, I² = 39.16%, H² = 1.64 1.13 [1.08, 1.18]
Age ≥ Median
Kansai Healthcare 2016, 46.1–69.0 g/day, Men 48.2 1.03 [0.86, 1.23] 7.83
Kimura 2018, 40–59 g/day, Men 65.0 1.00 [0.90, 1.11] 15.73
Kimura 2018, 40–59 g/day, Women 65.0 1.09 [0.83, 1.44] 3.82
PROMISE 2021, ≥40 g/day, Men & Women 62.0 0.82 [0.61, 1.11] 3.29
Heterogeneity: τ² = 0.00, I² = 0.00%, H² = 1.00 1.00 [0.92, 1.08]
Overall 1.09 [1.03, 1.15]
Heterogeneity: τ² = 0.00, I² = 57.06%, H² = 2.33
Test of group differences: Q_b(1) = 6.73, p = 0.01
1.0
1.5
RR of proteinuria

Figure S5d

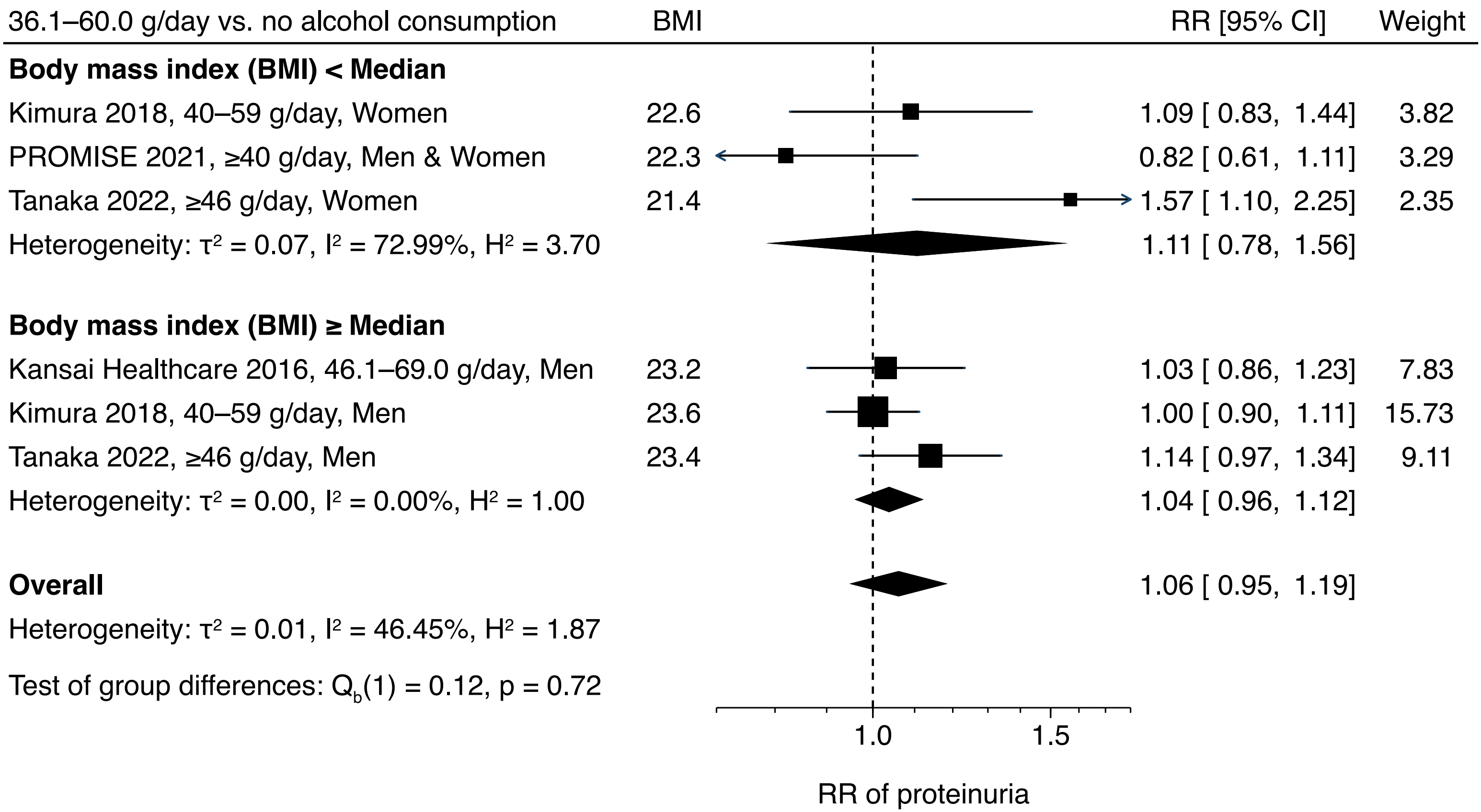
36.1–60.0 g/day vs. no alcohol consumption
BMI
RR [95% CI]
Weight
Body mass index (BMI) < Median
Kimura 2018, 40–59 g/day, Women
22.6
1.09 [0.83, 1.44]
3.82
PROMISE 2021, ≥40 g/day, Men & Women
22.3
0.82 [0.61, 1.11]
3.29
Tanaka 2022, ≥46 g/day, Women
21.4
1.57 [1.10, 2.25]
2.35
Heterogeneity: τ² = 0.07, I² = 72.99%, H² = 3.70
1.11 [0.78, 1.56]
Body mass index (BMI) ≥ Median
Kansai Healthcare 2016, 46.1–69.0 g/day, Men
23.2
1.03 [0.86, 1.23]
7.83
Kimura 2018, 40–59 g/day, Men
23.6
1.00 [0.90, 1.11]
15.73
Tanaka 2022, ≥46 g/day, Men
23.4
1.14 [0.97, 1.34]
9.11
Heterogeneity: τ² = 0.00, I² = 0.00%, H² = 1.00
1.04 [0.96, 1.12]
Overall
1.06 [0.95, 1.19]
Heterogeneity: τ² = 0.01, I² = 46.45%, H² = 1.87
Test of group differences: Q_b(1) = 0.12, p = 0.72
1.0
1.5
RR of proteinuria

Figure S5e

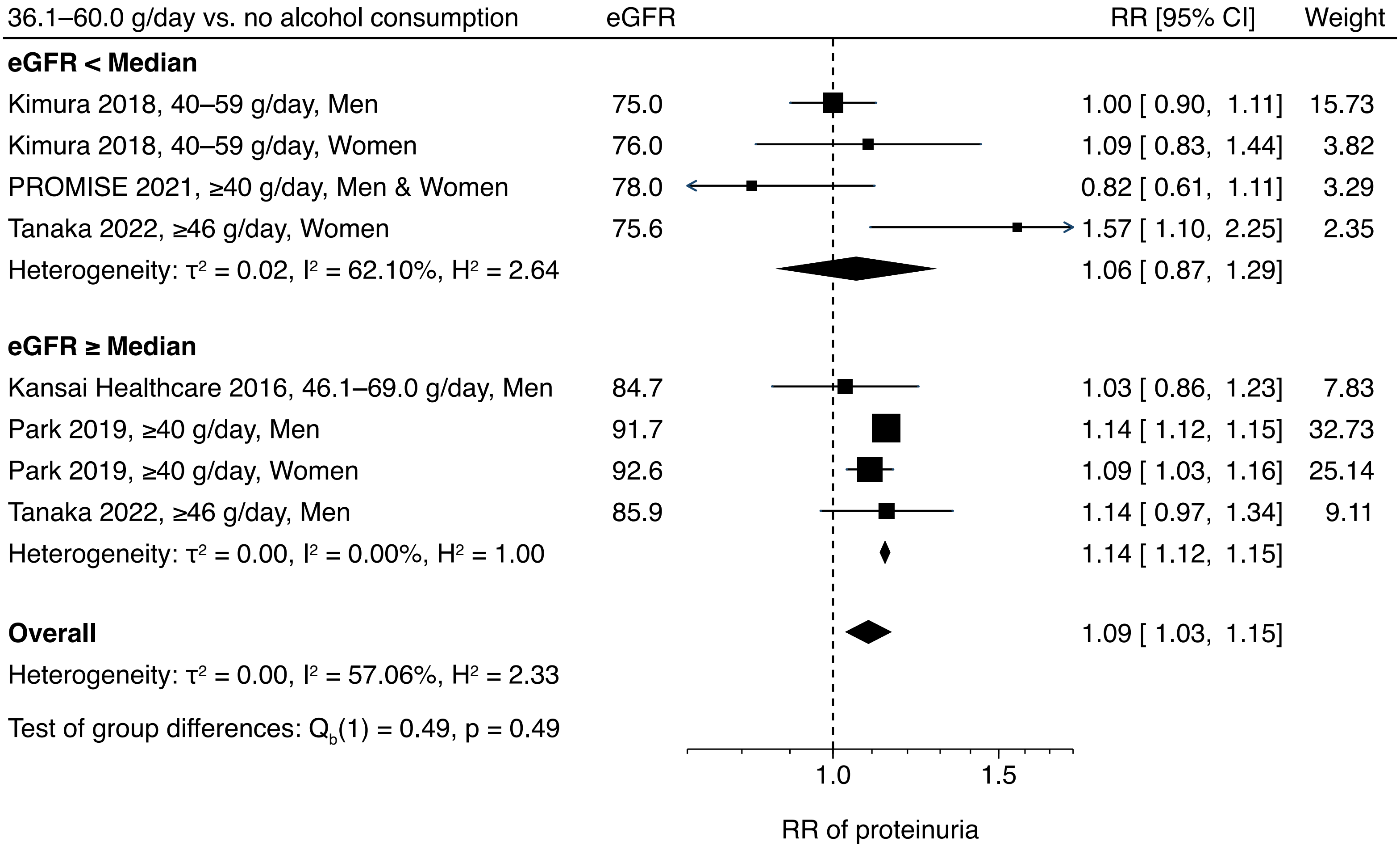
36.1–60.0 g/day vs. no alcohol consumption
eGFR
RR [95% CI]
Weight
eGFR < Median
Kimura 2018, 40–59 g/day, Men 75.0 1.00 [0.90, 1.11] 15.73
Kimura 2018, 40–59 g/day, Women 76.0 1.09 [0.83, 1.44] 3.82
PROMISE 2021, ≥40 g/day, Men & Women 78.0 0.82 [0.61, 1.11] 3.29
Tanaka 2022, ≥46 g/day, Women 75.6 1.57 [1.10, 2.25] 2.35
Heterogeneity: τ² = 0.02, I² = 62.10%, H² = 2.64 1.06 [0.87, 1.29]
eGFR ≥ Median
Kansai Healthcare 2016, 46.1–69.0 g/day, Men 84.7 1.03 [0.86, 1.23] 7.83
Park 2019, ≥40 g/day, Men 91.7 1.14 [1.12, 1.15] 32.73
Park 2019, ≥40 g/day, Women 92.6 1.09 [1.03, 1.16] 25.14
Tanaka 2022, ≥46 g/day, Men 85.9 1.14 [0.97, 1.34] 9.11
Heterogeneity: τ² = 0.00, I² = 0.00%, H² = 1.00 1.14 [1.12, 1.15]
Overall 1.09 [1.03, 1.15]
Heterogeneity: τ² = 0.00, I² = 57.06%, H² = 2.33
Test of group differences: Qb(1) = 0.49, p = 0.49
1.0
1.5
RR of proteinuria

Figure S5f

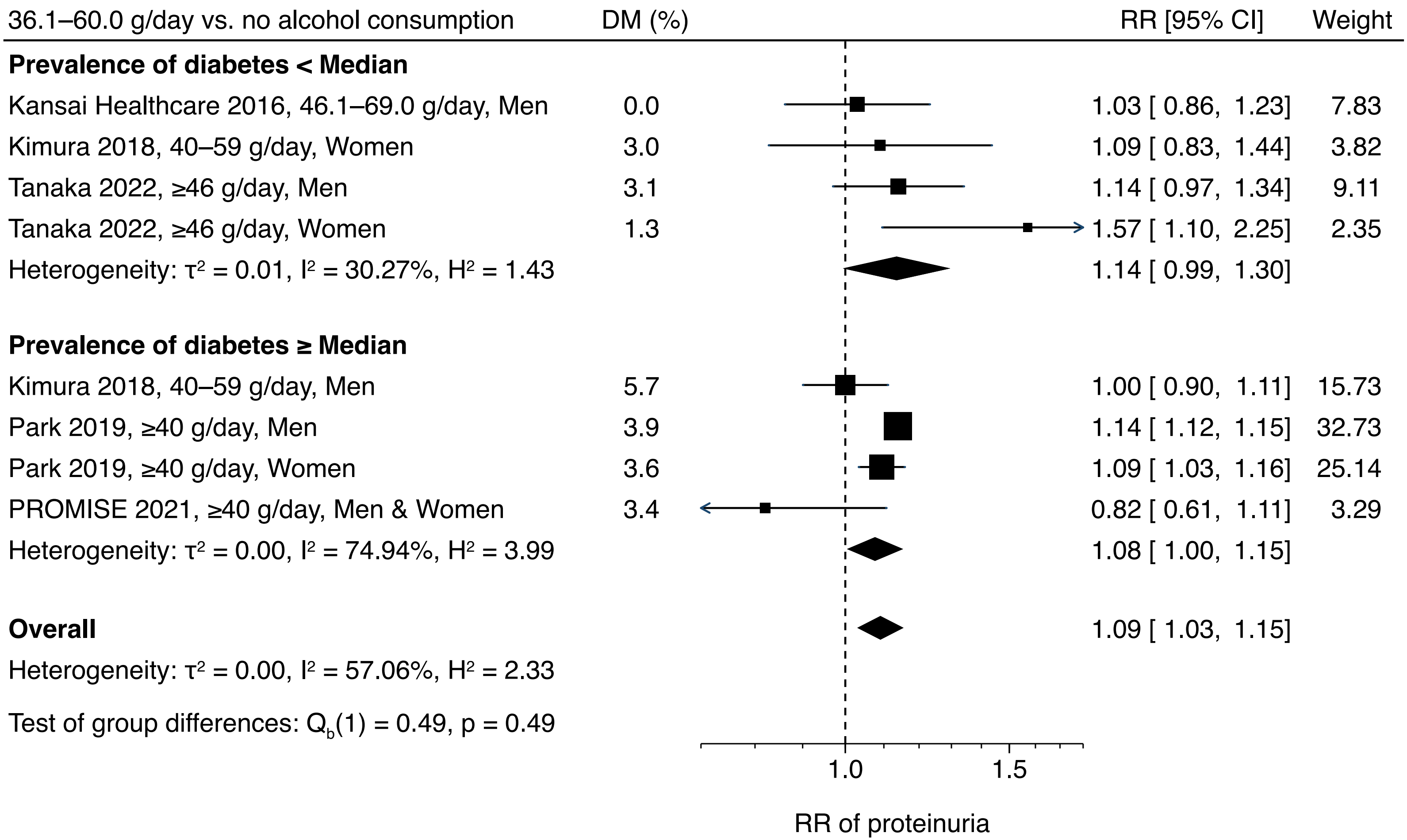
36.1–60.0 g/day vs. no alcohol consumption
DM (%)
RR [95% CI]
Weight
Prevalence of diabetes < Median
Kansai Healthcare 2016, 46.1–69.0 g/day, Men 0.0 1.03 [0.86, 1.23] 7.83
Kimura 2018, 40–59 g/day, Women 3.0 1.09 [0.83, 1.44] 3.82
Tanaka 2022, ≥46 g/day, Men 3.1 1.14 [0.97, 1.34] 9.11
Tanaka 2022, ≥46 g/day, Women 1.3 1.57 [1.10, 2.25] 2.35
Heterogeneity: τ² = 0.01, I² = 30.27%, H² = 1.43 1.14 [0.99, 1.30]
Prevalence of diabetes ≥ Median
Kimura 2018, 40–59 g/day, Men 5.7 1.00 [0.90, 1.11] 15.73
Park 2019, ≥40 g/day, Men 3.9 1.14 [1.12, 1.15] 32.73
Park 2019, ≥40 g/day, Women 3.6 1.09 [1.03, 1.16] 25.14
PROMISE 2021, ≥40 g/day, Men & Women 3.4 0.82 [0.61, 1.11] 3.29
Heterogeneity: τ² = 0.00, I² = 74.94%, H² = 3.99 1.08 [1.00, 1.15]
Overall 1.09 [1.03, 1.15]
Heterogeneity: τ² = 0.00, I² = 57.06%, H² = 2.33
Test of group differences: Qb(1) = 0.49, p = 0.49
1.0
1.5
RR of proteinuria

Figure S5g

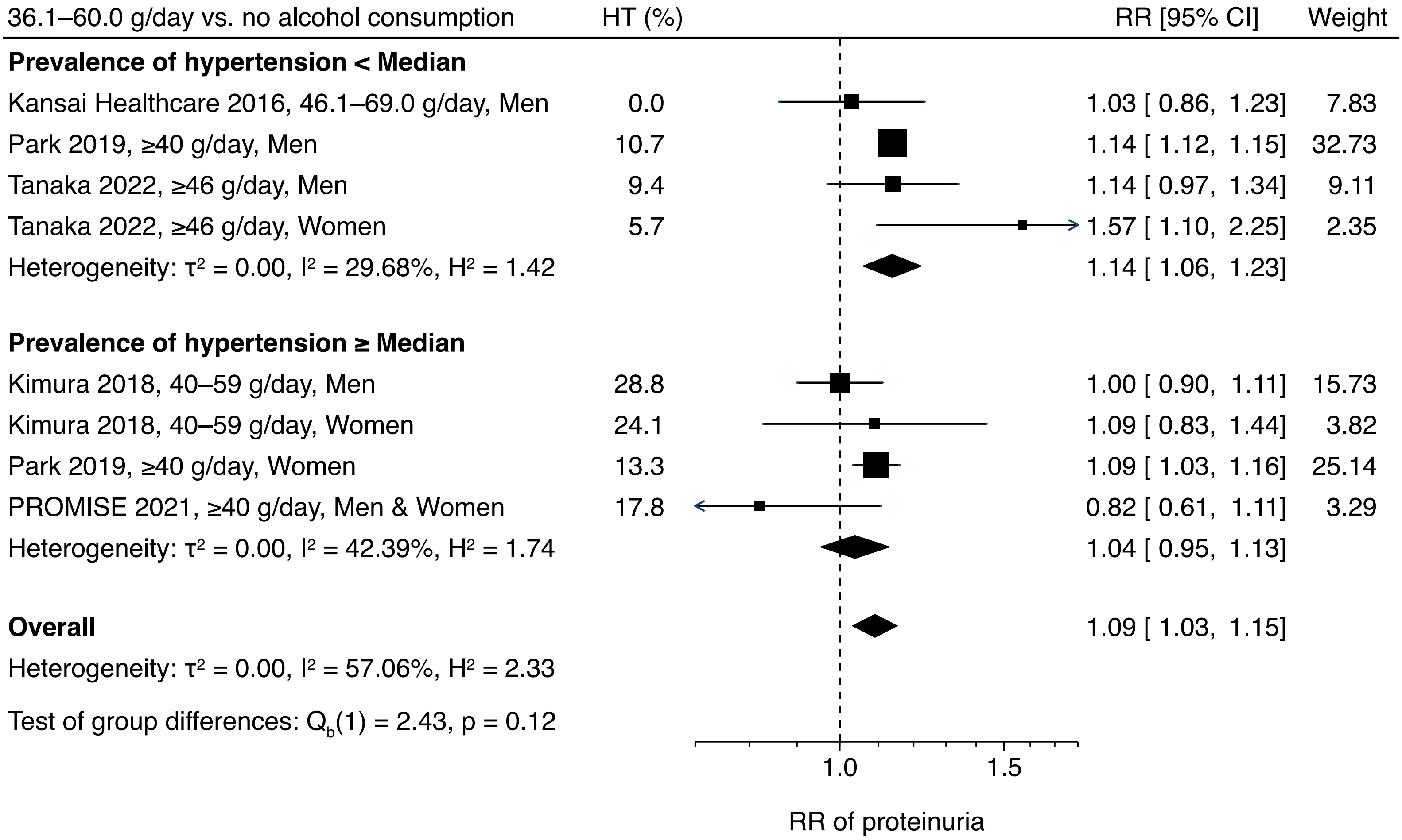
36.1–60.0 g/day vs. no alcohol consumption
HT (%)
RR [95% CI]
Weight
Prevalence of hypertension < Median
Kansai Healthcare 2016, 46.1–69.0 g/day, Men 0.0 1.03 [0.86, 1.23] 7.83
Park 2019, ≥40 g/day, Men 10.7 1.14 [1.12, 1.15] 32.73
Tanaka 2022, ≥46 g/day, Men 9.4 1.14 [0.97, 1.34] 9.11
Tanaka 2022, ≥46 g/day, Women 5.7 1.57 [1.10, 2.25] 2.35
Heterogeneity: τ² = 0.00, I² = 29.68%, H² = 1.42 1.14 [1.06, 1.23]
Prevalence of hypertension ≥ Median
Kimura 2018, 40–59 g/day, Men 28.8 1.00 [0.90, 1.11] 15.73
Kimura 2018, 40–59 g/day, Women 24.1 1.09 [0.83, 1.44] 3.82
Park 2019, ≥40 g/day, Women 13.3 1.09 [1.03, 1.16] 25.14
PROMISE 2021, ≥40 g/day, Men & Women 17.8 0.82 [0.61, 1.11] 3.29
Heterogeneity: τ² = 0.00, I² = 42.39%, H² = 1.74 1.04 [0.95, 1.13]
Overall 1.09 [1.03, 1.15]
Heterogeneity: τ² = 0.00, I² = 57.06%, H² = 2.33
Test of group differences: Qb(1) = 2.43, p = 0.12
1.0
1.5
RR of proteinuria

Figure S5h

36.1–60.0 g/day vs. no alcohol consumption	Follow-up	RR [95% CI]	Weight
**Follow-up duration < Median**			
Kimura 2018, 40–59 g/day, Men	1.8	1.00 [0.90, 1.11]	15.73
Kimura 2018, 40–59 g/day, Women	1.7	1.09 [0.83, 1.44]	3.82
Tanaka 2022, ≥46 g/day, Men	4.0	1.14 [0.97, 1.34]	9.11
Tanaka 2022, ≥46 g/day, Women	4.0	1.57 [1.10, 2.25]	2.35
Heterogeneity: $\tau^2 = 0.01$, $I^2 = 54.25\%$, $H^2 = 2.19$		1.12 [0.97, 1.29]	
**Follow-up duration ≥ Median**			
Kansai Healthcare 2016, 46.1–69.0 g/day, Men	8.0	1.03 [0.86, 1.23]	7.83
Park 2019, ≥40 g/day, Men	6.4	1.14 [1.12, 1.15]	32.73
Park 2019, ≥40 g/day, Women	6.4	1.09 [1.03, 1.16]	25.14
PROMISE 2021, ≥40 g/day, Men & Women	5.0	0.82 [0.61, 1.11]	3.29
Heterogeneity: $\tau^2 = 0.00$, $I^2 = 60.21\%$, $H^2 = 2.51$		1.10 [1.04, 1.17]	
**Overall**		1.09 [1.03, 1.15]	
Heterogeneity: $\tau^2 = 0.00$, $I^2 = 57.06\%$, $H^2 = 2.33$			
Test of group differences: $Q_b(1) = 0.04$, $p = 0.84$			

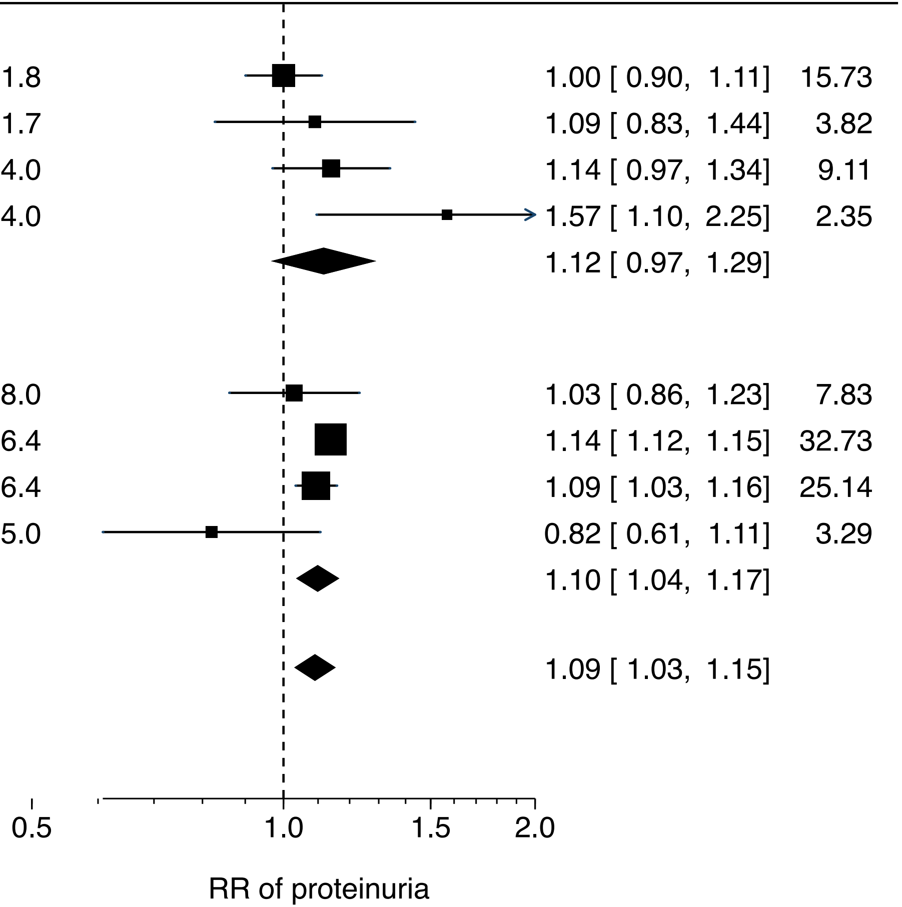

Figure S5i

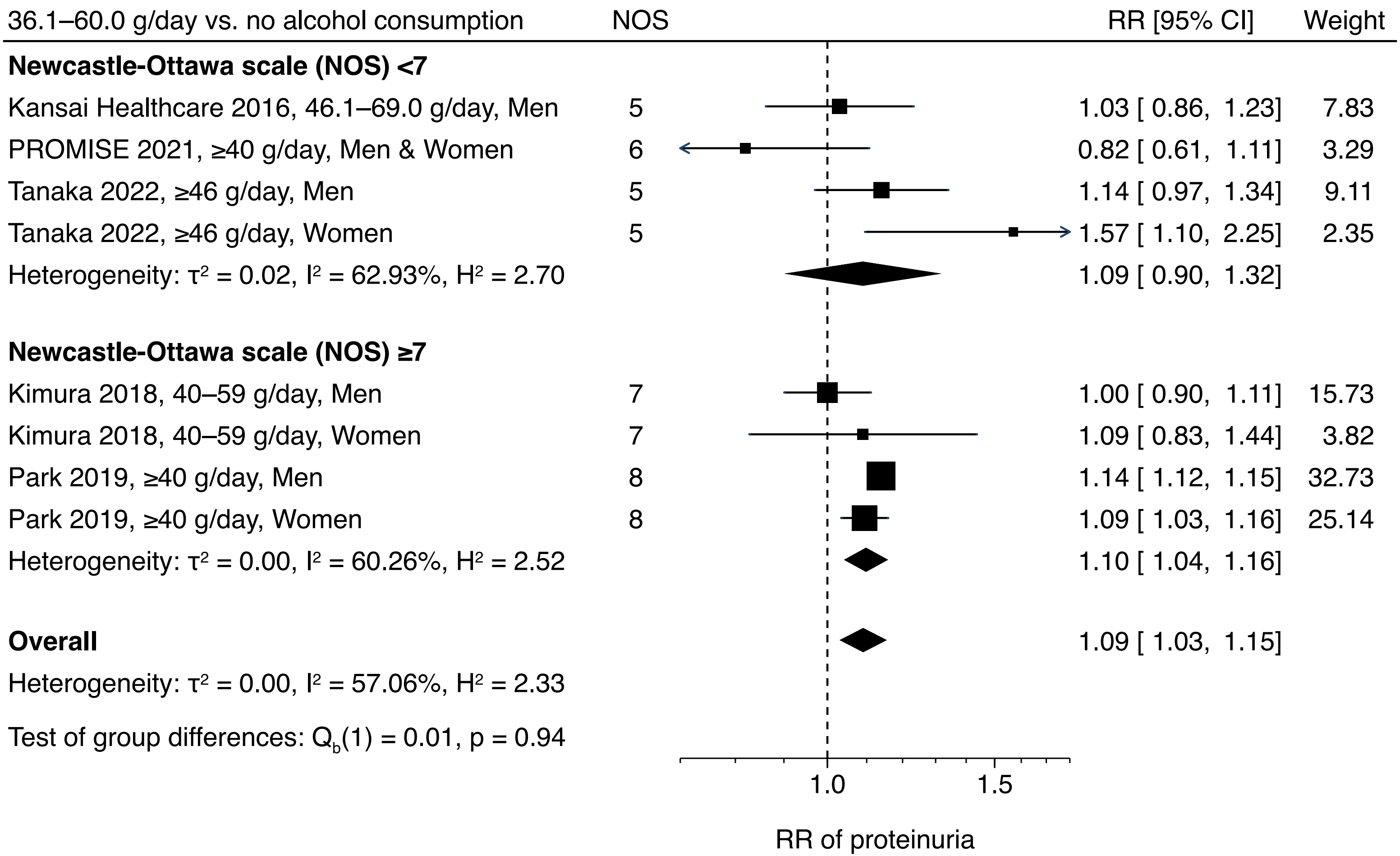
36.1–60.0 g/day vs. no alcohol consumption
NOS
RR [95% CI]
Weight
Newcastle-Ottawa scale (NOS) <7
Kansai Healthcare 2016, 46.1–69.0 g/day, Men 5 1.03 [0.86, 1.23] 7.83
PROMISE 2021, ≥40 g/day, Men & Women 6 0.82 [0.61, 1.11] 3.29
Tanaka 2022, ≥46 g/day, Men 5 1.14 [0.97, 1.34] 9.11
Tanaka 2022, ≥46 g/day, Women 5 1.57 [1.10, 2.25] 2.35
Heterogeneity: τ² = 0.02, I² = 62.93%, H² = 2.70 1.09 [0.90, 1.32]
Newcastle-Ottawa scale (NOS) ≥7
Kimura 2018, 40–59 g/day, Men 7 1.00 [0.90, 1.11] 15.73
Kimura 2018, 40–59 g/day, Women 7 1.09 [0.83, 1.44] 3.82
Park 2019, ≥40 g/day, Men 8 1.14 [1.12, 1.15] 32.73
Park 2019, ≥40 g/day, Women 8 1.09 [1.03, 1.16] 25.14
Heterogeneity: τ² = 0.00, I² = 60.26%, H² = 2.52 1.10 [1.04, 1.16]
Overall 1.09 [1.03, 1.15]
Heterogeneity: τ² = 0.00, I² = 57.06%, H² = 2.33
Test of group differences: Qb(1) = 0.01, p = 0.94
1.0
1.5
RR of proteinuria

Figure S5j

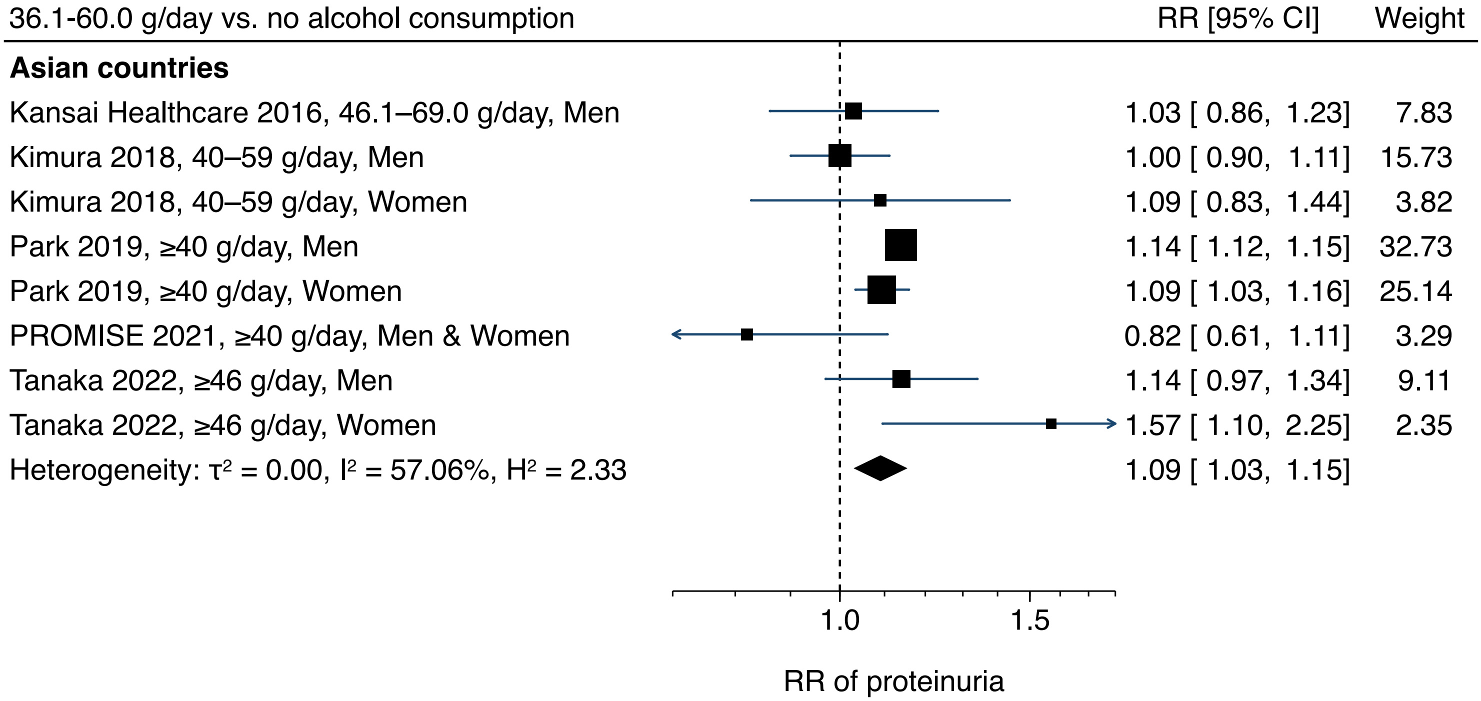

36.1-60.0 g/day vs. no alcohol consumption
RR [95% CI]
Weight
Asian countries
Kansai Healthcare 2016, 46.1–69.0 g/day, Men
1.03 [0.86, 1.23]
7.83
Kimura 2018, 40–59 g/day, Men
1.00 [0.90, 1.11]
15.73
Kimura 2018, 40–59 g/day, Women
1.09 [0.83, 1.44]
3.82
Park 2019, ≥40 g/day, Men
1.14 [1.12, 1.15]
32.73
Park 2019, ≥40 g/day, Women
1.09 [1.03, 1.16]
25.14
PROMISE 2021, ≥40 g/day, Men & Women
0.82 [0.61, 1.11]
3.29
Tanaka 2022, ≥46 g/day, Men
1.14 [0.97, 1.34]
9.11
Tanaka 2022, ≥46 g/day, Women
1.57 [1.10, 2.25]
2.35
Heterogeneity: τ² = 0.00, I² = 57.06%, H² = 2.33
1.09 [1.03, 1.15]
1.0
1.5
RR of proteinuria

Figure S6a

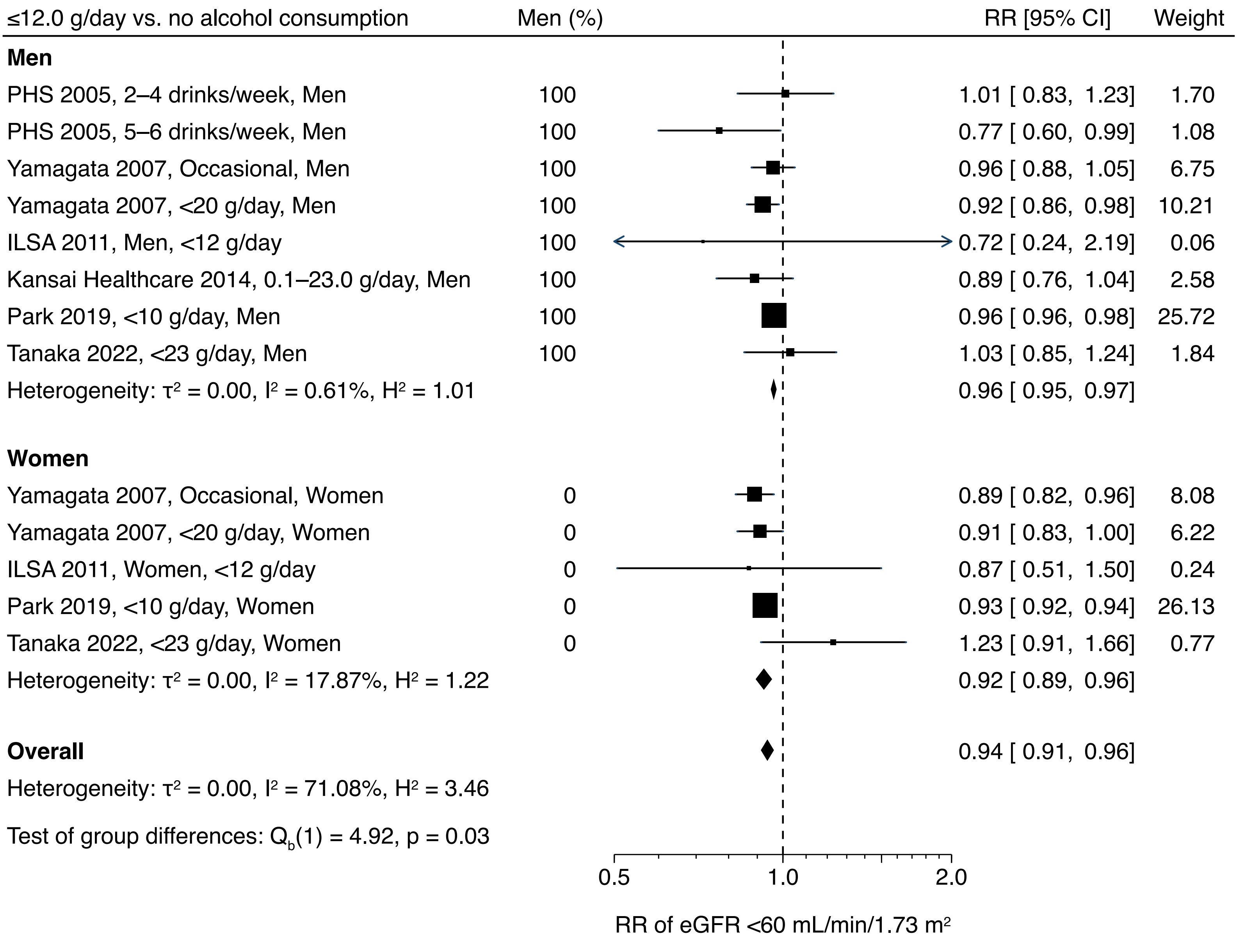

≤12.0 g/day vs. no alcohol consumption
Men (%)
RR [95% CI]
Weight
Men
PHS 2005, 2–4 drinks/week, Men 100 1.01 [0.83, 1.23] 1.70
PHS 2005, 5–6 drinks/week, Men 100 0.77 [0.60, 0.99] 1.08
Yamagata 2007, Occasional, Men 100 0.96 [0.88, 1.05] 6.75
Yamagata 2007, <20 g/day, Men 100 0.92 [0.86, 0.98] 10.21
ILSA 2011, Men, <12 g/day 100 0.72 [0.24, 2.19] 0.06
Kansai Healthcare 2014, 0.1–23.0 g/day, Men 100 0.89 [0.76, 1.04] 2.58
Park 2019, <10 g/day, Men 100 0.96 [0.96, 0.98] 25.72
Tanaka 2022, <23 g/day, Men 100 1.03 [0.85, 1.24] 1.84
Heterogeneity: τ² = 0.00, I² = 0.61%, H² = 1.01 0.96 [0.95, 0.97]
Women
Yamagata 2007, Occasional, Women 0 0.89 [0.82, 0.96] 8.08
Yamagata 2007, <20 g/day, Women 0 0.91 [0.83, 1.00] 6.22
ILSA 2011, Women, <12 g/day 0 0.87 [0.51, 1.50] 0.24
Park 2019, <10 g/day, Women 0 0.93 [0.92, 0.94] 26.13
Tanaka 2022, <23 g/day, Women 0 1.23 [0.91, 1.66] 0.77
Heterogeneity: τ² = 0.00, I² = 17.87%, H² = 1.22 0.92 [0.89, 0.96]
Overall 0.94 [0.91, 0.96]
Heterogeneity: τ² = 0.00, I² = 71.08%, H² = 3.46
Test of group differences: Q_b(1) = 4.92, p = 0.03
0.5
1.0
2.0
RR of eGFR <60 mL/min/1.73 m²

Figure S6b

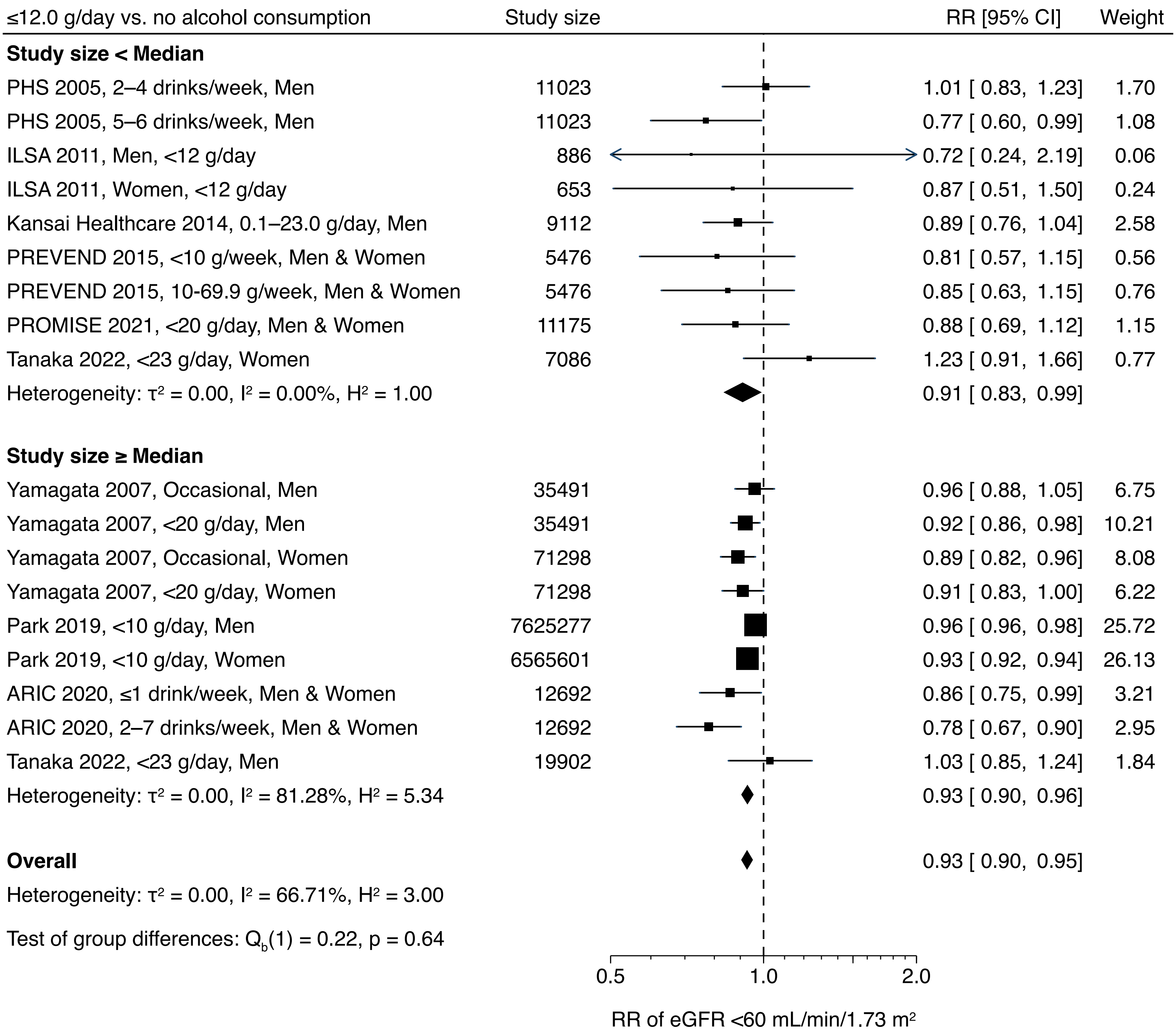
≤12.0 g/day vs. no alcohol consumption
Study size
RR [95% CI]
Weight
Study size < Median
PHS 2005, 2–4 drinks/week, Men | 11023 | 1.01 [0.83, 1.23] | 1.70
PHS 2005, 5–6 drinks/week, Men | 11023 | 0.77 [0.60, 0.99] | 1.08
ILSA 2011, Men, <12 g/day | 886 | 0.72 [0.24, 2.19] | 0.06
ILSA 2011, Women, <12 g/day | 653 | 0.87 [0.51, 1.50] | 0.24
Kansai Healthcare 2014, 0.1–23.0 g/day, Men | 9112 | 0.89 [0.76, 1.04] | 2.58
PREVEND 2015, <10 g/week, Men & Women | 5476 | 0.81 [0.57, 1.15] | 0.56
PREVEND 2015, 10-69.9 g/week, Men & Women | 5476 | 0.85 [0.63, 1.15] | 0.76
PROMISE 2021, <20 g/day, Men & Women | 11175 | 0.88 [0.69, 1.12] | 1.15
Tanaka 2022, <23 g/day, Women | 7086 | 1.23 [0.91, 1.66] | 0.77
Heterogeneity: τ^2 = 0.00, I^2 = 0.00%, H^2 = 1.00 | 0.91 [0.83, 0.99]
Study size ≥ Median
Yamagata 2007, Occasional, Men | 35491 | 0.96 [0.88, 1.05] | 6.75
Yamagata 2007, <20 g/day, Men | 35491 | 0.92 [0.86, 0.98] | 10.21
Yamagata 2007, Occasional, Women | 71298 | 0.89 [0.82, 0.96] | 8.08
Yamagata 2007, <20 g/day, Women | 71298 | 0.91 [0.83, 1.00] | 6.22
Park 2019, <10 g/day, Men | 7625277 | 0.96 [0.96, 0.98] | 25.72
Park 2019, <10 g/day, Women | 6565601 | 0.93 [0.92, 0.94] | 26.13
ARIC 2020, ≤1 drink/week, Men & Women | 12692 | 0.86 [0.75, 0.99] | 3.21
ARIC 2020, 2–7 drinks/week, Men & Women | 12692 | 0.78 [0.67, 0.90] | 2.95
Tanaka 2022, <23 g/day, Men | 19902 | 1.03 [0.85, 1.24] | 1.84
Heterogeneity: τ^2 = 0.00, I^2 = 81.28%, H^2 = 5.34 | 0.93 [0.90, 0.96]
Overall | 0.93 [0.90, 0.95]
Heterogeneity: τ^2 = 0.00, I^2 = 66.71%, H^2 = 3.00
Test of group differences: $Q_b(1)$ = 0.22, p = 0.64
0.5
1.0
2.0
RR of eGFR <60 mL/min/1.73 m^2

Figure S6c

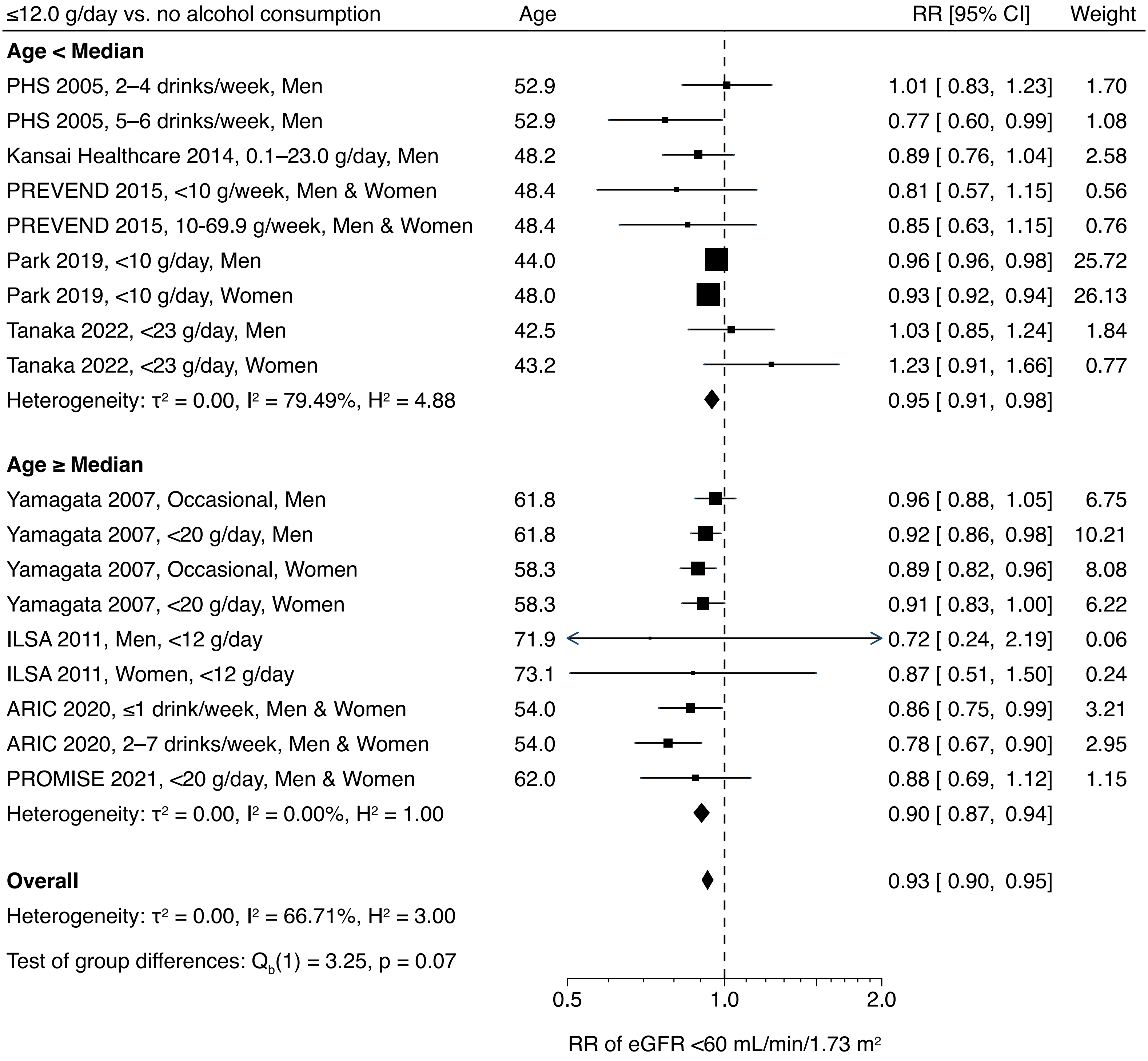
≤12.0 g/day vs. no alcohol consumption
Age
RR [95% CI]
Weight
Age < Median
PHS 2005, 2–4 drinks/week, Men 52.9 1.01 [0.83, 1.23] 1.70
PHS 2005, 5–6 drinks/week, Men 52.9 0.77 [0.60, 0.99] 1.08
Kansai Healthcare 2014, 0.1–23.0 g/day, Men 48.2 0.89 [0.76, 1.04] 2.58
PREVEND 2015, <10 g/week, Men & Women 48.4 0.81 [0.57, 1.15] 0.56
PREVEND 2015, 10-69.9 g/week, Men & Women 48.4 0.85 [0.63, 1.15] 0.76
Park 2019, <10 g/day, Men 44.0 0.96 [0.96, 0.98] 25.72
Park 2019, <10 g/day, Women 48.0 0.93 [0.92, 0.94] 26.13
Tanaka 2022, <23 g/day, Men 42.5 1.03 [0.85, 1.24] 1.84
Tanaka 2022, <23 g/day, Women 43.2 1.23 [0.91, 1.66] 0.77
Heterogeneity: τ^2 = 0.00, I^2 = 79.49%, H^2 = 4.88 0.95 [0.91, 0.98]
Age ≥ Median
Yamagata 2007, Occasional, Men 61.8 0.96 [0.88, 1.05] 6.75
Yamagata 2007, <20 g/day, Men 61.8 0.92 [0.86, 0.98] 10.21
Yamagata 2007, Occasional, Women 58.3 0.89 [0.82, 0.96] 8.08
Yamagata 2007, <20 g/day, Women 58.3 0.91 [0.83, 1.00] 6.22
ILSA 2011, Men, <12 g/day 71.9 0.72 [0.24, 2.19] 0.06
ILSA 2011, Women, <12 g/day 73.1 0.87 [0.51, 1.50] 0.24
ARIC 2020, ≤1 drink/week, Men & Women 54.0 0.86 [0.75, 0.99] 3.21
ARIC 2020, 2–7 drinks/week, Men & Women 54.0 0.78 [0.67, 0.90] 2.95
PROMISE 2021, <20 g/day, Men & Women 62.0 0.88 [0.69, 1.12] 1.15
Heterogeneity: τ^2 = 0.00, I^2 = 0.00%, H^2 = 1.00 0.90 [0.87, 0.94]
Overall 0.93 [0.90, 0.95]
Heterogeneity: τ^2 = 0.00, I^2 = 66.71%, H^2 = 3.00
Test of group differences: $Q_b(1)$ = 3.25, p = 0.07
0.5
1.0
2.0
RR of eGFR <60 mL/min/1.73 m^2

Figure S6d

≤12.0 g/day vs. no alcohol consumption	BMI	RR [95% CI]	Weight
**Body mass index (BMI) < Median**			
Yamagata 2007, Occasional, Men	23.2	0.96 [0.88, 1.05]	6.75
Yamagata 2007, <20 g/day, Men	23.2	0.92 [0.86, 0.98]	10.21
Yamagata 2007, Occasional, Women	23.5	0.89 [0.82, 0.96]	8.08
Yamagata 2007, <20 g/day, Women	23.5	0.91 [0.83, 1.00]	6.22
Kansai Healthcare 2014, 0.1–23.0 g/day, Men	23.2	0.89 [0.76, 1.04]	2.58
PROMISE 2021, <20 g/day, Men & Women	22.3	0.88 [0.69, 1.12]	1.15
Tanaka 2022, <23 g/day, Men	23.4	1.03 [0.85, 1.24]	1.84
Tanaka 2022, <23 g/day, Women	21.4	1.23 [0.91, 1.66]	0.77
Heterogeneity: $\tau^2 = 0.00$, $I^2 = 0.00\%$, $H^2 = 1.00$		0.92 [0.89, 0.96]	
**Body mass index (BMI) ≥ Median**			
PHS 2005, 2–4 drinks/week, Men	24.9	1.01 [0.83, 1.23]	1.70
PHS 2005, 5–6 drinks/week, Men	24.9	0.77 [0.60, 0.99]	1.08
ILSA 2011, Men, <12 g/day	26.5	0.72 [0.24, 2.19]	0.06
ILSA 2011, Women, <12 g/day	27.6	0.87 [0.51, 1.50]	0.24
PREVEND 2015, <10 g/week, Men & Women	25.7	0.81 [0.57, 1.15]	0.56
PREVEND 2015, 10-69.9 g/week, Men & Women	25.7	0.85 [0.63, 1.15]	0.76
ARIC 2020, ≤1 drink/week, Men & Women	27.4	0.86 [0.75, 0.99]	3.21
ARIC 2020, 2–7 drinks/week, Men & Women	27.4	0.78 [0.67, 0.90]	2.95
Heterogeneity: $\tau^2 = 0.00$, $I^2 = 0.00\%$, $H^2 = 1.00$		0.84 [0.78, 0.91]	
**Overall**		0.91 [0.88, 0.94]	
Heterogeneity: $\tau^2 = 0.00$, $I^2 = 6.91\%$, $H^2 = 1.07$			

Test of group differences: $Q_b(1) = 4.13$, $p = 0.04$

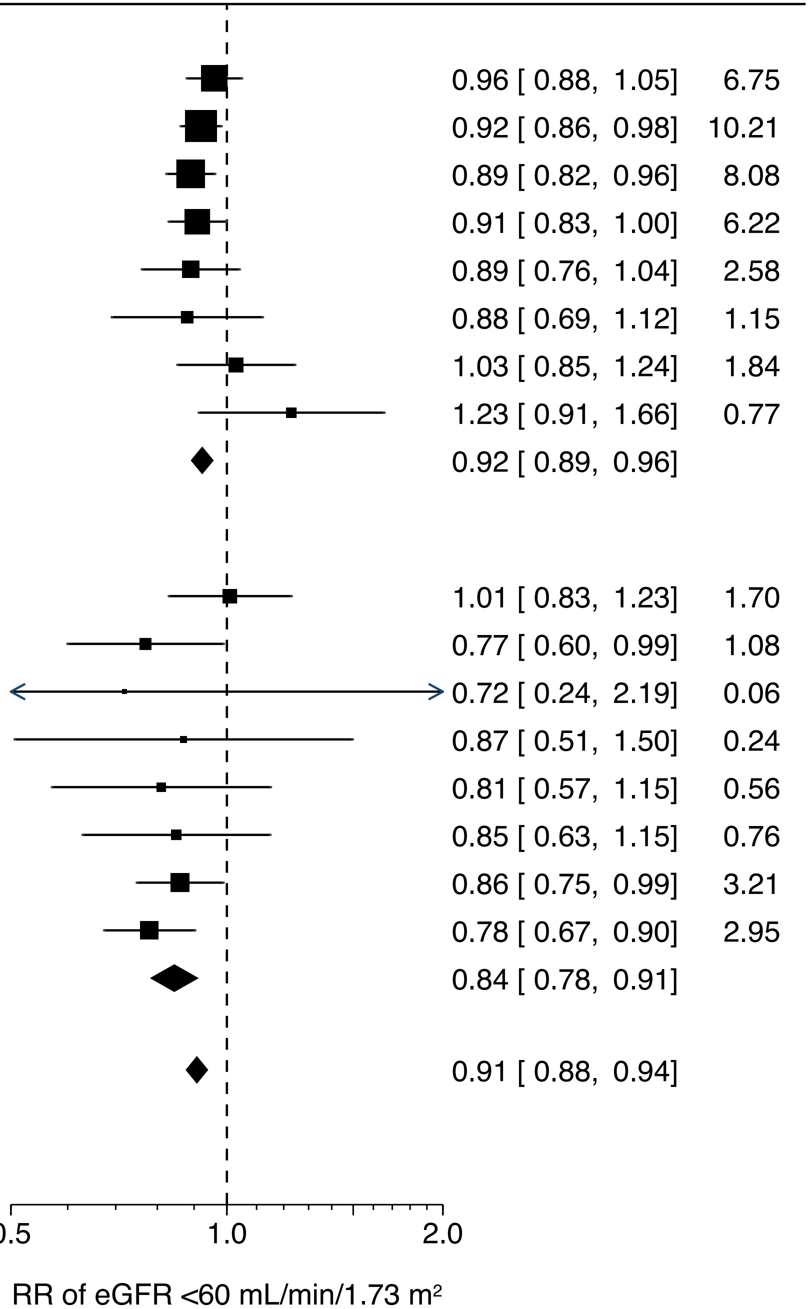

Figure S6e

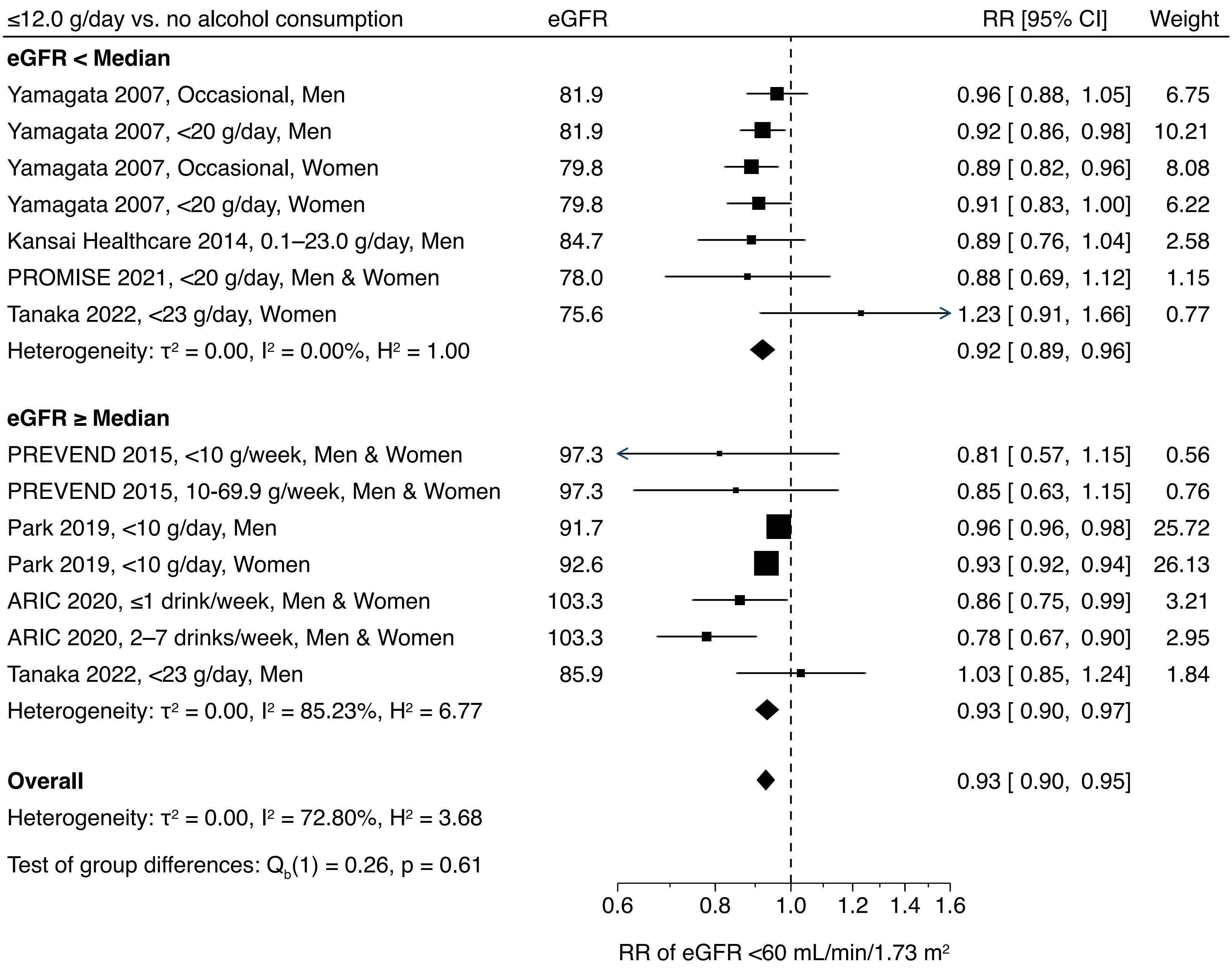
≤12.0 g/day vs. no alcohol consumption
eGFR
RR [95% CI]
Weight
eGFR < Median
Yamagata 2007, Occasional, Men 81.9 0.96 [0.88, 1.05] 6.75
Yamagata 2007, <20 g/day, Men 81.9 0.92 [0.86, 0.98] 10.21
Yamagata 2007, Occasional, Women 79.8 0.89 [0.82, 0.96] 8.08
Yamagata 2007, <20 g/day, Women 79.8 0.91 [0.83, 1.00] 6.22
Kansai Healthcare 2014, 0.1–23.0 g/day, Men 84.7 0.89 [0.76, 1.04] 2.58
PROMISE 2021, <20 g/day, Men & Women 78.0 0.88 [0.69, 1.12] 1.15
Tanaka 2022, <23 g/day, Women 75.6 1.23 [0.91, 1.66] 0.77
Heterogeneity: τ² = 0.00, I² = 0.00%, H² = 1.00 0.92 [0.89, 0.96]
eGFR ≥ Median
PREVEND 2015, <10 g/week, Men & Women 97.3 0.81 [0.57, 1.15] 0.56
PREVEND 2015, 10-69.9 g/week, Men & Women 97.3 0.85 [0.63, 1.15] 0.76
Park 2019, <10 g/day, Men 91.7 0.96 [0.96, 0.98] 25.72
Park 2019, <10 g/day, Women 92.6 0.93 [0.92, 0.94] 26.13
ARIC 2020, ≤1 drink/week, Men & Women 103.3 0.86 [0.75, 0.99] 3.21
ARIC 2020, 2–7 drinks/week, Men & Women 103.3 0.78 [0.67, 0.90] 2.95
Tanaka 2022, <23 g/day, Men 85.9 1.03 [0.85, 1.24] 1.84
Heterogeneity: τ² = 0.00, I² = 85.23%, H² = 6.77 0.93 [0.90, 0.97]
Overall 0.93 [0.90, 0.95]
Heterogeneity: τ² = 0.00, I² = 72.80%, H² = 3.68
Test of group differences: Qb(1) = 0.26, p = 0.61
0.6 0.8 1.0 1.2 1.4 1.6
RR of eGFR <60 mL/min/1.73 m²

Figure S6f

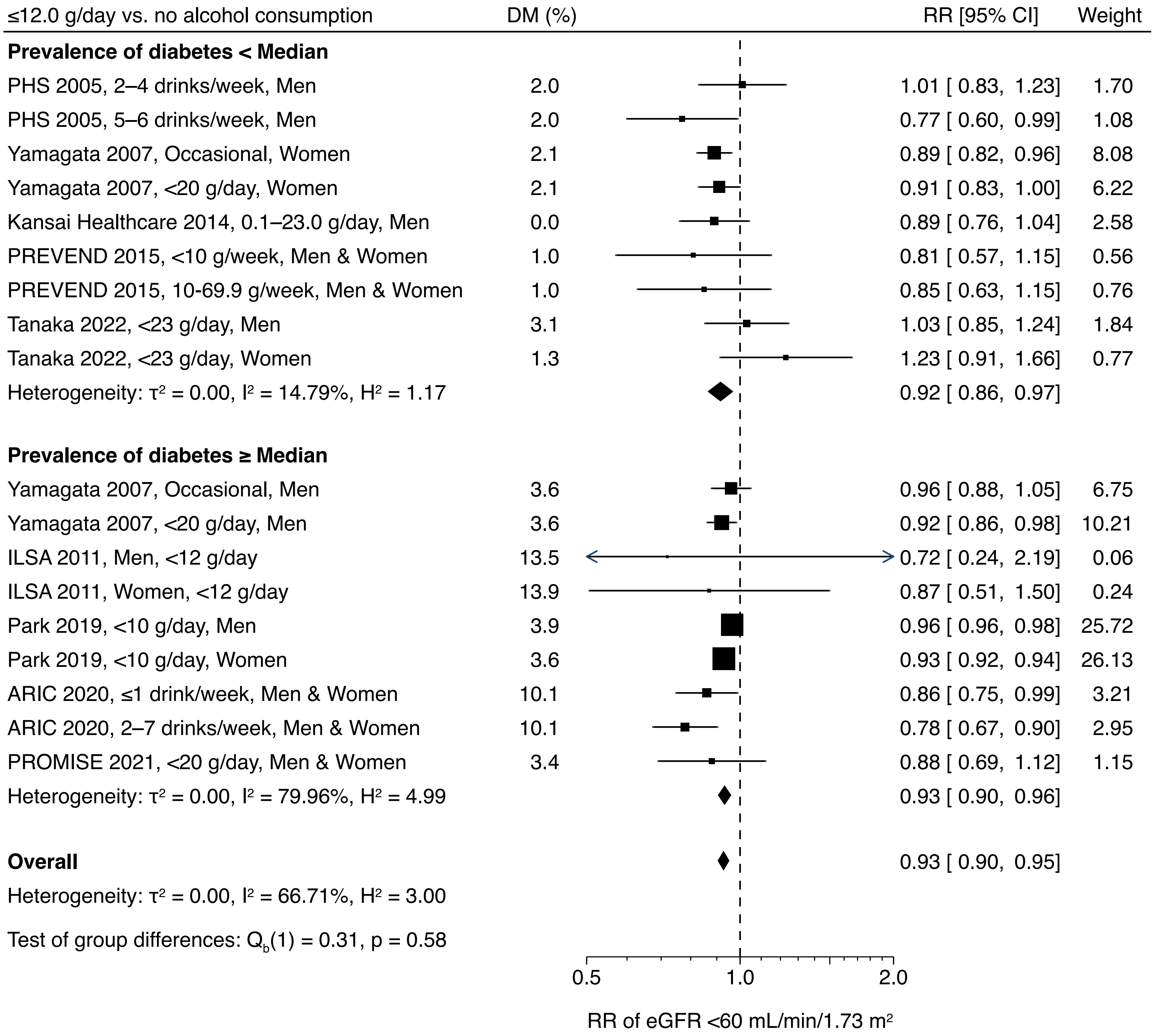

≤12.0 g/day vs. no alcohol consumption
DM (%)
RR [95% CI]
Weight
Prevalence of diabetes < Median
PHS 2005, 2–4 drinks/week, Men 2.0 1.01 [0.83, 1.23] 1.70
PHS 2005, 5–6 drinks/week, Men 2.0 0.77 [0.60, 0.99] 1.08
Yamagata 2007, Occasional, Women 2.1 0.89 [0.82, 0.96] 8.08
Yamagata 2007, <20 g/day, Women 2.1 0.91 [0.83, 1.00] 6.22
Kansai Healthcare 2014, 0.1–23.0 g/day, Men 0.0 0.89 [0.76, 1.04] 2.58
PREVEND 2015, <10 g/week, Men & Women 1.0 0.81 [0.57, 1.15] 0.56
PREVEND 2015, 10-69.9 g/week, Men & Women 1.0 0.85 [0.63, 1.15] 0.76
Tanaka 2022, <23 g/day, Men 3.1 1.03 [0.85, 1.24] 1.84
Tanaka 2022, <23 g/day, Women 1.3 1.23 [0.91, 1.66] 0.77
Heterogeneity: τ² = 0.00, I² = 14.79%, H² = 1.17 0.92 [0.86, 0.97]
Prevalence of diabetes ≥ Median
Yamagata 2007, Occasional, Men 3.6 0.96 [0.88, 1.05] 6.75
Yamagata 2007, <20 g/day, Men 3.6 0.92 [0.86, 0.98] 10.21
ILSA 2011, Men, <12 g/day 13.5 0.72 [0.24, 2.19] 0.06
ILSA 2011, Women, <12 g/day 13.9 0.87 [0.51, 1.50] 0.24
Park 2019, <10 g/day, Men 3.9 0.96 [0.96, 0.98] 25.72
Park 2019, <10 g/day, Women 3.6 0.93 [0.92, 0.94] 26.13
ARIC 2020, ≤1 drink/week, Men & Women 10.1 0.86 [0.75, 0.99] 3.21
ARIC 2020, 2–7 drinks/week, Men & Women 10.1 0.78 [0.67, 0.90] 2.95
PROMISE 2021, <20 g/day, Men & Women 3.4 0.88 [0.69, 1.12] 1.15
Heterogeneity: τ² = 0.00, I² = 79.96%, H² = 4.99 0.93 [0.90, 0.96]
Overall 0.93 [0.90, 0.95]
Heterogeneity: τ² = 0.00, I² = 66.71%, H² = 3.00
Test of group differences: Qb(1) = 0.31, p = 0.58
0.5
1.0
2.0
RR of eGFR <60 mL/min/1.73 m²

Figure S6g

≤12.0 g/day vs. no alcohol consumption	HT (%)	RR [95% CI]	Weight
**Prevalence of hypertension < Median**			
Yamagata 2007, Occasional, Women	18.9	0.89 [0.82, 0.96]	8.08
Yamagata 2007, <20 g/day, Women	18.9	0.91 [0.83, 1.00]	6.22
Kansai Healthcare 2014, 0.1–23.0 g/day, Men	0.0	0.89 [0.76, 1.04]	2.58
PREVEND 2015, <10 g/week, Men & Women	11.7	0.81 [0.57, 1.15]	0.56
PREVEND 2015, 10-69.9 g/week, Men & Women	11.7	0.85 [0.63, 1.15]	0.76
Park 2019, <10 g/day, Men	10.7	0.96 [0.96, 0.98]	25.72
Park 2019, <10 g/day, Women	13.3	0.93 [0.92, 0.94]	26.13
PROMISE 2021, <20 g/day, Men & Women	17.8	0.88 [0.69, 1.12]	1.15
Tanaka 2022, <23 g/day, Men	9.4	1.03 [0.85, 1.24]	1.84
Tanaka 2022, <23 g/day, Women	5.7	1.23 [0.91, 1.66]	0.77
Heterogeneity: $\tau^2 = 0.00$, $I^2 = 76.92\%$, $H^2 = 4.33$		0.94 [0.91, 0.97]	
**Prevalence of hypertension ≥ Median**			
PHS 2005, 2–4 drinks/week, Men	20.9	1.01 [0.83, 1.23]	1.70
PHS 2005, 5–6 drinks/week, Men	20.9	0.77 [0.60, 0.99]	1.08
Yamagata 2007, Occasional, Men	21.0	0.96 [0.88, 1.05]	6.75
Yamagata 2007, <20 g/day, Men	21.0	0.92 [0.86, 0.98]	10.21
ILSA 2011, Men, <12 g/day	64.9	0.72 [0.24, 2.19]	0.06
ILSA 2011, Women, <12 g/day	73.2	0.87 [0.51, 1.50]	0.24
ARIC 2020, ≤1 drink/week, Men & Women	25.1	0.86 [0.75, 0.99]	3.21
ARIC 2020, 2–7 drinks/week, Men & Women	25.1	0.78 [0.67, 0.90]	2.95
Heterogeneity: $\tau^2 = 0.00$, $I^2 = 25.79\%$, $H^2 = 1.35$		0.90 [0.85, 0.95]	
**Overall**		0.93 [0.90, 0.95]	
Heterogeneity: $\tau^2 = 0.00$, $I^2 = 66.71\%$, $H^2 = 3.00$			
Test of group differences: $Q_b(1) = 1.49$, $p = 0.22$			

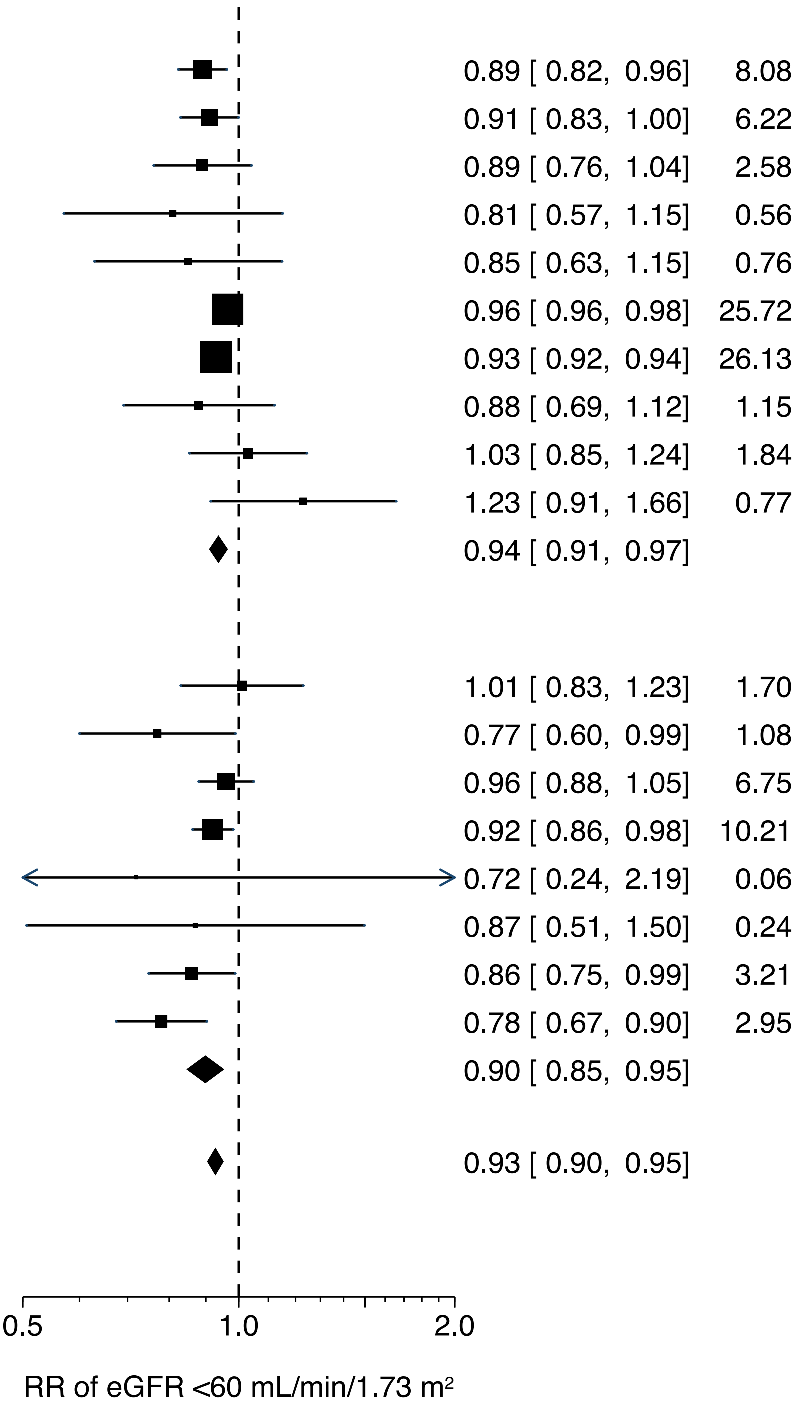

Figure S6h

≤12.0 g/day vs. no alcohol consumption	Follow-up	RR [95% CI]	Weight
**Follow-up duration < Median**			
ILSA 2011, Men, <12 g/day	3.5	0.72 [0.24, 2.19]	0.06
ILSA 2011, Women, <12 g/day	3.5	0.87 [0.51, 1.50]	0.24
Park 2019, <10 g/day, Men	6.4	0.96 [0.96, 0.98]	25.72
Park 2019, <10 g/day, Women	6.4	0.93 [0.92, 0.94]	26.13
PROMISE 2021, <20 g/day, Men & Women	5.0	0.88 [0.69, 1.12]	1.15
Tanaka 2022, <23 g/day, Men	4.0	1.03 [0.85, 1.24]	1.84
Tanaka 2022, <23 g/day, Women	4.0	1.23 [0.91, 1.66]	0.77
Heterogeneity: τ^2 = 0.00, I^2 = 82.83%, H^2 = 5.82		0.95 [0.92, 0.98]	
**Follow-up duration ≥ Median**			
PHS 2005, 2–4 drinks/week, Men	14.2	1.01 [0.83, 1.23]	1.70
PHS 2005, 5–6 drinks/week, Men	14.2	0.77 [0.60, 0.99]	1.08
Kansai Healthcare 2014, 0.1–23.0 g/day, Men	8.7	0.89 [0.76, 1.04]	2.58
PREVEND 2015, <10 g/week, Men & Women	10.2	0.81 [0.57, 1.15]	0.56
PREVEND 2015, 10-69.9 g/week, Men & Women	10.2	0.85 [0.63, 1.15]	0.76
ARIC 2020, ≤1 drink/week, Men & Women	24.0	0.86 [0.75, 0.99]	3.21
ARIC 2020, 2–7 drinks/week, Men & Women	24.0	0.78 [0.67, 0.90]	2.95
Heterogeneity: τ^2 = 0.00, I^2 = 0.00%, H^2 = 1.00		0.85 [0.80, 0.92]	
**Overall**		0.93 [0.90, 0.96]	
Heterogeneity: τ^2 = 0.00, I^2 = 72.81%, H^2 = 3.68			
Test of group differences: $Q_b(1)$ = 7.21, p = 0.01			

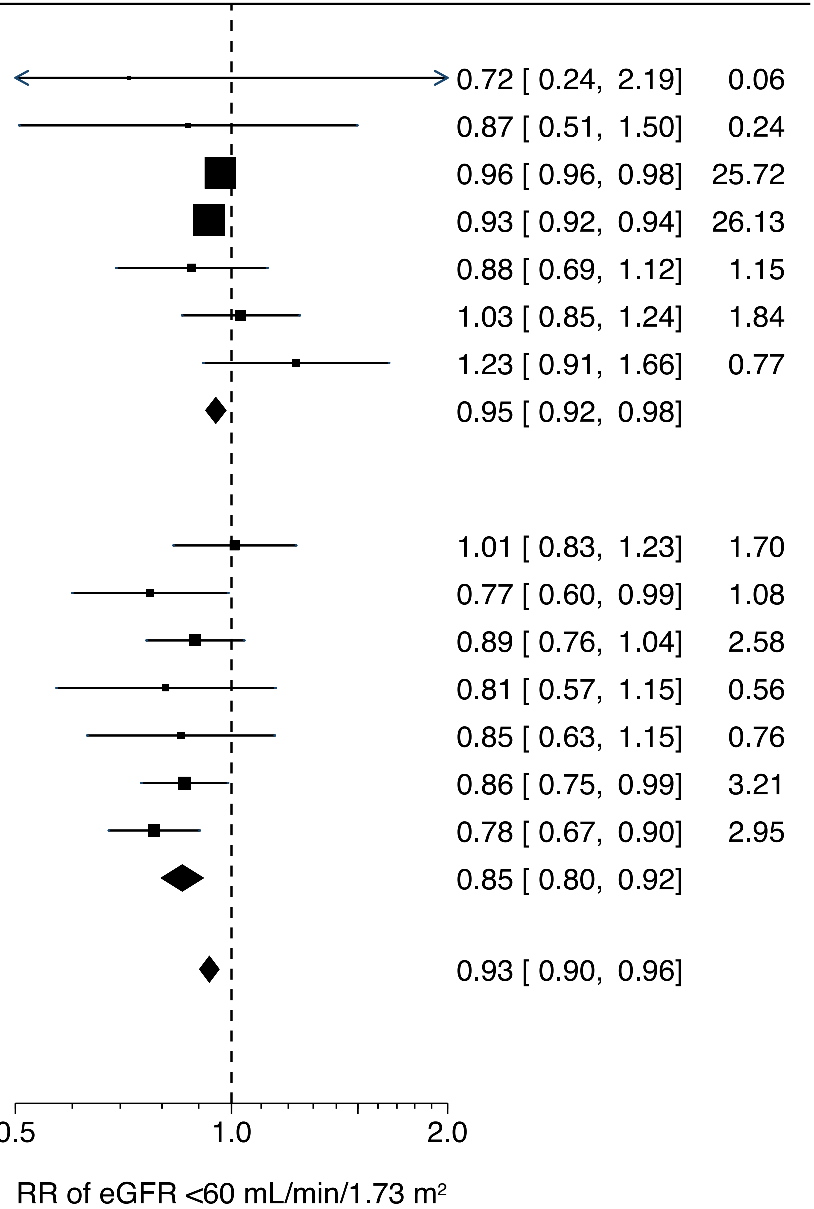

Figure S6i

≤12.0 g/day vs. no alcohol consumption	NOS	RR [95% CI]	Weight
**Newcastle-Ottawa scale (NOS) <7**			
PHS 2005, 2–4 drinks/week, Men	5	1.01 [0.83, 1.23]	1.70
PHS 2005, 5–6 drinks/week, Men	5	0.77 [0.60, 0.99]	1.08
Yamagata 2007, Occasional, Men	6	0.96 [0.88, 1.05]	6.75
Yamagata 2007, <20 g/day, Men	6	0.92 [0.86, 0.98]	10.21
Yamagata 2007, Occasional, Women	6	0.89 [0.82, 0.96]	8.08
Yamagata 2007, <20 g/day, Women	6	0.91 [0.83, 1.00]	6.22
ILSA 2011, Men, <12 g/day	6	0.72 [0.24, 2.19]	0.06
ILSA 2011, Women, <12 g/day	6	0.87 [0.51, 1.50]	0.24
Kansai Healthcare 2014, 0.1–23.0 g/day, Men	5	0.89 [0.76, 1.04]	2.58
PROMISE 2021, <20 g/day, Men & Women	6	0.88 [0.69, 1.12]	1.15
Tanaka 2022, <23 g/day, Men	5	1.03 [0.85, 1.24]	1.84
Tanaka 2022, <23 g/day, Women	5	1.23 [0.91, 1.66]	0.77
Heterogeneity: $\tau^2 = 0.00$, $I^2 = 0.00\%$, $H^2 = 1.00$		0.92 [0.89, 0.96]	
**Newcastle-Ottawa scale (NOS) ≤7**			
PREVEND 2015, <10 g/week, Men & Women	7	0.81 [0.57, 1.15]	0.56
PREVEND 2015, 10-69.9 g/week, Men & Women	7	0.85 [0.63, 1.15]	0.76
Park 2019, <10 g/day, Men	8	0.96 [0.96, 0.98]	25.72
Park 2019, <10 g/day, Women	8	0.93 [0.92, 0.94]	26.13
ARIC 2020, ≤1 drink/week, Men & Women	8	0.86 [0.75, 0.99]	3.21
ARIC 2020, 2–7 drinks/week, Men & Women	8	0.78 [0.67, 0.90]	2.95
Heterogeneity: $\tau^2 = 0.00$, $I^2 = 87.43\%$, $H^2 = 7.95$		0.93 [0.90, 0.96]	
**Overall**		0.93 [0.90, 0.95]	
Heterogeneity: $\tau^2 = 0.00$, $I^2 = 66.71\%$, $H^2 = 3.00$			
Test of group differences: $Q_b(1) = 0.07$, $p = 0.79$			

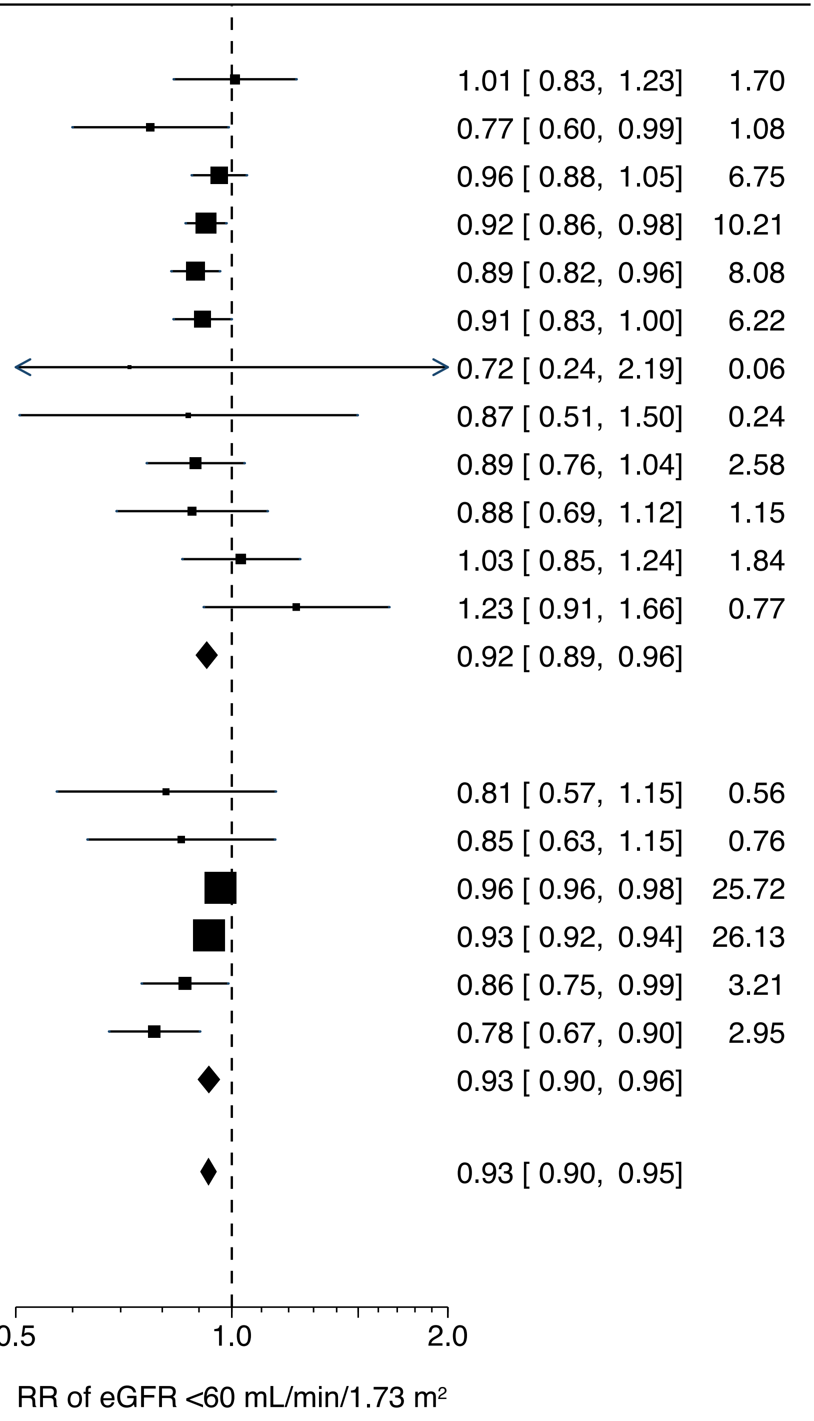

Figure S6j

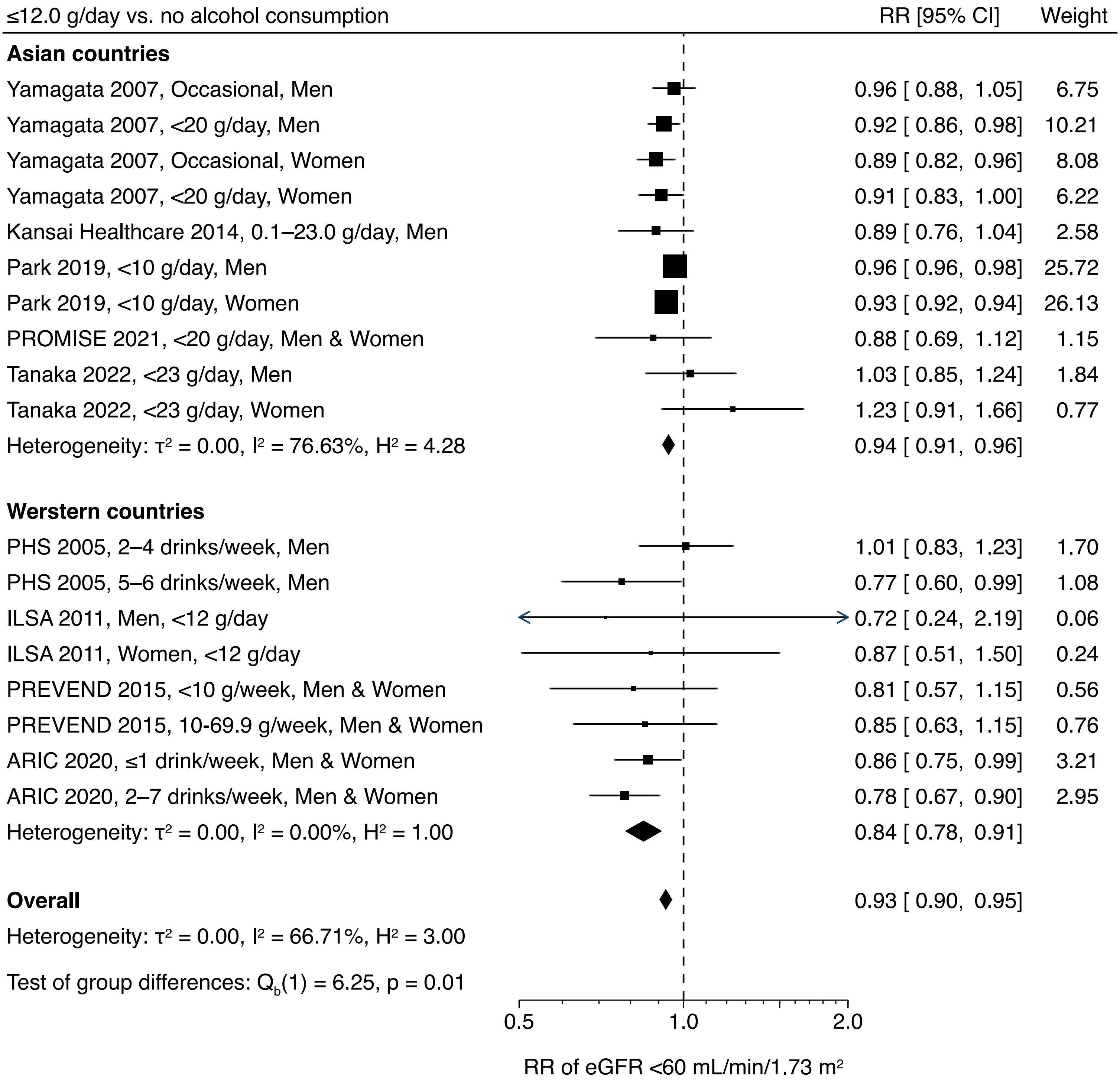
≤12.0 g/day vs. no alcohol consumption
RR [95% CI]
Weight
Asian countries
Yamagata 2007, Occasional, Men 0.96 [0.88, 1.05] 6.75
Yamagata 2007, <20 g/day, Men 0.92 [0.86, 0.98] 10.21
Yamagata 2007, Occasional, Women 0.89 [0.82, 0.96] 8.08
Yamagata 2007, <20 g/day, Women 0.91 [0.83, 1.00] 6.22
Kansai Healthcare 2014, 0.1–23.0 g/day, Men 0.89 [0.76, 1.04] 2.58
Park 2019, <10 g/day, Men 0.96 [0.96, 0.98] 25.72
Park 2019, <10 g/day, Women 0.93 [0.92, 0.94] 26.13
PROMISE 2021, <20 g/day, Men & Women 0.88 [0.69, 1.12] 1.15
Tanaka 2022, <23 g/day, Men 1.03 [0.85, 1.24] 1.84
Tanaka 2022, <23 g/day, Women 1.23 [0.91, 1.66] 0.77
Heterogeneity: $\tau^2 = 0.00$, $I^2 = 76.63\%$, $H^2 = 4.28$ 0.94 [0.91, 0.96]
Werstern countries
PHS 2005, 2–4 drinks/week, Men 1.01 [0.83, 1.23] 1.70
PHS 2005, 5–6 drinks/week, Men 0.77 [0.60, 0.99] 1.08
ILSA 2011, Men, <12 g/day 0.72 [0.24, 2.19] 0.06
ILSA 2011, Women, <12 g/day 0.87 [0.51, 1.50] 0.24
PREVEND 2015, <10 g/week, Men & Women 0.81 [0.57, 1.15] 0.56
PREVEND 2015, 10-69.9 g/week, Men & Women 0.85 [0.63, 1.15] 0.76
ARIC 2020, ≤1 drink/week, Men & Women 0.86 [0.75, 0.99] 3.21
ARIC 2020, 2–7 drinks/week, Men & Women 0.78 [0.67, 0.90] 2.95
Heterogeneity: $\tau^2 = 0.00$, $I^2 = 0.00\%$, $H^2 = 1.00$ 0.84 [0.78, 0.91]
Overall 0.93 [0.90, 0.95]
Heterogeneity: $\tau^2 = 0.00$, $I^2 = 66.71\%$, $H^2 = 3.00$
Test of group differences: $Q_b(1) = 6.25$, $p = 0.01$
0.5
1.0
2.0
RR of eGFR <60 mL/min/1.73 m^2

Figure S7a

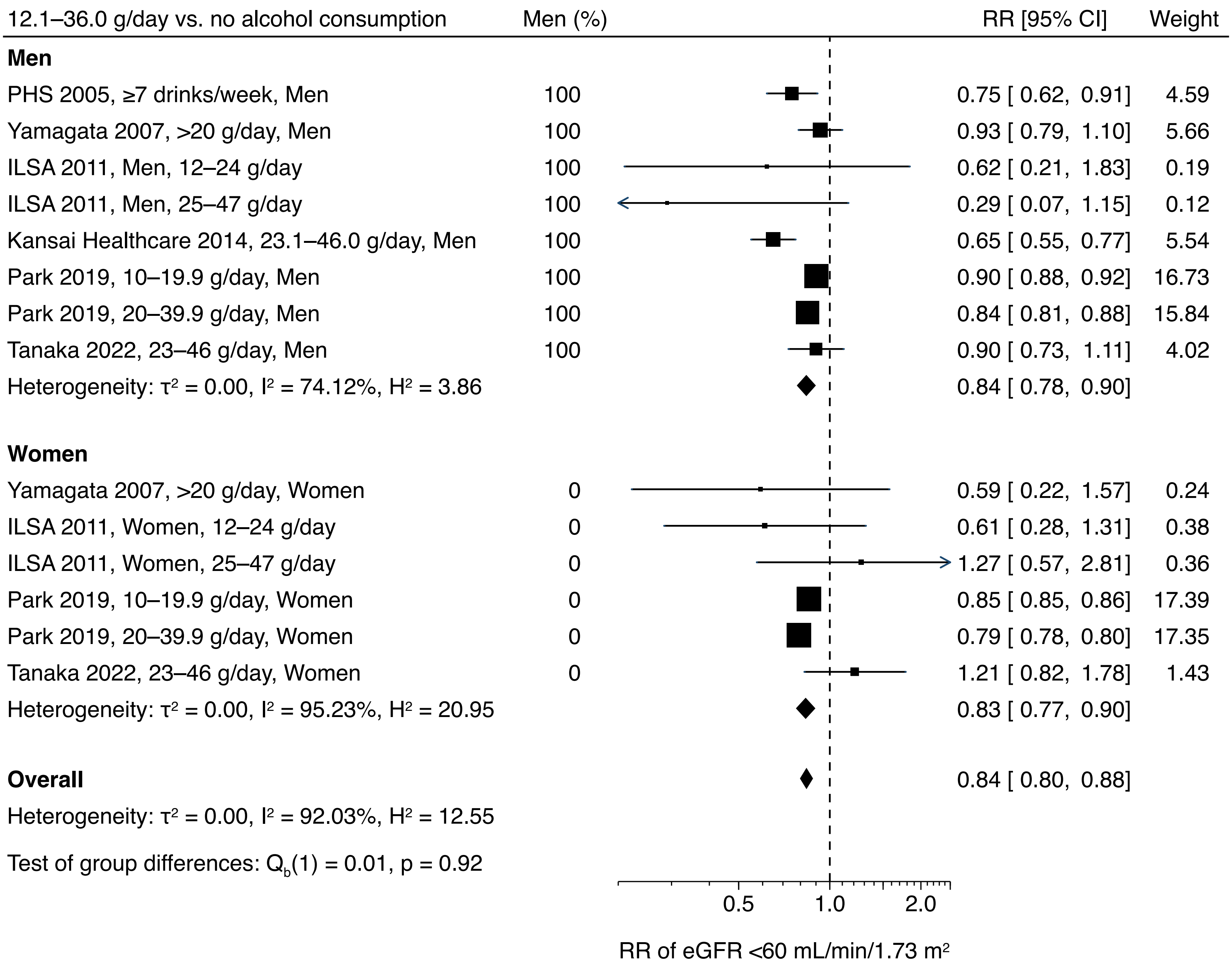
12.1–36.0 g/day vs. no alcohol consumption
Men (%)
RR [95% CI]
Weight
Men
PHS 2005, ≥7 drinks/week, Men 100 0.75 [0.62, 0.91] 4.59
Yamagata 2007, >20 g/day, Men 100 0.93 [0.79, 1.10] 5.66
ILSA 2011, Men, 12–24 g/day 100 0.62 [0.21, 1.83] 0.19
ILSA 2011, Men, 25–47 g/day 100 0.29 [0.07, 1.15] 0.12
Kansai Healthcare 2014, 23.1–46.0 g/day, Men 100 0.65 [0.55, 0.77] 5.54
Park 2019, 10–19.9 g/day, Men 100 0.90 [0.88, 0.92] 16.73
Park 2019, 20–39.9 g/day, Men 100 0.84 [0.81, 0.88] 15.84
Tanaka 2022, 23–46 g/day, Men 100 0.90 [0.73, 1.11] 4.02
Heterogeneity: τ^2 = 0.00, I^2 = 74.12%, H^2 = 3.86 0.84 [0.78, 0.90]
Women
Yamagata 2007, >20 g/day, Women 0 0.59 [0.22, 1.57] 0.24
ILSA 2011, Women, 12–24 g/day 0 0.61 [0.28, 1.31] 0.38
ILSA 2011, Women, 25–47 g/day 0 1.27 [0.57, 2.81] 0.36
Park 2019, 10–19.9 g/day, Women 0 0.85 [0.85, 0.86] 17.39
Park 2019, 20–39.9 g/day, Women 0 0.79 [0.78, 0.80] 17.35
Tanaka 2022, 23–46 g/day, Women 0 1.21 [0.82, 1.78] 1.43
Heterogeneity: τ^2 = 0.00, I^2 = 95.23%, H^2 = 20.95 0.83 [0.77, 0.90]
Overall 0.84 [0.80, 0.88]
Heterogeneity: τ^2 = 0.00, I^2 = 92.03%, H^2 = 12.55
Test of group differences: $Q_b(1)$ = 0.01, p = 0.92
0.5
1.0
2.0
RR of eGFR <60 mL/min/1.73 m^2

Figure S7b

12.1–36.0 g/day vs. no alcohol consumption	Study size	RR [95% CI]	Weight
**Study size < Median**			
PHS 2005, ≥7 drinks/week, Men	11023	0.75 [0.62, 0.91]	4.59
ILSA 2011, Men, 12–24 g/day	886	0.62 [0.21, 1.83]	0.19
ILSA 2011, Men, 25–47 g/day	886	0.29 [0.07, 1.15]	0.12
ILSA 2011, Women, 12–24 g/day	653	0.61 [0.28, 1.31]	0.38
ILSA 2011, Women, 25–47 g/day	653	1.27 [0.57, 2.81]	0.36
Kansai Healthcare 2014, 23.1–46.0 g/day, Men	9112	0.65 [0.55, 0.77]	5.54
PREVEND 2015, 70–210 g/week, Men & Women	5476	0.56 [0.38, 0.82]	1.47
PREVEND 2015, >120 g/week, Men & Women	5476	0.51 [0.25, 1.04]	0.45
Tanaka 2022, 23–46 g/day, Women	7086	1.21 [0.82, 1.78]	1.43
Heterogeneity: $\tau^2 = 0.03$, $I^2 = 46.68\%$, $H^2 = 1.88$		0.72 [0.59, 0.87]	
**Study size ≥ Median**			
Yamagata 2007, >20 g/day, Men	35491	0.93 [0.79, 1.10]	5.66
Yamagata 2007, >20 g/day, Women	71298	0.59 [0.22, 1.57]	0.24
Park 2019, 10–19.9 g/day, Men	7625277	0.90 [0.88, 0.92]	16.73
Park 2019, 20–39.9 g/day, Men	7625277	0.84 [0.81, 0.88]	15.84
Park 2019, 10–19.9 g/day, Women	6565601	0.85 [0.85, 0.86]	17.39
Park 2019, 20–39.9 g/day, Women	6565601	0.79 [0.78, 0.80]	17.35
ARIC 2020, 8–14 drinks/week, Men & Women	12692	0.61 [0.49, 0.76]	3.74
ARIC 2020, ≥15 drinks/week, Men & Women	12692	0.74 [0.58, 0.95]	3.10
PROMISE 2021, 20–39 g/day, Men & Woomen	11175	0.92 [0.63, 1.35]	1.42
Tanaka 2022, 23–46 g/day, Men	19902	0.90 [0.73, 1.11]	4.02
Heterogeneity: $\tau^2 = 0.00$, $I^2 = 94.18\%$, $H^2 = 17.18$		0.84 [0.80, 0.88]	
**Overall**		0.82 [0.78, 0.86]	
Heterogeneity: $\tau^2 = 0.00$, $I^2 = 89.89\%$, $H^2 = 9.89$			
Test of group differences: $Q_b(1) = 2.24$, $p = 0.13$			

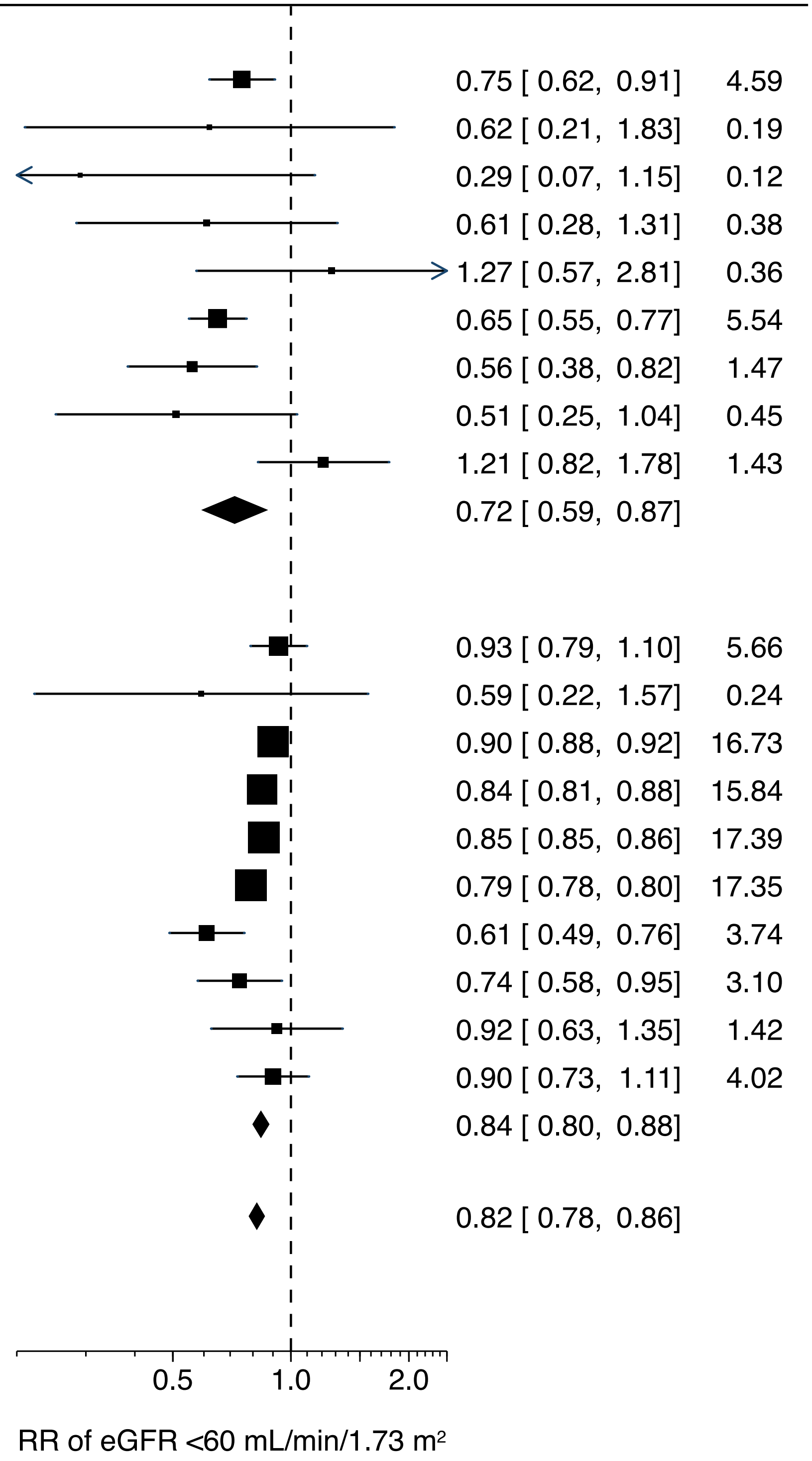

Figure S7c

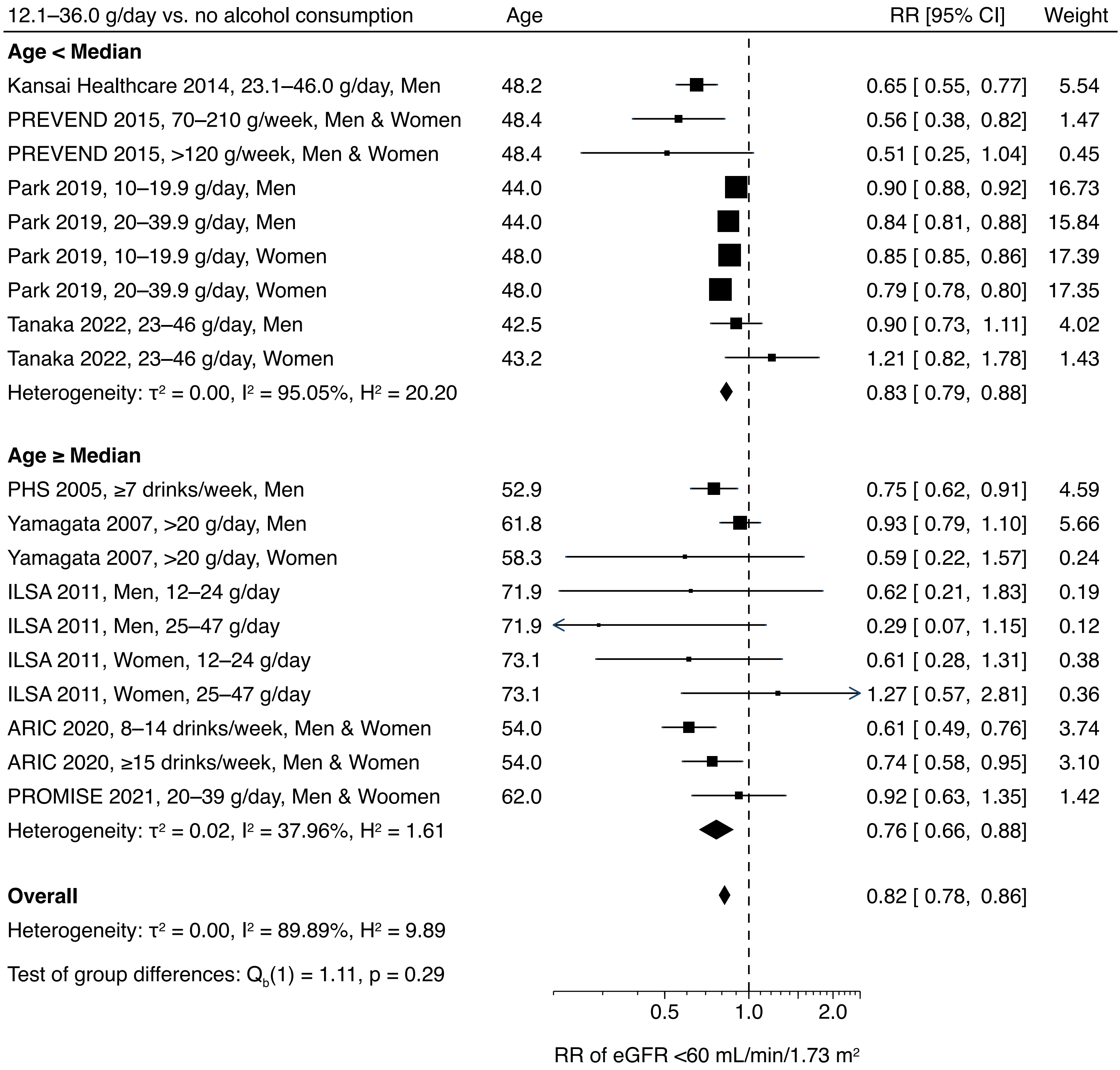
12.1–36.0 g/day vs. no alcohol consumption
Age
RR [95% CI]
Weight
Age < Median
Kansai Healthcare 2014, 23.1–46.0 g/day, Men 48.2 0.65 [0.55, 0.77] 5.54
PREVEND 2015, 70–210 g/week, Men & Women 48.4 0.56 [0.38, 0.82] 1.47
PREVEND 2015, >120 g/week, Men & Women 48.4 0.51 [0.25, 1.04] 0.45
Park 2019, 10–19.9 g/day, Men 44.0 0.90 [0.88, 0.92] 16.73
Park 2019, 20–39.9 g/day, Men 44.0 0.84 [0.81, 0.88] 15.84
Park 2019, 10–19.9 g/day, Women 48.0 0.85 [0.85, 0.86] 17.39
Park 2019, 20–39.9 g/day, Women 48.0 0.79 [0.78, 0.80] 17.35
Tanaka 2022, 23–46 g/day, Men 42.5 0.90 [0.73, 1.11] 4.02
Tanaka 2022, 23–46 g/day, Women 43.2 1.21 [0.82, 1.78] 1.43
Heterogeneity: τ² = 0.00, I² = 95.05%, H² = 20.20 0.83 [0.79, 0.88]
Age ≥ Median
PHS 2005, ≥7 drinks/week, Men 52.9 0.75 [0.62, 0.91] 4.59
Yamagata 2007, >20 g/day, Men 61.8 0.93 [0.79, 1.10] 5.66
Yamagata 2007, >20 g/day, Women 58.3 0.59 [0.22, 1.57] 0.24
ILSA 2011, Men, 12–24 g/day 71.9 0.62 [0.21, 1.83] 0.19
ILSA 2011, Men, 25–47 g/day 71.9 0.29 [0.07, 1.15] 0.12
ILSA 2011, Women, 12–24 g/day 73.1 0.61 [0.28, 1.31] 0.38
ILSA 2011, Women, 25–47 g/day 73.1 1.27 [0.57, 2.81] 0.36
ARIC 2020, 8–14 drinks/week, Men & Women 54.0 0.61 [0.49, 0.76] 3.74
ARIC 2020, ≥15 drinks/week, Men & Women 54.0 0.74 [0.58, 0.95] 3.10
PROMISE 2021, 20–39 g/day, Men & Woomen 62.0 0.92 [0.63, 1.35] 1.42
Heterogeneity: τ² = 0.02, I² = 37.96%, H² = 1.61 0.76 [0.66, 0.88]
Overall 0.82 [0.78, 0.86]
Heterogeneity: τ² = 0.00, I² = 89.89%, H² = 9.89
Test of group differences: Qb(1) = 1.11, p = 0.29
0.5
1.0
2.0
RR of eGFR <60 mL/min/1.73 m²

Figure S7d

12.1–36.0 g/day vs. no alcohol consumption	BMI	RR [95% CI]	Weight
**Body mass index (BMI) < Median**			
PHS 2005, ≥7 drinks/week, Men	24.9	0.75 [0.62, 0.91]	4.59
Yamagata 2007, >20 g/day, Men	23.2	0.93 [0.79, 1.10]	5.66
Yamagata 2007, >20 g/day, Women	23.5	0.59 [0.22, 1.57]	0.24
Kansai Healthcare 2014, 23.1–46.0 g/day, Men	23.2	0.65 [0.55, 0.77]	5.54
PROMISE 2021, 20–39 g/day, Men & Woomen	22.3	0.92 [0.63, 1.35]	1.42
Tanaka 2022, 23–46 g/day, Men	23.4	0.90 [0.73, 1.11]	4.02
Tanaka 2022, 23–46 g/day, Women	21.4	1.21 [0.82, 1.78]	1.43
Heterogeneity: $\tau^2 = 0.02$, $I^2 = 62.10\%$, $H^2 = 2.64$		0.84 [0.72, 0.98]	
**Body mass index (BMI) ≥ Median**			
ILSA 2011, Men, 12–24 g/day	26.5	0.62 [0.21, 1.83]	0.19
ILSA 2011, Men, 25–47 g/day	26.5	0.29 [0.07, 1.15]	0.12
ILSA 2011, Women, 12–24 g/day	27.6	0.61 [0.28, 1.31]	0.38
ILSA 2011, Women, 25–47 g/day	27.6	1.27 [0.57, 2.81]	0.36
PREVEND 2015, 70–210 g/week, Men & Women	25.7	0.56 [0.38, 0.82]	1.47
PREVEND 2015, >120 g/week, Men & Women	25.7	0.51 [0.25, 1.04]	0.45
ARIC 2020, 8–14 drinks/week, Men & Women	27.4	0.61 [0.49, 0.76]	3.74
ARIC 2020, ≥15 drinks/week, Men & Women	27.4	0.74 [0.58, 0.95]	3.10
Heterogeneity: $\tau^2 = 0.00$, $I^2 = 0.00\%$, $H^2 = 1.00$		0.65 [0.56, 0.74]	
**Overall**		0.76 [0.67, 0.86]	
Heterogeneity: $\tau^2 = 0.03$, $I^2 = 53.18\%$, $H^2 = 2.14$			
Test of group differences: $Q_b(1) = 5.85$, $p = 0.02$			

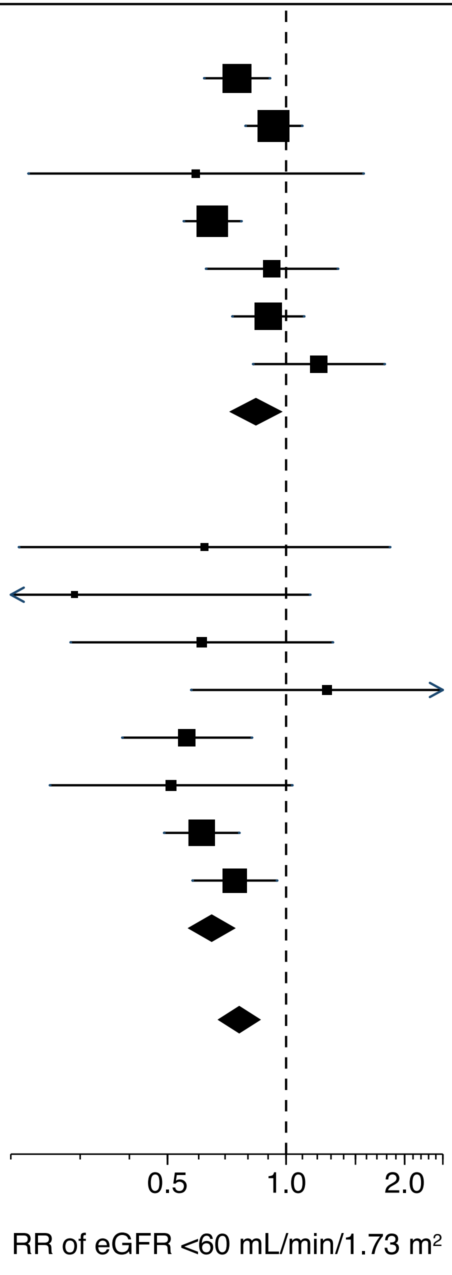

Figure S7e

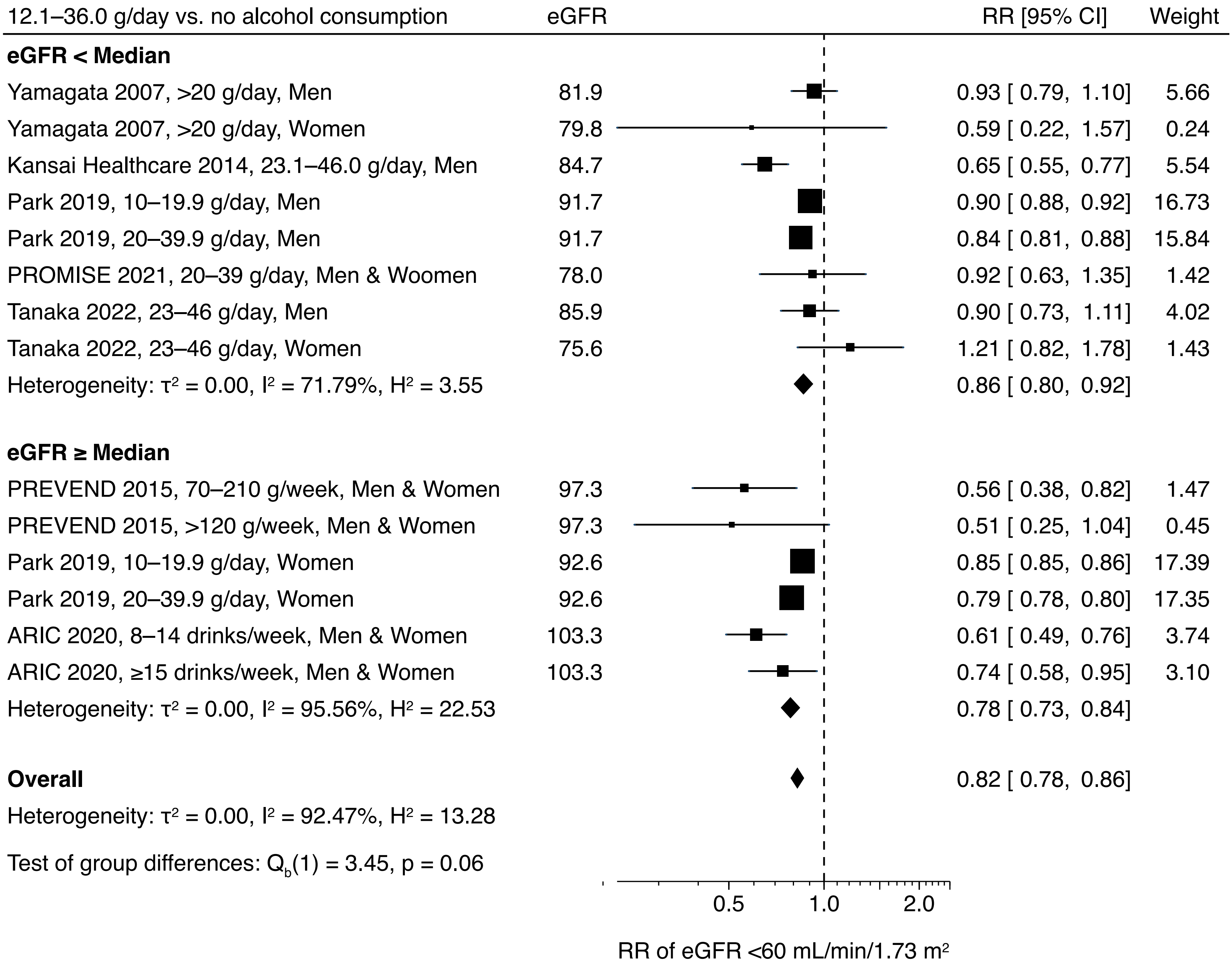
12.1–36.0 g/day vs. no alcohol consumption
eGFR
RR [95% CI]
Weight
eGFR < Median
Yamagata 2007, >20 g/day, Men 81.9 0.93 [0.79, 1.10] 5.66
Yamagata 2007, >20 g/day, Women 79.8 0.59 [0.22, 1.57] 0.24
Kansai Healthcare 2014, 23.1–46.0 g/day, Men 84.7 0.65 [0.55, 0.77] 5.54
Park 2019, 10–19.9 g/day, Men 91.7 0.90 [0.88, 0.92] 16.73
Park 2019, 20–39.9 g/day, Men 91.7 0.84 [0.81, 0.88] 15.84
PROMISE 2021, 20–39 g/day, Men & Woomen 78.0 0.92 [0.63, 1.35] 1.42
Tanaka 2022, 23–46 g/day, Men 85.9 0.90 [0.73, 1.11] 4.02
Tanaka 2022, 23–46 g/day, Women 75.6 1.21 [0.82, 1.78] 1.43
Heterogeneity: τ² = 0.00, I² = 71.79%, H² = 3.55 0.86 [0.80, 0.92]
eGFR ≥ Median
PREVEND 2015, 70–210 g/week, Men & Women 97.3 0.56 [0.38, 0.82] 1.47
PREVEND 2015, >120 g/week, Men & Women 97.3 0.51 [0.25, 1.04] 0.45
Park 2019, 10–19.9 g/day, Women 92.6 0.85 [0.85, 0.86] 17.39
Park 2019, 20–39.9 g/day, Women 92.6 0.79 [0.78, 0.80] 17.35
ARIC 2020, 8–14 drinks/week, Men & Women 103.3 0.61 [0.49, 0.76] 3.74
ARIC 2020, ≥15 drinks/week, Men & Women 103.3 0.74 [0.58, 0.95] 3.10
Heterogeneity: τ² = 0.00, I² = 95.56%, H² = 22.53 0.78 [0.73, 0.84]
Overall 0.82 [0.78, 0.86]
Heterogeneity: τ² = 0.00, I² = 92.47%, H² = 13.28
Test of group differences: Qb(1) = 3.45, p = 0.06
0.5
1.0
2.0
RR of eGFR <60 mL/min/1.73 m²

Figure S7f

12.1–36.0 g/day vs. no alcohol consumption	DM (%)	RR [95% CI]	Weight
**Prevalence of diabetes < Median**			
PHS 2005, ≥7 drinks/week, Men	2.0	0.75 [0.62, 0.91]	4.59
Yamagata 2007, >20 g/day, Men	3.6	0.93 [0.79, 1.10]	5.66
Yamagata 2007, >20 g/day, Women	2.1	0.59 [0.22, 1.57]	0.24
Kansai Healthcare 2014, 23.1–46.0 g/day, Men	0.0	0.65 [0.55, 0.77]	5.54
PREVEND 2015, 70–210 g/week, Men & Women	1.0	0.56 [0.38, 0.82]	1.47
PREVEND 2015, >120 g/week, Men & Women	1.0	0.51 [0.25, 1.04]	0.45
Park 2019, 10–19.9 g/day, Women	3.6	0.85 [0.85, 0.86]	17.39
Park 2019, 20–39.9 g/day, Women	3.6	0.79 [0.78, 0.80]	17.35
PROMISE 2021, 20–39 g/day, Men & Woomen	3.4	0.92 [0.63, 1.35]	1.42
Tanaka 2022, 23–46 g/day, Men	3.1	0.90 [0.73, 1.11]	4.02
Tanaka 2022, 23–46 g/day, Women	1.3	1.21 [0.82, 1.78]	1.43
Heterogeneity: $\tau^2 = 0.00$, $I^2 = 91.70\%$, $H^2 = 12.05$		0.81 [0.76, 0.86]	
**Prevalence of diabetes ≥ Median**			
ILSA 2011, Men, 12–24 g/day	13.5	0.62 [0.21, 1.83]	0.19
ILSA 2011, Men, 25–47 g/day	13.5	0.29 [0.07, 1.15]	0.12
ILSA 2011, Women, 12–24 g/day	13.9	0.61 [0.28, 1.31]	0.38
ILSA 2011, Women, 25–47 g/day	13.9	1.27 [0.57, 2.81]	0.36
Park 2019, 10–19.9 g/day, Men	3.9	0.90 [0.88, 0.92]	16.73
Park 2019, 20–39.9 g/day, Men	3.9	0.84 [0.81, 0.88]	15.84
ARIC 2020, 8–14 drinks/week, Men & Women	10.1	0.61 [0.49, 0.76]	3.74
ARIC 2020, ≥15 drinks/week, Men & Women	10.1	0.74 [0.58, 0.95]	3.10
Heterogeneity: $\tau^2 = 0.00$, $I^2 = 72.84\%$, $H^2 = 3.68$		0.82 [0.76, 0.89]	
**Overall**		0.82 [0.78, 0.86]	
Heterogeneity: $\tau^2 = 0.00$, $I^2 = 89.89\%$, $H^2 = 9.89$			
Test of group differences: $Q_b(1) = 0.06$, $p = 0.81$			

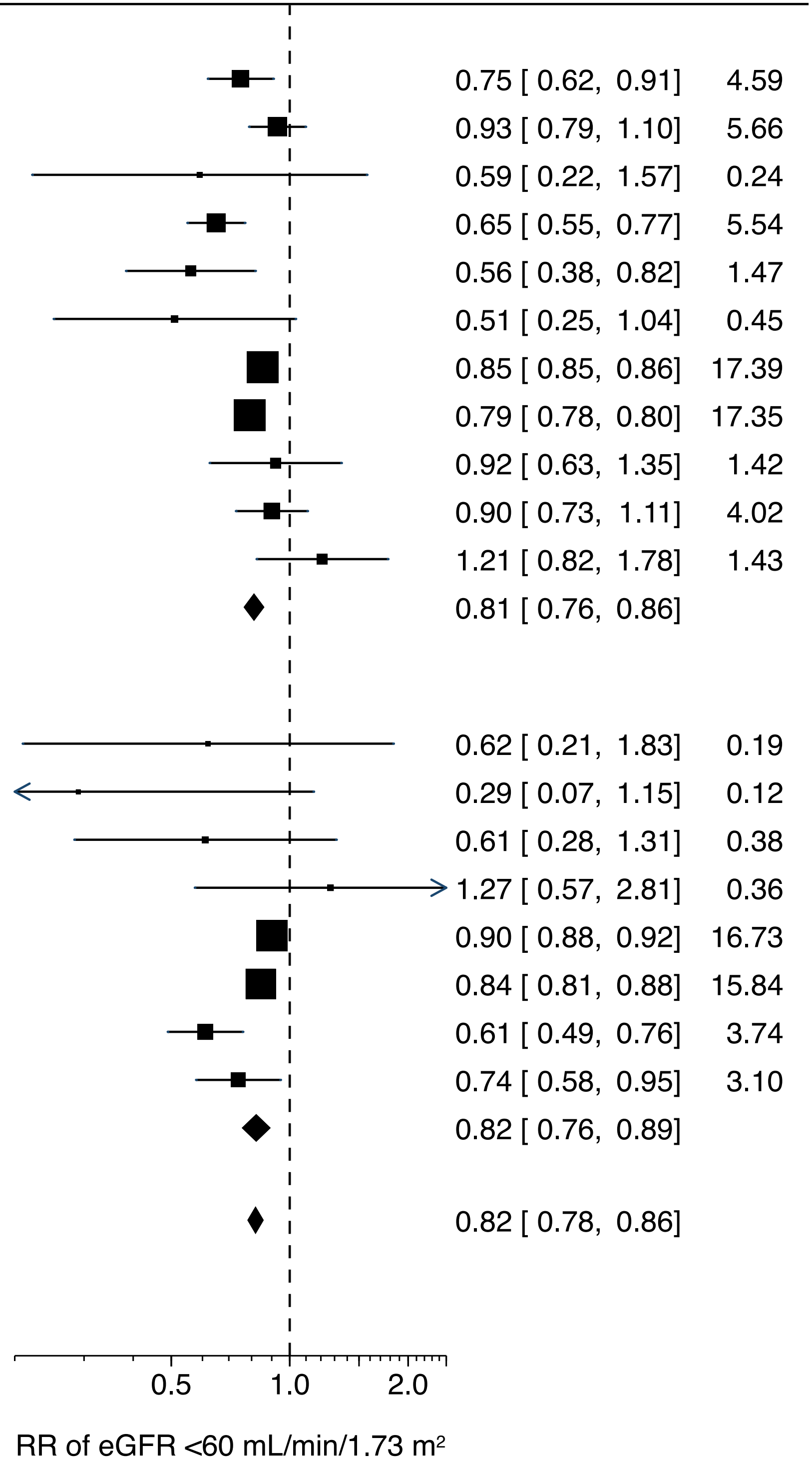

Figure S7g

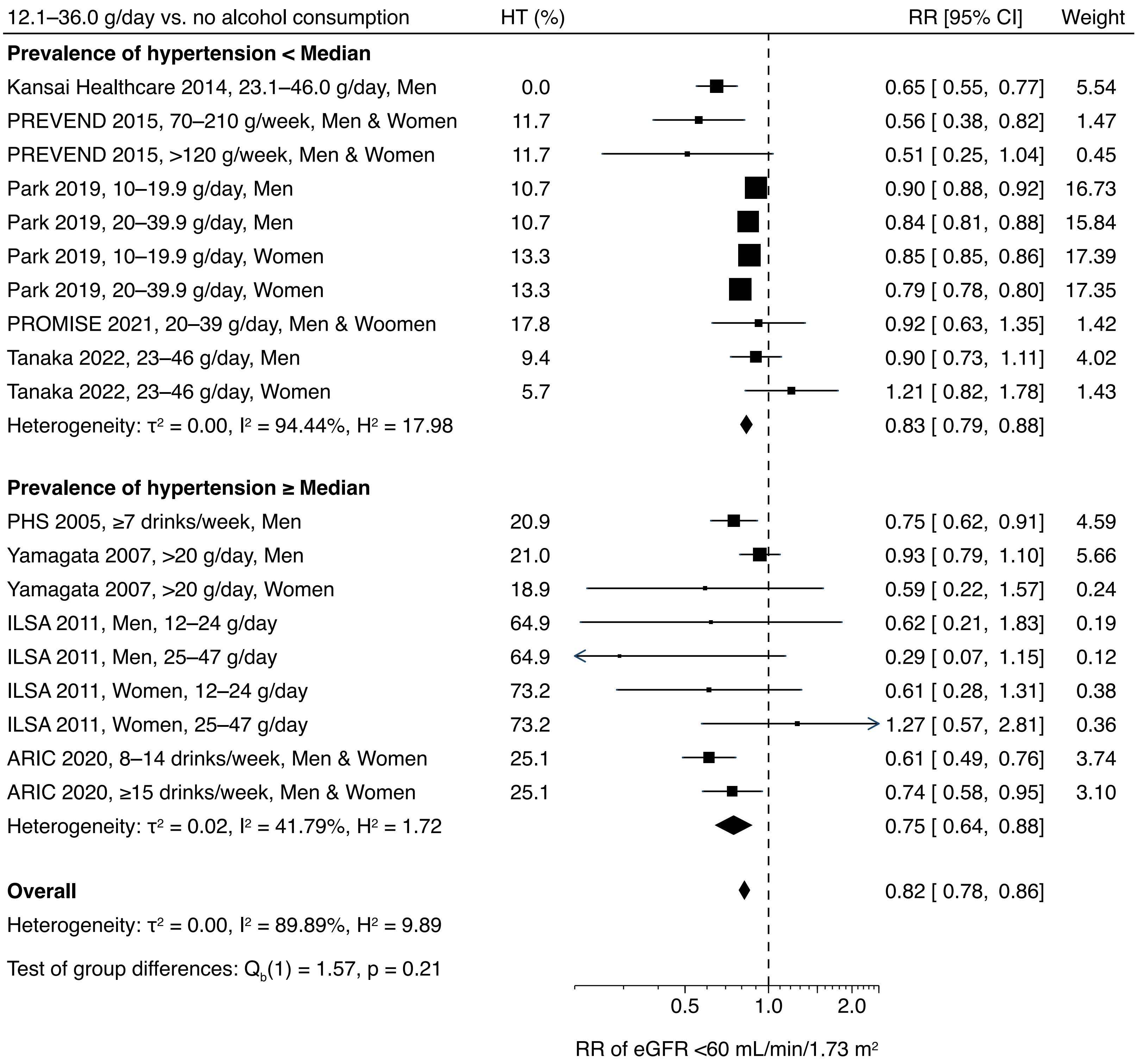
12.1–36.0 g/day vs. no alcohol consumption
HT (%)
RR [95% CI]
Weight
Prevalence of hypertension < Median
Kansai Healthcare 2014, 23.1–46.0 g/day, Men 0.0 0.65 [0.55, 0.77] 5.54
PREVEND 2015, 70–210 g/week, Men & Women 11.7 0.56 [0.38, 0.82] 1.47
PREVEND 2015, >120 g/week, Men & Women 11.7 0.51 [0.25, 1.04] 0.45
Park 2019, 10–19.9 g/day, Men 10.7 0.90 [0.88, 0.92] 16.73
Park 2019, 20–39.9 g/day, Men 10.7 0.84 [0.81, 0.88] 15.84
Park 2019, 10–19.9 g/day, Women 13.3 0.85 [0.85, 0.86] 17.39
Park 2019, 20–39.9 g/day, Women 13.3 0.79 [0.78, 0.80] 17.35
PROMISE 2021, 20–39 g/day, Men & Woomen 17.8 0.92 [0.63, 1.35] 1.42
Tanaka 2022, 23–46 g/day, Men 9.4 0.90 [0.73, 1.11] 4.02
Tanaka 2022, 23–46 g/day, Women 5.7 1.21 [0.82, 1.78] 1.43
Heterogeneity: τ² = 0.00, I² = 94.44%, H² = 17.98 0.83 [0.79, 0.88]
Prevalence of hypertension ≥ Median
PHS 2005, ≥7 drinks/week, Men 20.9 0.75 [0.62, 0.91] 4.59
Yamagata 2007, >20 g/day, Men 21.0 0.93 [0.79, 1.10] 5.66
Yamagata 2007, >20 g/day, Women 18.9 0.59 [0.22, 1.57] 0.24
ILSA 2011, Men, 12–24 g/day 64.9 0.62 [0.21, 1.83] 0.19
ILSA 2011, Men, 25–47 g/day 64.9 0.29 [0.07, 1.15] 0.12
ILSA 2011, Women, 12–24 g/day 73.2 0.61 [0.28, 1.31] 0.38
ILSA 2011, Women, 25–47 g/day 73.2 1.27 [0.57, 2.81] 0.36
ARIC 2020, 8–14 drinks/week, Men & Women 25.1 0.61 [0.49, 0.76] 3.74
ARIC 2020, ≥15 drinks/week, Men & Women 25.1 0.74 [0.58, 0.95] 3.10
Heterogeneity: τ² = 0.02, I² = 41.79%, H² = 1.72 0.75 [0.64, 0.88]
Overall 0.82 [0.78, 0.86]
Heterogeneity: τ² = 0.00, I² = 89.89%, H² = 9.89
Test of group differences: Q_b(1) = 1.57, p = 0.21
0.5
1.0
2.0
RR of eGFR <60 mL/min/1.73 m²

Figure S7h

12.1–36.0 g/day vs. no alcohol consumption	Follow-up	RR [95% CI]	Weight
**Follow-up duration < Median**			
ILSA 2011, Men, 12–24 g/day	3.5	0.62 [0.21, 1.83]	0.19
ILSA 2011, Men, 25–47 g/day	3.5	0.29 [0.07, 1.15]	0.12
ILSA 2011, Women, 12–24 g/day	3.5	0.61 [0.28, 1.31]	0.38
ILSA 2011, Women, 25–47 g/day	3.5	1.27 [0.57, 2.81]	0.36
PROMISE 2021, 20–39 g/day, Men & Woomen	5.0	0.92 [0.63, 1.35]	1.42
Tanaka 2022, 23–46 g/day, Men	4.0	0.90 [0.73, 1.11]	4.02
Tanaka 2022, 23–46 g/day, Women	4.0	1.21 [0.82, 1.78]	1.43
Heterogeneity: $\tau^2 = 0.01$, $I^2 = 13.49\%$, $H^2 = 1.16$		0.92 [0.76, 1.12]	
**Follow-up duration ≥ Median**			
PHS 2005, ≥7 drinks/week, Men	14.2	0.75 [0.62, 0.91]	4.59
Kansai Healthcare 2014, 23.1–46.0 g/day, Men	8.7	0.65 [0.55, 0.77]	5.54
PREVEND 2015, 70–210 g/week, Men & Women	10.2	0.56 [0.38, 0.82]	1.47
PREVEND 2015, >120 g/week, Men & Women	10.2	0.51 [0.25, 1.04]	0.45
Park 2019, 10–19.9 g/day, Men	6.4	0.90 [0.88, 0.92]	16.73
Park 2019, 20–39.9 g/day, Men	6.4	0.84 [0.81, 0.88]	15.84
Park 2019, 10–19.9 g/day, Women	6.4	0.85 [0.85, 0.86]	17.39
Park 2019, 20–39.9 g/day, Women	6.4	0.79 [0.78, 0.80]	17.35
ARIC 2020, 8–14 drinks/week, Men & Women	24.0	0.61 [0.49, 0.76]	3.74
ARIC 2020, ≥15 drinks/week, Men & Women	24.0	0.74 [0.58, 0.95]	3.10
Heterogeneity: $\tau^2 = 0.00$, $I^2 = 94.61\%$, $H^2 = 18.56$		0.80 [0.76, 0.85]	
**Overall**		0.81 [0.77, 0.85]	

Heterogeneity: $\tau^2 = 0.00$, $I^2 = 90.90\%$, $H^2 = 10.99$

Test of group differences: $Q_b(1) = 1.94$, $p = 0.16$

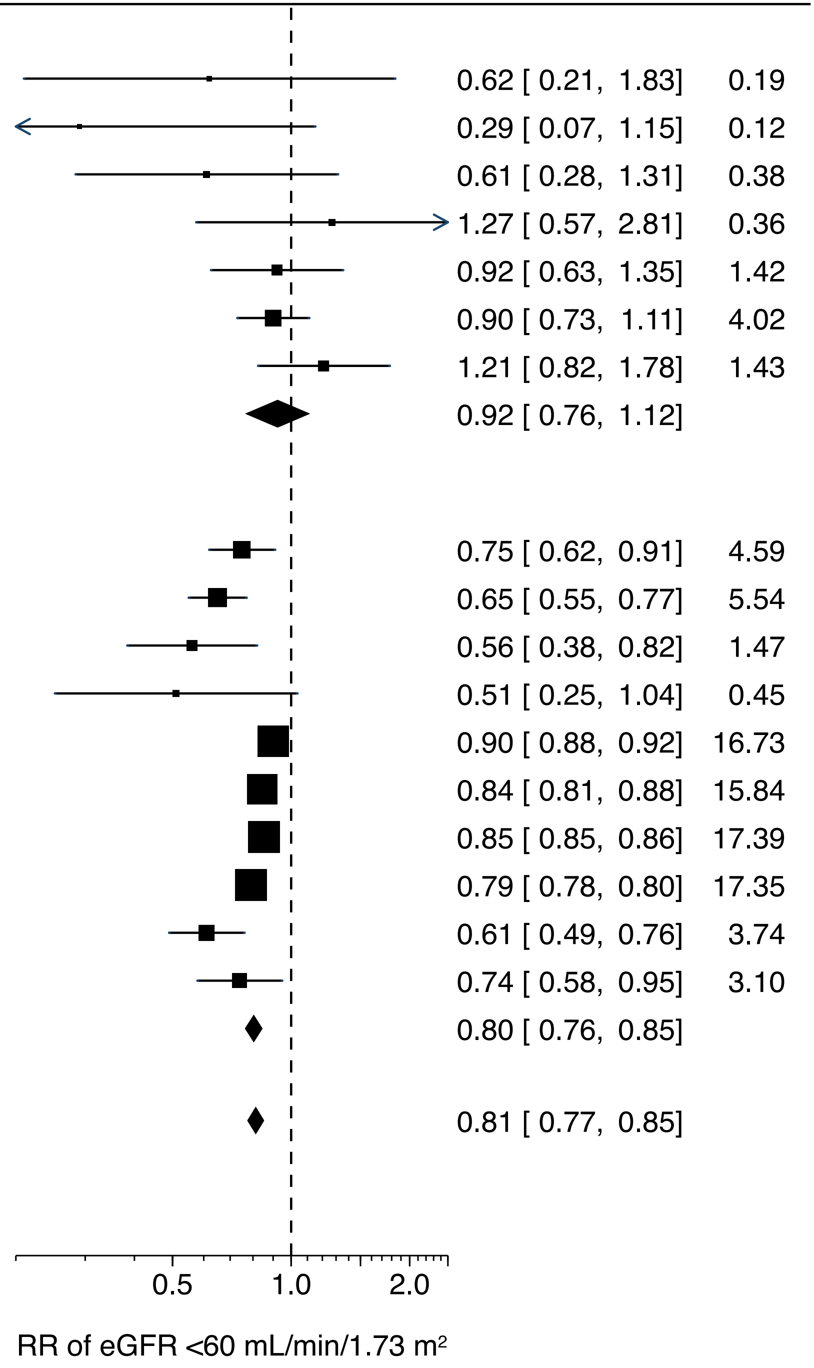

Figure S7i

12.1–36.0 g/day vs. no alcohol consumption	NOS	RR [95% CI]	Weight
**Newcastle-Ottawa scale (NOS) <7**			
PHS 2005, ≥7 drinks/week, Men	5	0.75 [0.62, 0.91]	4.59
Yamagata 2007, >20 g/day, Men	6	0.93 [0.79, 1.10]	5.66
Yamagata 2007, >20 g/day, Women	6	0.59 [0.22, 1.57]	0.24
ILSA 2011, Men, 12–24 g/day	6	0.62 [0.21, 1.83]	0.19
ILSA 2011, Men, 25–47 g/day	6	0.29 [0.07, 1.15]	0.12
ILSA 2011, Women, 12–24 g/day	6	0.61 [0.28, 1.31]	0.38
ILSA 2011, Women, 25–47 g/day	6	1.27 [0.57, 2.81]	0.36
Kansai Healthcare 2014, 23.1–46.0 g/day, Men	5	0.65 [0.55, 0.77]	5.54
PROMISE 2021, 20–39 g/day, Men & Woomen	6	0.92 [0.63, 1.35]	1.42
Tanaka 2022, 23–46 g/day, Men	5	0.90 [0.73, 1.11]	4.02
Tanaka 2022, 23–46 g/day, Women	5	1.21 [0.82, 1.78]	1.43
Heterogeneity: $\tau^2 = 0.02$, $I^2 = 49.94\%$, $H^2 = 2.00$		0.83 [0.71, 0.96]	
**Newcastle-Ottawa scale (NOS) ≥7**			
PREVEND 2015, 70–210 g/week, Men & Women	7	0.56 [0.38, 0.82]	1.47
PREVEND 2015, >120 g/week, Men & Women	7	0.51 [0.25, 1.04]	0.45
Park 2019, 10–19.9 g/day, Men	8	0.90 [0.88, 0.92]	16.73
Park 2019, 20–39.9 g/day, Men	8	0.84 [0.81, 0.88]	15.84
Park 2019, 10–19.9 g/day, Women	8	0.85 [0.85, 0.86]	17.39
Park 2019, 20–39.9 g/day, Women	8	0.79 [0.78, 0.80]	17.35
ARIC 2020, 8–14 drinks/week, Men & Women	8	0.61 [0.49, 0.76]	3.74
ARIC 2020, ≥15 drinks/week, Men & Women	8	0.74 [0.58, 0.95]	3.10
Heterogeneity: $\tau^2 = 0.00$, $I^2 = 95.56\%$, $H^2 = 22.53$		0.82 [0.78, 0.87]	
**Overall**		0.82 [0.78, 0.86]	
Heterogeneity: $\tau^2 = 0.00$, $I^2 = 89.89\%$, $H^2 = 9.89$			
Test of group differences: $Q_b(1) = 0.01$, $p = 0.93$			

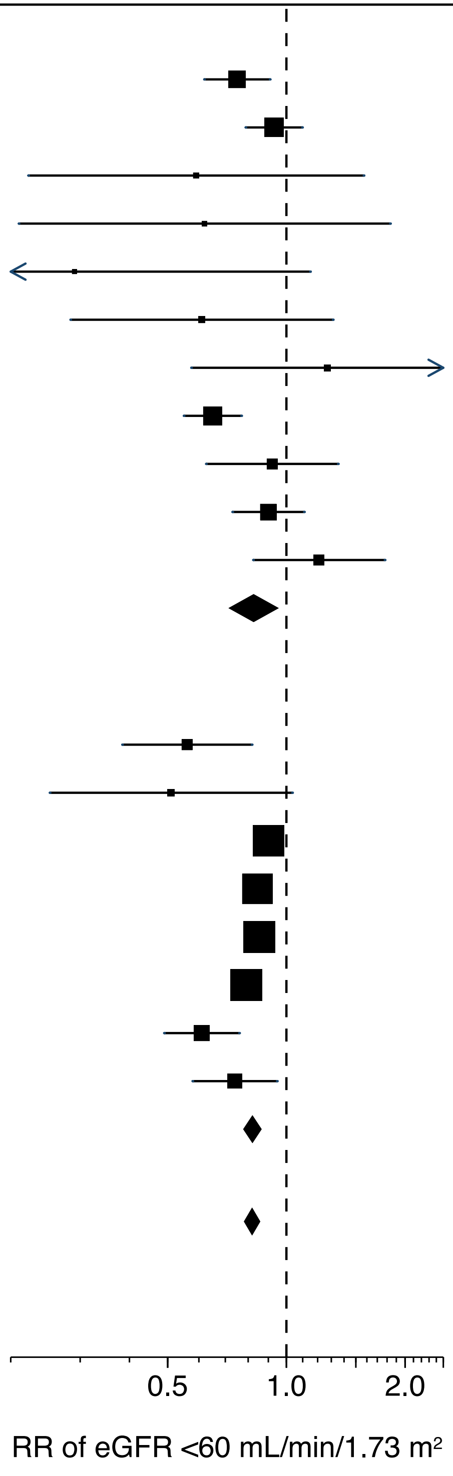

Figure S7j

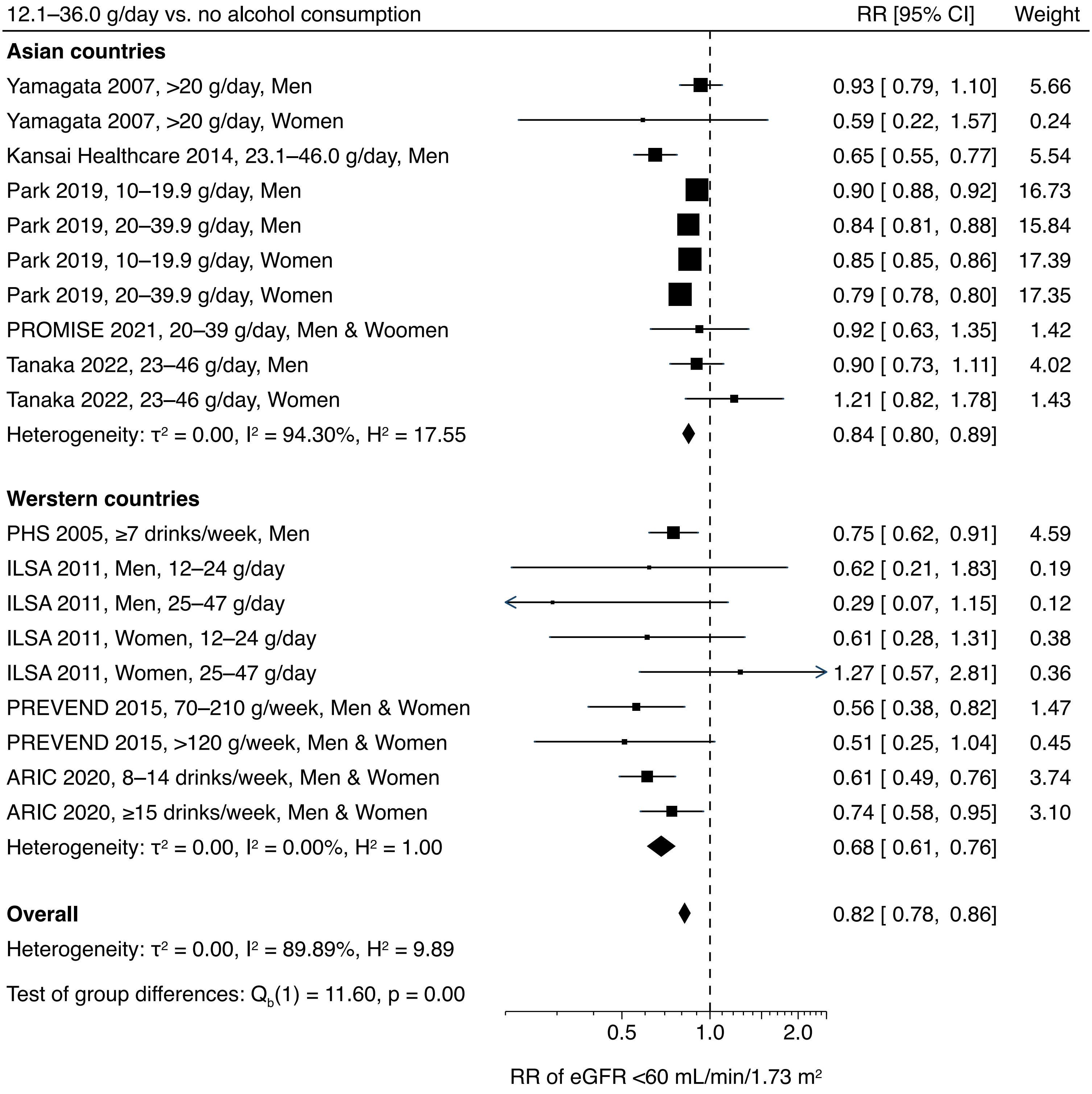
12.1–36.0 g/day vs. no alcohol consumption
RR [95% CI]
Weight
Asian countries
Yamagata 2007, >20 g/day, Men 0.93 [0.79, 1.10] 5.66
Yamagata 2007, >20 g/day, Women 0.59 [0.22, 1.57] 0.24
Kansai Healthcare 2014, 23.1–46.0 g/day, Men 0.65 [0.55, 0.77] 5.54
Park 2019, 10–19.9 g/day, Men 0.90 [0.88, 0.92] 16.73
Park 2019, 20–39.9 g/day, Men 0.84 [0.81, 0.88] 15.84
Park 2019, 10–19.9 g/day, Women 0.85 [0.85, 0.86] 17.39
Park 2019, 20–39.9 g/day, Women 0.79 [0.78, 0.80] 17.35
PROMISE 2021, 20–39 g/day, Men & Woomen 0.92 [0.63, 1.35] 1.42
Tanaka 2022, 23–46 g/day, Men 0.90 [0.73, 1.11] 4.02
Tanaka 2022, 23–46 g/day, Women 1.21 [0.82, 1.78] 1.43
Heterogeneity: τ² = 0.00, I² = 94.30%, H² = 17.55 0.84 [0.80, 0.89]
Werstern countries
PHS 2005, ≥7 drinks/week, Men 0.75 [0.62, 0.91] 4.59
ILSA 2011, Men, 12–24 g/day 0.62 [0.21, 1.83] 0.19
ILSA 2011, Men, 25–47 g/day 0.29 [0.07, 1.15] 0.12
ILSA 2011, Women, 12–24 g/day 0.61 [0.28, 1.31] 0.38
ILSA 2011, Women, 25–47 g/day 1.27 [0.57, 2.81] 0.36
PREVEND 2015, 70–210 g/week, Men & Women 0.56 [0.38, 0.82] 1.47
PREVEND 2015, >120 g/week, Men & Women 0.51 [0.25, 1.04] 0.45
ARIC 2020, 8–14 drinks/week, Men & Women 0.61 [0.49, 0.76] 3.74
ARIC 2020, ≥15 drinks/week, Men & Women 0.74 [0.58, 0.95] 3.10
Heterogeneity: τ² = 0.00, I² = 0.00%, H² = 1.00 0.68 [0.61, 0.76]
Overall 0.82 [0.78, 0.86]
Heterogeneity: τ² = 0.00, I² = 89.89%, H² = 9.89
Test of group differences: Q_b(1) = 11.60, p = 0.00
0.5
1.0
2.0
RR of eGFR <60 mL/min/1.73 m²

Figure S8a

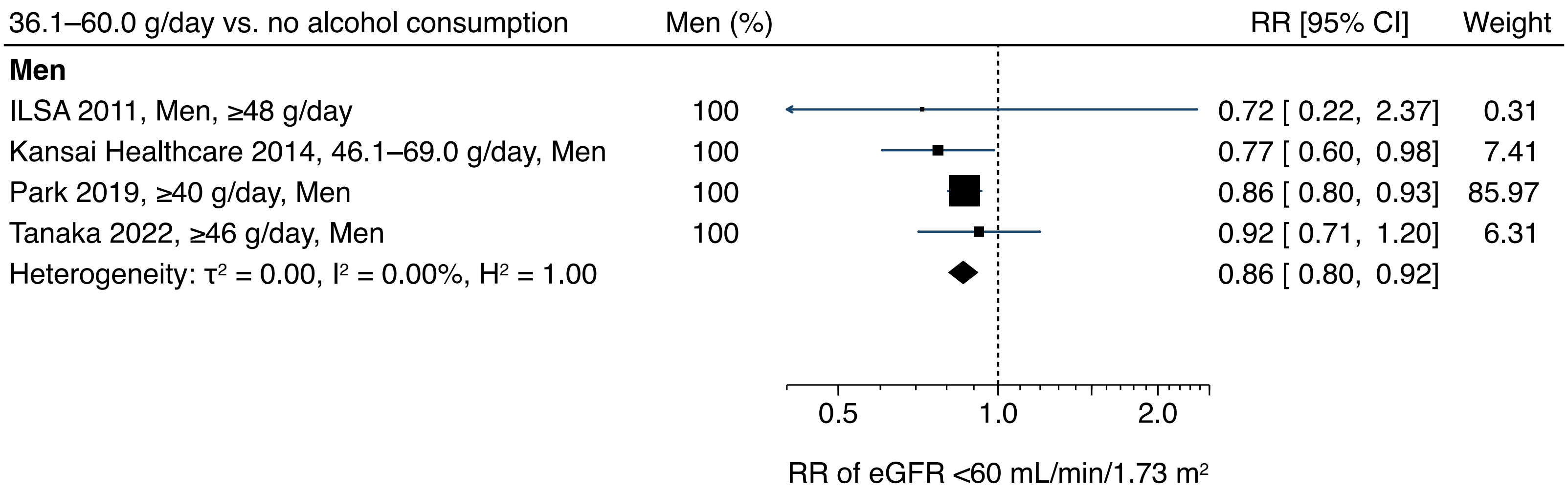
36.1–60.0 g/day vs. no alcohol consumption
Men (%)
RR [95% CI]
Weight
Men
ILSA 2011, Men, ≥48 g/day
100
0.72 [0.22, 2.37]
0.31
Kansai Healthcare 2014, 46.1–69.0 g/day, Men
100
0.77 [0.60, 0.98]
7.41
Park 2019, ≥40 g/day, Men
100
0.86 [0.80, 0.93]
85.97
Tanaka 2022, ≥46 g/day, Men
100
0.92 [0.71, 1.20]
6.31
Heterogeneity: τ^2 = 0.00, I^2 = 0.00%, H^2 = 1.00
0.86 [0.80, 0.92]
0.5
1.0
2.0
RR of eGFR <60 mL/min/1.73 m^2

Figure S8b

36.1–60.0 g/day vs. no alcohol consumption	Study size	RR [95% CI]	Weight
**Study size < Median**			
ILSA 2011, Men, ≥48 g/day	886	0.72 [0.22, 2.37]	1.33
Kansai Healthcare 2014, 46.1–69.0 g/day, Men	9112	0.77 [0.60, 0.98]	15.29
Tanaka 2022, ≥46 g/day, Women	7086	1.62 [1.04, 2.53]	7.36
Heterogeneity: $\tau^2 = 0.19$, $I^2 = 76.31\%$, $H^2 = 4.22$		1.01 [0.56, 1.83]	
**Study size ≥ Median**			
Park 2019, ≥40 g/day, Men	7625277	0.86 [0.80, 0.93]	26.14
Park 2019, ≥40 g/day, Women	6565601	0.74 [0.73, 0.76]	27.93
PROMISE 2021, ≥40 g/day, Men & Women	11175	1.40 [0.91, 2.15]	7.79
Tanaka 2022, ≥46 g/day, Men	19902	0.92 [0.71, 1.20]	14.16
Heterogeneity: $\tau^2 = 0.01$, $I^2 = 88.79\%$, $H^2 = 8.92$		0.86 [0.74, 1.00]	
**Overall**		0.89 [0.77, 1.03]	
Heterogeneity: $\tau^2 = 0.02$, $I^2 = 84.35\%$, $H^2 = 6.39$			
Test of group differences: $Q_b(1) = 0.27$, $p = 0.61$			

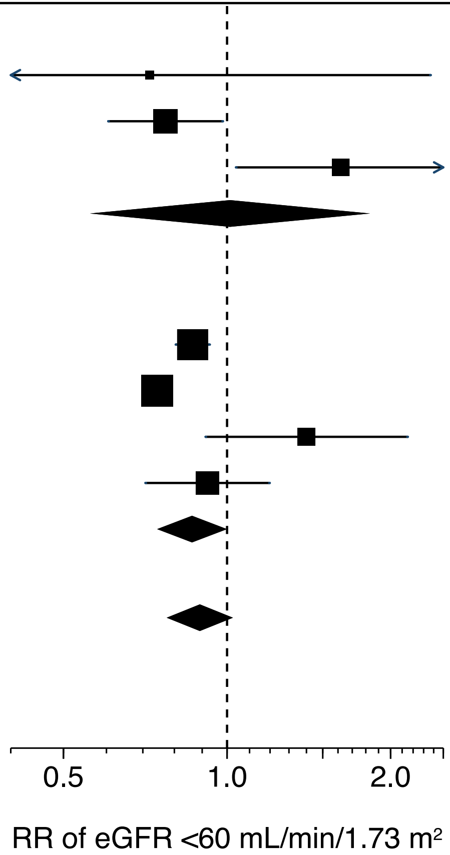

Figure S8c

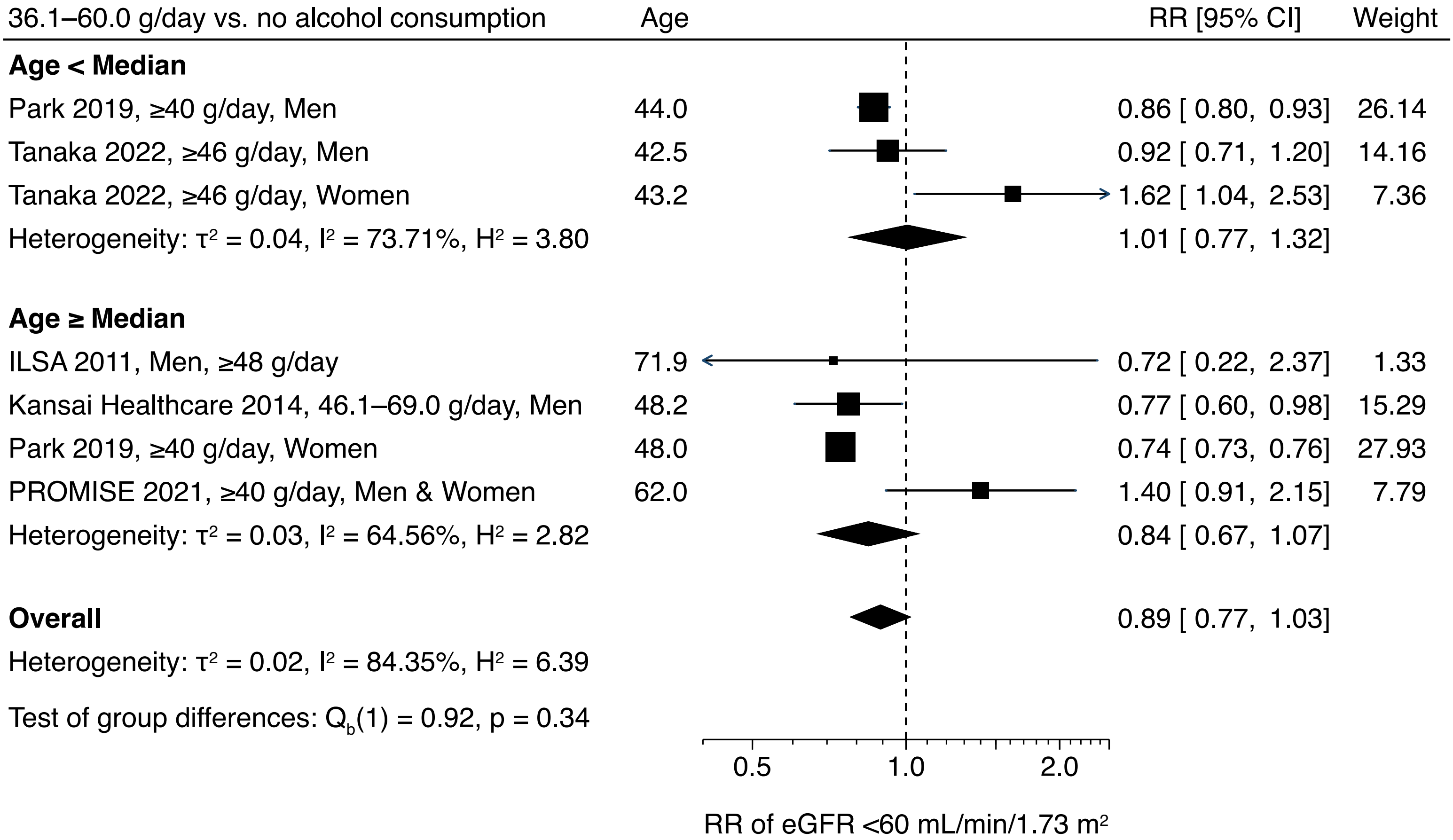
36.1–60.0 g/day vs. no alcohol consumption
Age
RR [95% CI]
Weight
Age < Median
Park 2019, ≥40 g/day, Men 44.0 0.86 [0.80, 0.93] 26.14
Tanaka 2022, ≥46 g/day, Men 42.5 0.92 [0.71, 1.20] 14.16
Tanaka 2022, ≥46 g/day, Women 43.2 1.62 [1.04, 2.53] 7.36
Heterogeneity: τ² = 0.04, I² = 73.71%, H² = 3.80 1.01 [0.77, 1.32]
Age ≥ Median
ILSA 2011, Men, ≥48 g/day 71.9 0.72 [0.22, 2.37] 1.33
Kansai Healthcare 2014, 46.1–69.0 g/day, Men 48.2 0.77 [0.60, 0.98] 15.29
Park 2019, ≥40 g/day, Women 48.0 0.74 [0.73, 0.76] 27.93
PROMISE 2021, ≥40 g/day, Men & Women 62.0 1.40 [0.91, 2.15] 7.79
Heterogeneity: τ² = 0.03, I² = 64.56%, H² = 2.82 0.84 [0.67, 1.07]
Overall 0.89 [0.77, 1.03]
Heterogeneity: τ² = 0.02, I² = 84.35%, H² = 6.39
Test of group differences: Qb(1) = 0.92, p = 0.34
0.5
1.0
2.0
RR of eGFR <60 mL/min/1.73 m²

Figure S8d

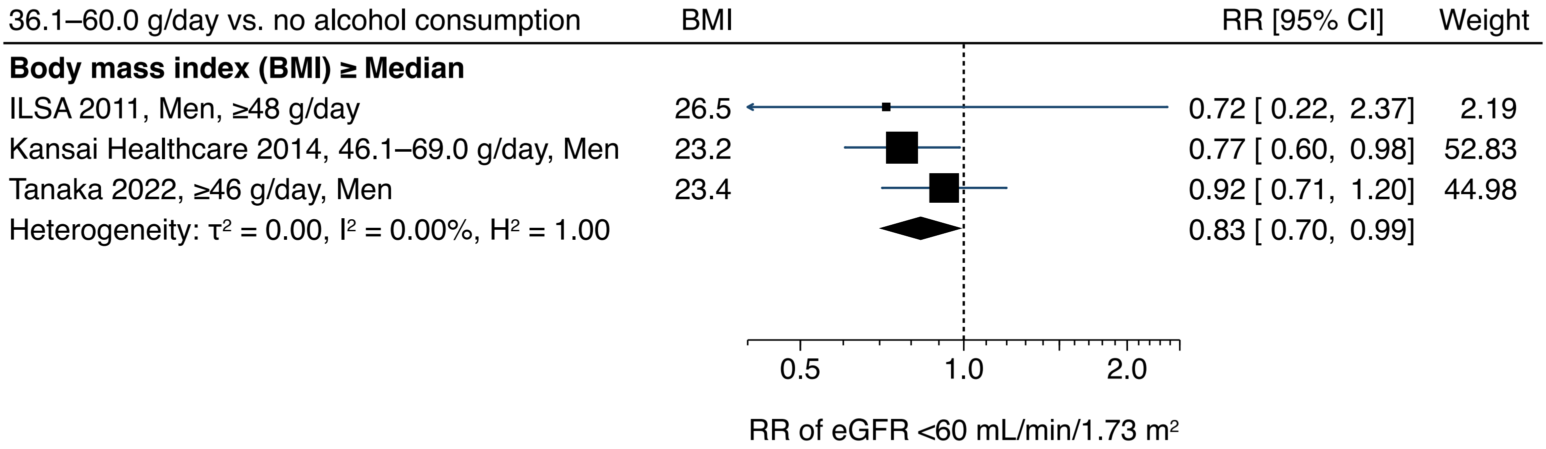
36.1–60.0 g/day vs. no alcohol consumption
BMI
RR [95% CI]
Weight
Body mass index (BMI) ≥ Median
ILSA 2011, Men, ≥48 g/day
26.5
0.72 [0.22, 2.37]
2.19
Kansai Healthcare 2014, 46.1–69.0 g/day, Men
23.2
0.77 [0.60, 0.98]
52.83
Tanaka 2022, ≥46 g/day, Men
23.4
0.92 [0.71, 1.20]
44.98
Heterogeneity: τ² = 0.00, I² = 0.00%, H² = 1.00
0.83 [0.70, 0.99]
0.5
1.0
2.0
RR of eGFR <60 mL/min/1.73 m²

Figure S8e

36.1–60.0 g/day vs. no alcohol consumption	eGFR	RR [95% CI]	Weight
**eGFR < Median**			
Kansai Healthcare 2014, 46.1–69.0 g/day, Men	84.7	0.77 [0.60, 0.98]	15.29
PROMISE 2021, ≥40 g/day, Men & Women	78.0	1.40 [0.91, 2.15]	7.79
Tanaka 2022, ≥46 g/day, Women	75.6	1.62 [1.04, 2.53]	7.36
Heterogeneity: $\tau^2 = 0.16$, $I^2 = 82.42\%$, $H^2 = 5.69$		1.17 [0.71, 1.94]	
**eGFR ≥ Median**			
Park 2019, ≥40 g/day, Men	91.7	0.86 [0.80, 0.93]	26.14
Park 2019, ≥40 g/day, Women	92.6	0.74 [0.73, 0.76]	27.93
Tanaka 2022, ≥46 g/day, Men	85.9	0.92 [0.71, 1.20]	14.16
Heterogeneity: $\tau^2 = 0.01$, $I^2 = 89.23\%$, $H^2 = 9.29$		0.82 [0.71, 0.94]	
**Overall**		0.90 [0.78, 1.03]	
Heterogeneity: $\tau^2 = 0.02$, $I^2 = 86.95\%$, $H^2 = 7.66$			
Test of group differences: $Q_b(1) = 1.82$, $p = 0.18$			

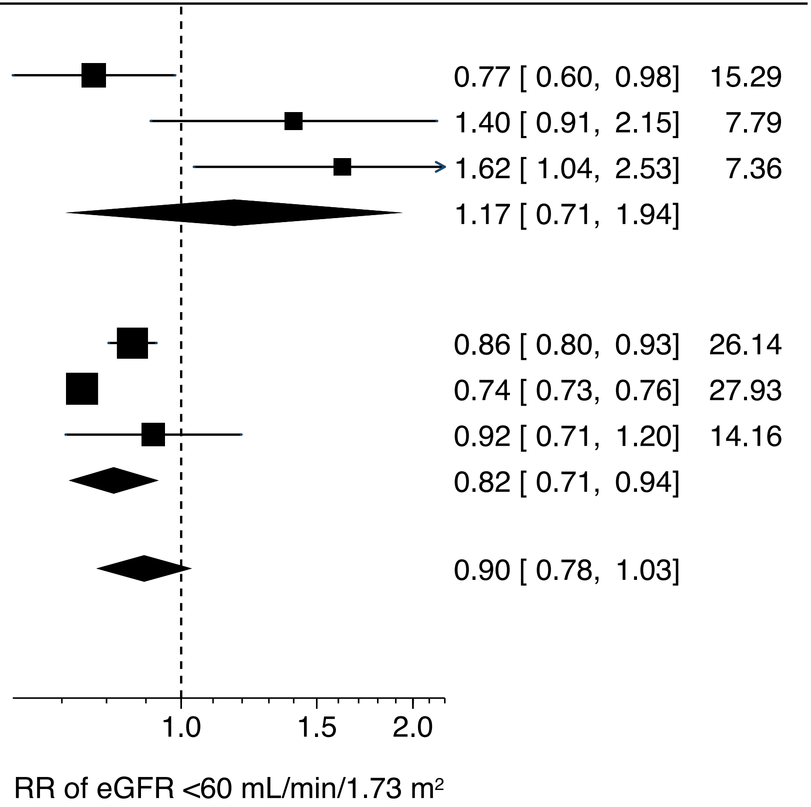

Figure S8f

36.1–60.0 g/day vs. no alcohol consumption	DM (%)	RR [95% CI]	Weight
**Prevalence of diabetes < Median**			
Kansai Healthcare 2014, 46.1–69.0 g/day, Men	0.0	0.77 [0.60, 0.98]	15.29
Tanaka 2022, ≥46 g/day, Men	3.1	0.92 [0.71, 1.20]	14.16
Tanaka 2022, ≥46 g/day, Women	1.3	1.62 [1.04, 2.53]	7.36
Heterogeneity: τ^2 = 0.07, I^2 = 75.89%, H^2 = 4.15		1.00 [0.70, 1.43]	
**Prevalence of diabetes ≥ Median**			
ILSA 2011, Men, ≥48 g/day	13.5	0.72 [0.22, 2.37]	1.33
Park 2019, ≥40 g/day, Men	3.9	0.86 [0.80, 0.93]	26.14
Park 2019, ≥40 g/day, Women	3.6	0.74 [0.73, 0.76]	27.93
PROMISE 2021, ≥40 g/day, Men & Women	3.4	1.40 [0.91, 2.15]	7.79
Heterogeneity: τ^2 = 0.01, I^2 = 87.73%, H^2 = 8.15		0.85 [0.72, 0.99]	
**Overall**		0.89 [0.77, 1.03]	
Heterogeneity: τ^2 = 0.02, I^2 = 84.35%, H^2 = 6.39			
Test of group differences: $Q_b(1)$ = 0.70, p = 0.40			

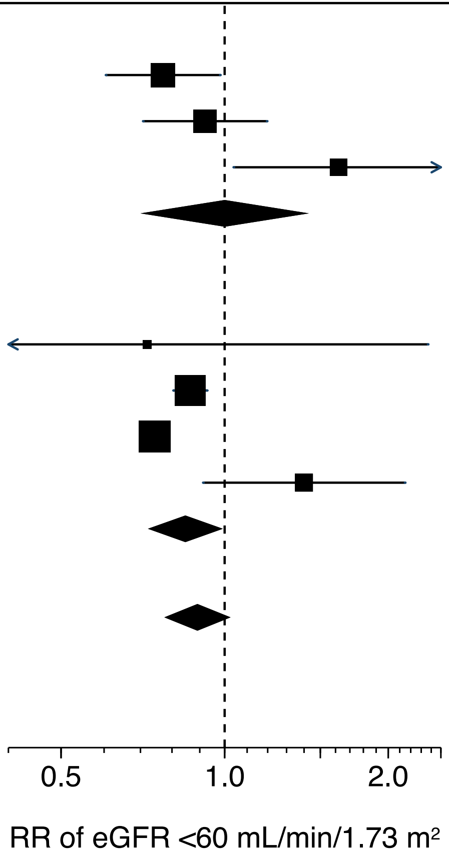

Figure S8g

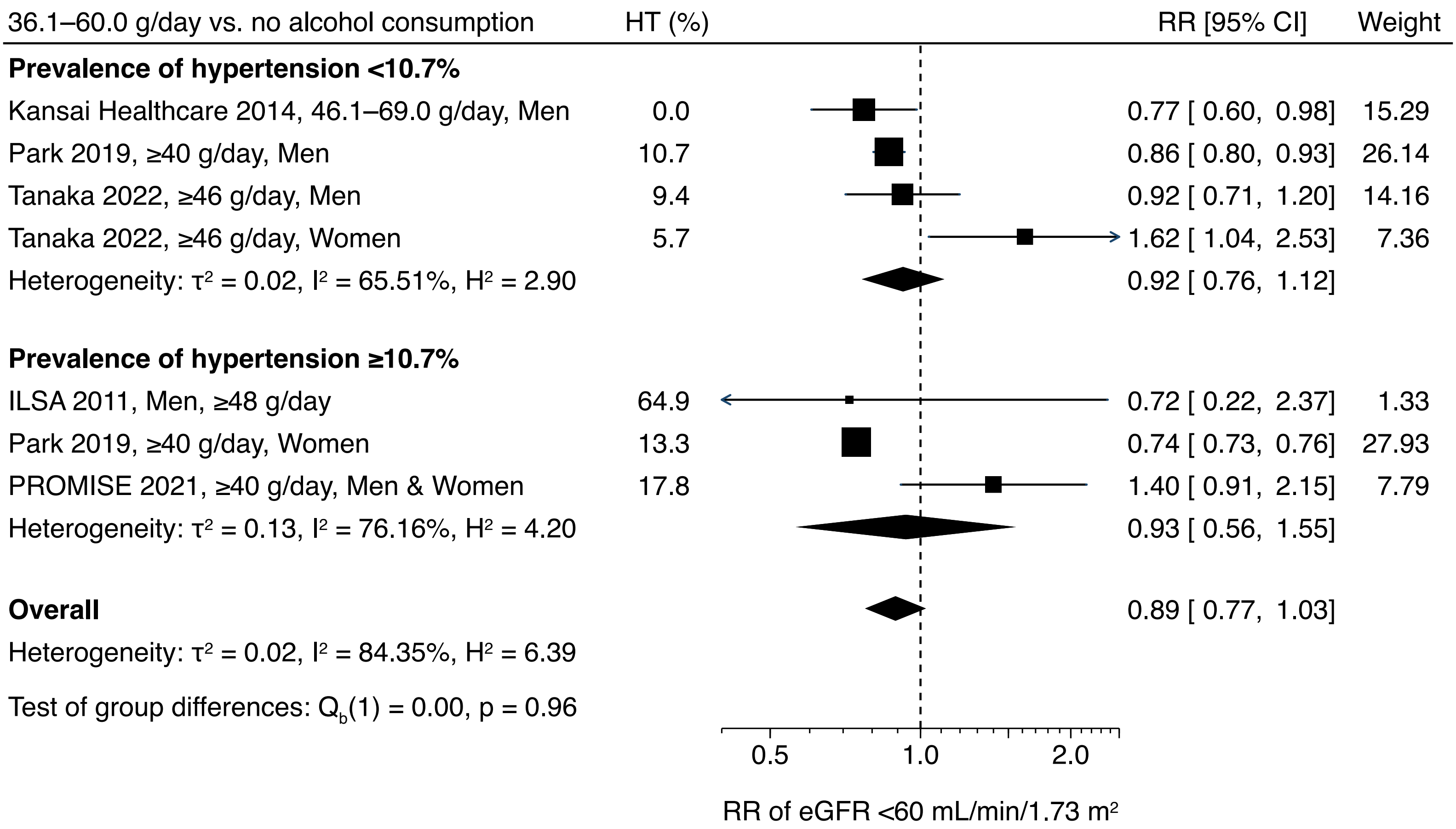
36.1–60.0 g/day vs. no alcohol consumption
HT (%)
RR [95% CI]
Weight
Prevalence of hypertension <10.7%
Kansai Healthcare 2014, 46.1–69.0 g/day, Men 0.0 0.77 [0.60, 0.98] 15.29
Park 2019, ≥40 g/day, Men 10.7 0.86 [0.80, 0.93] 26.14
Tanaka 2022, ≥46 g/day, Men 9.4 0.92 [0.71, 1.20] 14.16
Tanaka 2022, ≥46 g/day, Women 5.7 1.62 [1.04, 2.53] 7.36
Heterogeneity: τ^2 = 0.02, I^2 = 65.51%, H^2 = 2.90 0.92 [0.76, 1.12]
Prevalence of hypertension ≥10.7%
ILSA 2011, Men, ≥48 g/day 64.9 0.72 [0.22, 2.37] 1.33
Park 2019, ≥40 g/day, Women 13.3 0.74 [0.73, 0.76] 27.93
PROMISE 2021, ≥40 g/day, Men & Women 17.8 1.40 [0.91, 2.15] 7.79
Heterogeneity: τ^2 = 0.13, I^2 = 76.16%, H^2 = 4.20 0.93 [0.56, 1.55]
Overall 0.89 [0.77, 1.03]
Heterogeneity: τ^2 = 0.02, I^2 = 84.35%, H^2 = 6.39
Test of group differences: $Q_b(1)$ = 0.00, p = 0.96
0.5
1.0
2.0
RR of eGFR <60 mL/min/1.73 m^2

Figure S8h

36.1–60.0 g/day vs. no alcohol consumption	Follow-up	RR [95% CI]	Weight
**Follow-up duration <5 yr**			
ILSA 2011, Men, ≥48 g/day	3.5	0.72 [0.22, 2.37]	1.33
Tanaka 2022, ≥46 g/day, Men	4.0	0.92 [0.71, 1.20]	14.16
Tanaka 2022, ≥46 g/day, Women	4.0	1.62 [1.04, 2.53]	7.36
Heterogeneity: $\tau^2 = 0.09$, $I^2 = 60.13\%$, $H^2 = 2.51$		1.11 [0.70, 1.75]	
**Follow-up duration ≥5 yr**			
Kansai Healthcare 2014, 46.1–69.0 g/day, Men	8.7	0.77 [0.60, 0.98]	15.29
Park 2019, ≥40 g/day, Men	6.4	0.86 [0.80, 0.93]	26.14
Park 2019, ≥40 g/day, Women	6.4	0.74 [0.73, 0.76]	27.93
PROMISE 2021, ≥40 g/day, Men & Women	5.0	1.40 [0.91, 2.15]	7.79
Heterogeneity: $\tau^2 = 0.01$, $I^2 = 87.75\%$, $H^2 = 8.16$		0.83 [0.72, 0.96]	
**Overall**		0.89 [0.77, 1.03]	
Heterogeneity: $\tau^2 = 0.02$, $I^2 = 84.35\%$, $H^2 = 6.39$			
Test of group differences: $Q_b(1) = 1.39$, $p = 0.24$			

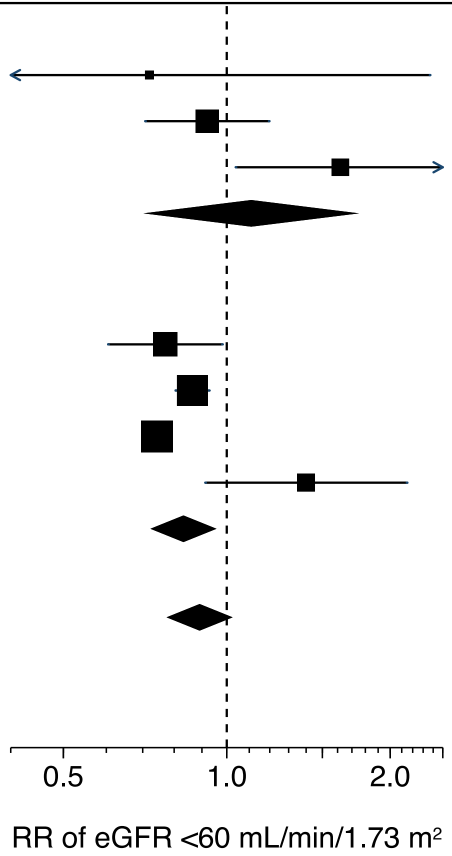

Figure S8i

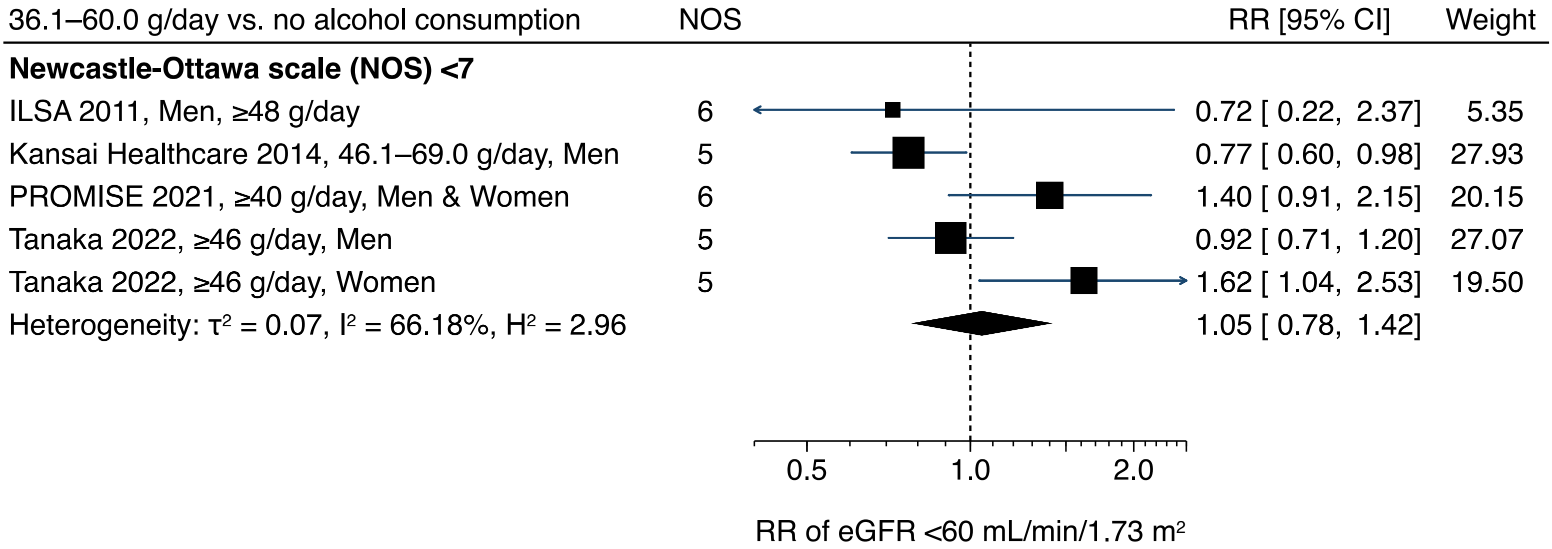
36.1–60.0 g/day vs. no alcohol consumption
NOS
RR [95% CI]
Weight
Newcastle-Ottawa scale (NOS) <7
ILSA 2011, Men, ≥48 g/day
6
0.72 [0.22, 2.37]
5.35
Kansai Healthcare 2014, 46.1–69.0 g/day, Men
5
0.77 [0.60, 0.98]
27.93
PROMISE 2021, ≥40 g/day, Men & Women
6
1.40 [0.91, 2.15]
20.15
Tanaka 2022, ≥46 g/day, Men
5
0.92 [0.71, 1.20]
27.07
Tanaka 2022, ≥46 g/day, Women
5
1.62 [1.04, 2.53]
19.50
Heterogeneity: τ² = 0.07, I² = 66.18%, H² = 2.96
1.05 [0.78, 1.42]
0.5
1.0
2.0
RR of eGFR <60 mL/min/1.73 m²

Figure S8j

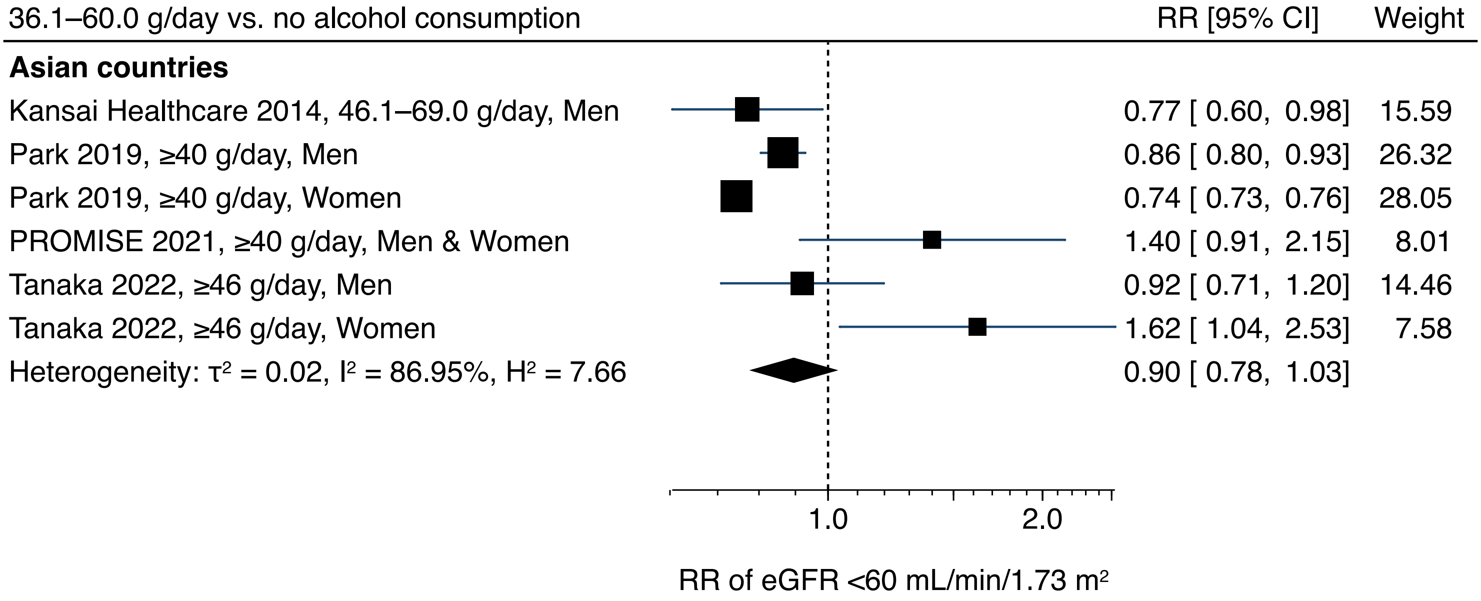
36.1–60.0 g/day vs. no alcohol consumption
RR [95% CI]
Weight
Asian countries
Kansai Healthcare 2014, 46.1–69.0 g/day, Men
0.77 [0.60, 0.98]
15.59
Park 2019, ≥40 g/day, Men
0.86 [0.80, 0.93]
26.32
Park 2019, ≥40 g/day, Women
0.74 [0.73, 0.76]
28.05
PROMISE 2021, ≥40 g/day, Men & Women
1.40 [0.91, 2.15]
8.01
Tanaka 2022, ≥46 g/day, Men
0.92 [0.71, 1.20]
14.46
Tanaka 2022, ≥46 g/day, Women
1.62 [1.04, 2.53]
7.58
Heterogeneity: τ² = 0.02, I² = 86.95%, H² = 7.66
0.90 [0.78, 1.03]
1.0
2.0
RR of eGFR <60 mL/min/1.73 m²
